# A one-pot electrochemical synthesis of 2-aminothiazoles from active methylene ketones and thioureas mediated by NH_4_I

**DOI:** 10.3762/bjoc.18.130

**Published:** 2022-09-15

**Authors:** Shang-Feng Yang, Pei Li, Zi-Lin Fang, Sen Liang, Hong-Yu Tian, Bao-Guo Sun, Kun Xu, Cheng-Chu Zeng

**Affiliations:** 1 Beijing Advanced Innovation Center for Food Nutrition and Human Health, Beijing Key Laboratory of Flavor Chemistry, Beijing Technology and Business University, Beijing 100048, China; 2 Faculty of Environment and Life, Beijing University of Technology, Beijing 100124, China

**Keywords:** 2-aminothiazoles, electrosynthesis, indirect electrolysis, halide ion

## Abstract

The electrochemical preparation of 2-aminothiazoles has been achieved by the reaction of active methylene ketones with thioureas assisted by ᴅʟ-alanine using NH_4_I as a redox mediator. The electrochemical protocol proceeds in an undivided cell equipped with graphite plate electrodes under constant current conditions. Various active methylene ketones, including β-keto ester, β-keto amide, β-keto nitrile, β-keto sulfone and 1,3-diketones, can be converted to the corresponding 2-aminothiazoles. Mechanistically, the in situ generated α-iodoketone was proposed to be the key active species.

## Introduction

**Scheme 1:**
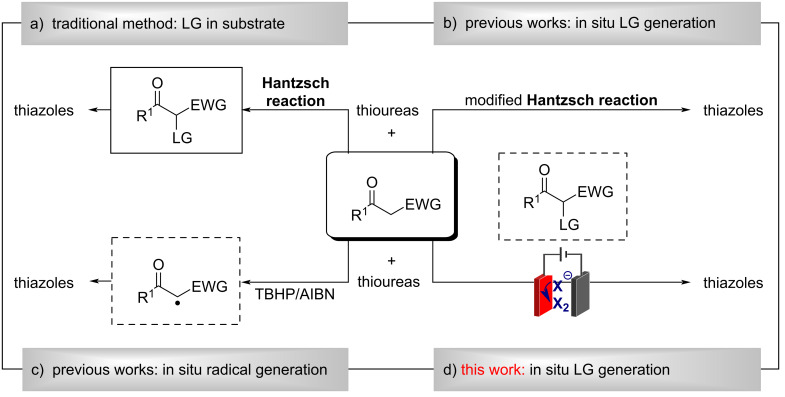
Methods for the synthesis of thiazoles using active methylene ketones as starting materials.

Thiazoles are prevalent structural motifs in a wide range of natural products [1] and synthetic molecules possessing various pharmaceutical activities such as antimicrobial [2–3], antiviral [4], antitumor [5–6], anti-inflammatory [7–8] and so on. Moreover, as a type of important intermediates, thiazole is of prime importance in organic synthesis [9–10] which is used extensively in the preparation of flavors [11], polymers [12], dyes [13], etc. These important features of thiazoles have driven intense interests in their facile synthesis [14–17]. Among various synthetic routes to the thiazole unit, the Hantzsch condensation of α-halo ketones (dielectrophiles) with various thioureas (dinucleophiles) should be the most well-known method (Scheme 1a) [18]. Since active methylene ketones are able to be in situ α-halogenated, the modified Hantzsch condensation of active methylene ketones with thioureas has attracted increasing attention in thiazoles’ synthesis, thereby saving costs and time needed to prepare the required α-halogenated dielectrophiles. Along this line, in situ α-halogenation strategies have been developed, using various halogenating reagents including Br_2_ [19–20], I_2_ [21–22], NBS [23–25], tribromoisocyanuric acid [26], 1,3-dichloro-5,5-dimethylhydantoin [27], HBr or HI, DMSO [28] etc. (Scheme 1b). Alternatively, the oxidation of α-C–H of active methylene ketones generate α-carbon-centered radicals, thus providing another way to obtain thiazoles. Recently, Sun et al. reported a *tert*-butyl hydroperoxide/azodiisobutyronitrile-mediated synthesis of 2-aminothiazoles from active methylene ketones and thiourea via an oxidative cyclization initiated by a radical process and a following condensation reaction (Scheme 1c) [29]. Although these methods are practical, most of these strategies require stoichiometric or excess amounts of halogenating reagents or oxidants, which are toxic, hazardous and would lead to a large quantity of waste. Considering the importance of thiazoles in synthetic and medicinal chemistry, the development of greener and more atom economic processes for the thiazole synthesis has become increasingly necessary with growing awareness of environmental constraints.

Organic electrosynthesis has been recognized as a green, modern, and safe technique, since electrons can be used as an alternative for oxidants or reductants [30–38]. During our continuous interests in halide-mediated indirect electrolysis [39–42], we have achieved the in situ generation of α-iodocarbonyl ketones from constant current electrolysis (CCE) of ketones in the presence of iodide ions. It is worth noting that we have also reported an electrochemical method for the synthesis of 2-aminothiazoles via the one-pot direct α-C–H functionalization of ketones with thioureas [42]. However, the reported method only tolerates aromatic and aliphatic ketones; the active methylene ketones were not suitable. Given that amino acids have been reported to work as green organocatalysts for the synthesis of 2-aminothiazole heterocycles [43–44]. We herein report a ᴅʟ-alanine-assisted one-pot electrochemical synthesis of 2-aminothiazoles from active methylene ketones and thioureas mediated by NH_4_I (Scheme 1d). This electrochemical method features external-oxidant-free conditions and avoids the prefunctionalization of the substrates.

## Results and Discussion

To demonstrate the feasibility of our idea, ethyl acetoacetate (**1a**) and thiourea (**2a**) were chosen as model substrates for the optimization of reaction conditions. Based on our previous studies on the halide-mediated α-C–H functionalization of carbonyl compounds [32–35], graphite was chosen as the working electrode rather than exploring other options. As shown in Table 1, when the electrolysis of **1a** with **2a** was performed in aqueous DMSO at a constant current of 5 mA/cm^2^ using NH_4_I as the mediator, LiClO_4_ as electrolyte and asparagine as additive in an undivided cell, the desired product **3a** was isolated in 51% after passing 6 F/mol of charge (Table 1, entry 1). The yield of **3a** decreased obviously when the ratio of **1a** to **2a** changed from 2:1 to 1:1 or 1:2 (Table 1, entries 2 and 3). Subsequent solvent screening disclosed that aqueous DMSO was the optimal solvent and lower yields were obtained in other solvents, such as aqueous DMF, aqueous MeCN or aqueous EtOH (Table 1, entries 4–6). Owing to the tedious workup process in using a large amount of DMSO as solvent, we decided to increase the proportion of H_2_O and disclosed that the yield of **3a** improved from 52% to 61% when the ratio of DMSO to H_2_O changed from 2:1 to 1:14 (Table 1, entry 7). When the reaction was performed in H_2_O, **3a** was obtained in 51% yield (Table 1, entry 8). It was observed that the additive plays an important role, among which ᴅʟ-alanine was proved to be the best, although asparagine, ʟ-phenylalanine, ʟ-aspartic acid and ʟ-allysine also gave comparable yields of **3a** (Table 1, entries 9–12). It is interesting to note that **3a** was achieved in higher yields (75%) when the temperature decreased from 70 °C to 30 °C (Table 1, entry 14). The eﬀect of current density was also examined. It was found that a slightly lower yield of **3a** was produced when 4 mA/cm^2^ (Table 1, entry 15) or 6 mA/cm^2^ (Table 1, entry 16) was used instead of the optimal 5 mA/cm^2^. In addition, 6 F/mol of charge was preferable because less yield of **3a** was obtained when 4 F/mol (Table 1, entry 17) and 8 F/mol (Table 1, entry 18) charge were consumed.

**Table 1:** Optimization of the reaction conditions^a^.

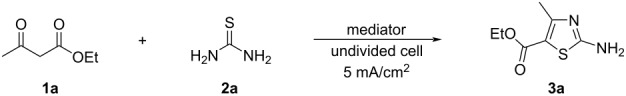							

Entry	**1a**:**2a**	Mediator	Solvent (15 mL)	Additive	*T* [°C]	F [mol]	Yield [%]^b^

1	2:1	NH_4_I	DMSO/H_2_O (2:1)	asparagine	70	6	51
2	1:1	NH_4_I	DMSO/H_2_O (2:1)	asparagine	70	6	17
3	1:2	NH_4_I	DMSO/H_2_O (2:1)	asparagine	70	6	15
4	2:1	NH_4_I	DMF/H_2_O (2:1)	asparagine	70	6	41
5	2:1	NH_4_I	MeCN/H_2_O (2:1)	asparagine	70	6	33
6	2:1	NH_4_I	EtOH/H_2_O (2:1)	asparagine	70	6	46
7	2:1	NH_4_I	DMSO/H_2_O (1:14)	asparagine	70	6	61
8	2:1	NH_4_I	H_2_O	asparagine	70	6	51
9	2:1	NH_4_I	DMSO/H_2_O (1:14)	ʟ-phenylalanine	70	6	60
10	2:1	NH_4_I	DMSO/H_2_O (1:14)	ʟ-aspartic acid	70	6	62
11	2:1	NH_4_I	DMSO/H_2_O (1:14)	ʟ-allysine	70	6	60
12	2:1	NH_4_I	DMSO/H_2_O (1:14)	ᴅʟ-alanine	70	6	65
13	2:1	NH_4_I	DMSO/H_2_O (1:14)	ᴅʟ-alanine	50	6	65
14	2:1	NH_4_I	DMSO/H_2_O (1:14)	ᴅʟ-alanine	30	6	75
15^c^	2:1	NH_4_I	DMSO/H_2_O (1:14)	ᴅʟ-alanine	30	6	65
16^d^	2:1	NH_4_I	DMSO/H_2_O (1:14)	ᴅʟ-alanine	30	6	73
17	2:1	NH_4_I	DMSO/H_2_O (1:14)	ᴅʟ-alanine	30	4	66
18	2:1	NH_4_I	DMSO/H_2_O (1:14)	ᴅʟ-alanine	30	8	75
19	2:1	NH_4_Cl	DMSO/H_2_O (1:14)	ᴅʟ-alanine	30	6	35
20	2:1	NH_4_Br	DMSO/H_2_O (1:14)	ᴅʟ-alanine	30	6	55
21	2:1	Et_4_NI	DMSO/H_2_O (1:14)	ᴅʟ-alanine	30	6	64
22	2:1	Bu_4_NI	DMSO/H_2_O (1:14)	ᴅʟ-alanine	30	6	59
23	2:1	NaI	DMSO/H_2_O (1:14)	ᴅʟ-alanine	30	6	72
24^e^	2:1	NH_4_I	DMSO/H_2_O (1:14)	ᴅʟ-alanine	30	6	73
25^f^	2:1	NH_4_I	DMSO/H_2_O (1:14)	ᴅʟ-alanine	30	6	70

^a^Reaction conditions: ethyl acetoacetate (**1a**, 2 mmol), thiourea (**2a**, 1 mmol), NH_4_I (1 mmol), acid (1 mmol), LiClO_4_ (0.5 mmol), DMSO/H_2_O (v/v), undivided cell, graphite plate anode and cathode, 5 mA/cm^2^. ^b^Isolated yield. ^c^4 mA/cm^2^. ^d^6 mA/cm^2^. ^e^NH_4_I (0.1 mmol). ^f^Without LiClO_4_.

Next, the effect of halide-based mediators on the reaction was examined. When NH_4_Cl or NH_4_Br was used as the mediator, the yield of **3a** decreased to 35% (Table 1, entry 19) and 55% (Table 1, entry 20), respectively. Slightly lower yields of **3a** were obtained when the mediator NH_4_I was replaced by Et_4_NI, Bu_4_NI or NaI (Table 1, entries 21–23). Interestingly, almost same yield of **3a** was isolated when the amount of NH_4_I was reduced to 0.1 mmol (Table 1, entry 24). In addition, without conductive salt (LiClO_4_) the electrochemical reaction also proceeded smoothly with a comparable yield (Table 1, entry 25).

Based on the results mentioned above, the optimal reaction conditions are as follows: constant current electrolysis was performed in an undivided cell equipped with a graphite plate as working electrode and counter electrode, using 0.1 mmol of NH_4_I as the mediator and DMSO/H_2_O (1 mL + 14 mL) as the solvent in the presence of ᴅʟ-alanine (Table 1, entry 24).

**Scheme 2:**
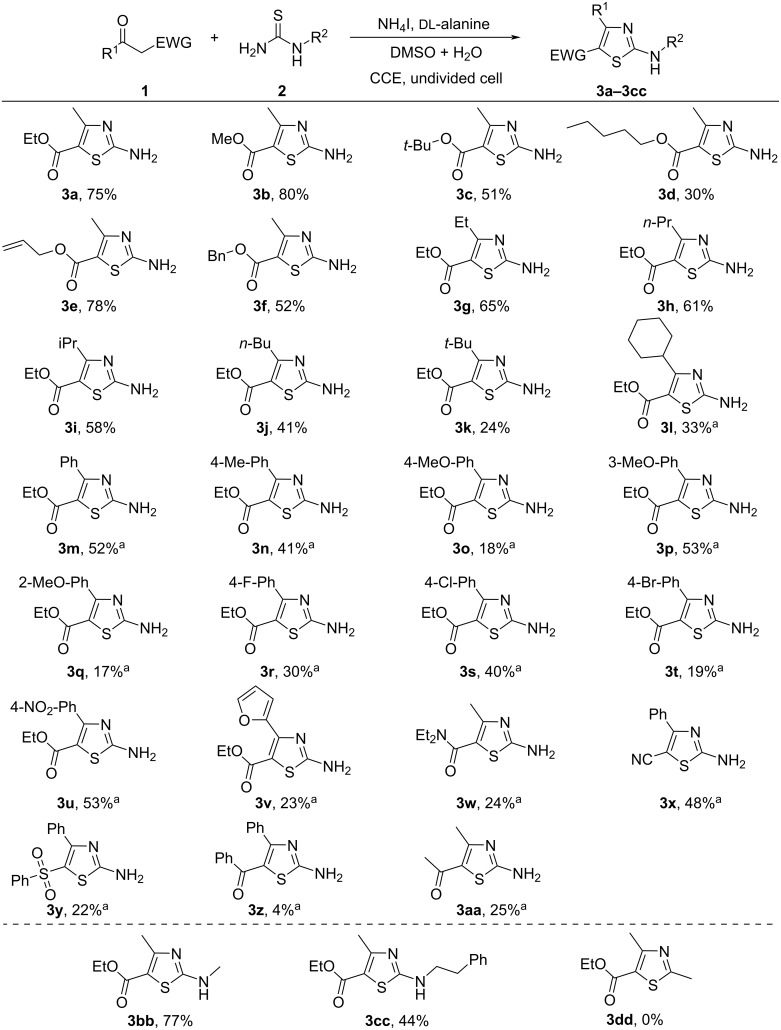
Substrate scope. Reaction conditions: **1** (2 mmol), **2** (1 mmol), NH_4_I (0.1 mmol), ᴅʟ-alanine (1 mmol), LiClO_4_ (0.5 mmol) in DMSO 1 mL + H_2_O 14 mL, undivided cell, graphite plate anode and cathode, 30 °C, 5 mA/cm^2^, 6 F/mol; isolated yields are given. ^a^8 F/mol.

Under the optimized reaction conditions (entry 24, Table 1), the scope of the electrochemical reaction was studied using a series of active methylene ketones (Scheme 2). Various linear and branched alkyl acetoacetates including methyl, ethyl, *tert*-butyl, and amyl reacted smoothly with thiourea **2a** under the optimized conditions, giving the corresponding products in 30–80% yields (**3a**–**d**). In addition, allyl and benzyl acetoacetate were also suitable substrates to give the desired 2-aminothiazoles **3e** and **3f** in 78% and 52% yields, respectively. β-ketoesters containing ethyl, *n*-propyl, isopropyl, *n*-butyl, *tert-b*utyl and cyclohexyl moieties were also compatible with the optimized conditions, providing the corresponding 2-aminothiazoles in 24% to 65% yields (**3g–l**).

As shown in Scheme 2, when the R^1^ group changed from alkyl to aryl, the target products could also be obtained. For example, ethyl 3-oxo-3-phenylpropanoate and ethyl 3-oxo-3-(*p*-tolyl)propanoate afforded the corresponding 2-aminothiazoles **3m** and **3n** in 52% and 41% isolated yields under the standard conditions. When an electron-donating methoxy substituent was introduced to the *ortho-*, *meta*-, or *para*-position of the phenyl moiety, the corresponding 2-aminothiazoles, **3o**–**q**, were given in 17–53% yields. With electron-withdrawing groups (F, Cl, Br, NO_2_), the desired products **3r–u** were obtained in moderate yields. Other heteroaryl ketones, ethyl 3-(furan-2-yl)-3-oxopropanoate (**1v**) reacted with thiourea to give **3v** in 23% yield. In addition to β-keto esters, other active methylene derivatives, including β-ketoamide, β-keto nitrile and β-keto sulfone were also suitable coupling partners with relatively lower yields (**3w–y**) and most of the starting materials were recovered. The method could also be applied to 1,3-diones. For example, in the cases of 1,3-diphenylpropane-1,3-dione and acetoacetone, the corresponding products **3z** and **3aa** were given in 4% and 25% yields, respectively.

Different *N*-substituted thioureas were also screened for the electrochemical reactions. *N*-methylthiourea and *N*-(2-phenylethyl)thiourea reacted smoothly with ethyl acetoacetate **1a** under the optimized conditions, providing the corresponding 2-aminothiazoles, **3bb** and **3cc**, in 77% and 44% yields, respectively. It is regretful that thioacetamide is not the best suitable partner due to its low reactivity under the optimized conditions.

**Scheme 3:**
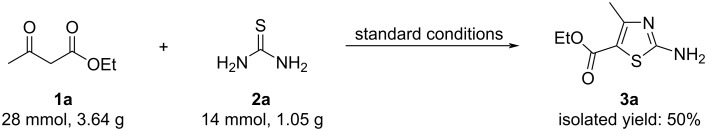
Up-scaling experiment.

To demonstrate the practicability of the reaction, a scale-up reaction of ethyl acetoacetate (**1a**, 28 mmol, 3.64 g) and thiourea (**2a**,14 mmol, 1.05 g) was carried out under the optimized conditions to give **3a** in 50% yield (Scheme 3).

**Scheme 4:**
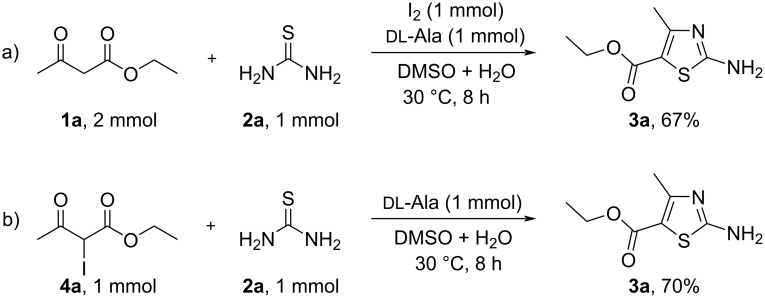
Control experiments.

In order to better understand the iodide-mediated reaction mechanism and to determine the possible active intermediates involved, several control experiments were carried out. As shown in Scheme 4, when molecular iodine was employed as oxidant, the desired product **3a** was obtained in a 67% yield under otherwise identical conditions (Scheme 4a), but without passing electricity. Therefore, the in situ-generated I_2_ was one of the active species. Further investigation revealed that the condensation of ethyl 2-iodo-3-oxobutanoate (**4a**) with thiourea **2a** could give the target product **3a** in 70% yield (Scheme 4b). Therefore, the in situ-generated ethyl 2-iodo-3-oxobutanoate (**4a**) should be a key intermediate for this tandem reaction.

**Scheme 5:**
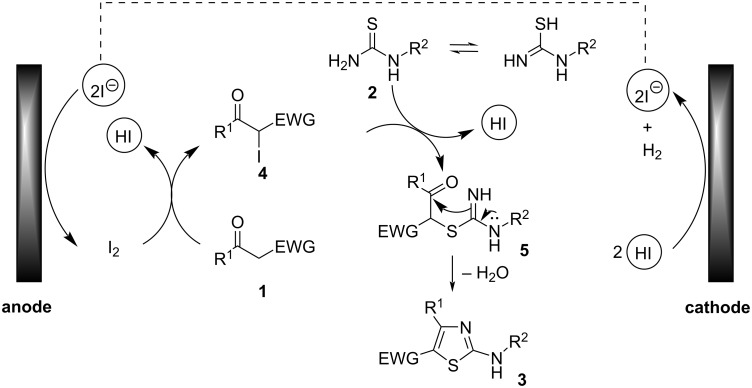
The proposed mechanism for the one-pot electrochemical synthesis of 2-aminothiazoles mediated by NH_4_I.

On the basis of the above mechanistic studies and the previous works on iodide-mediated electrochemical transformation [37–40], a possible mechanism for this electrochemical reaction was proposed (Scheme 5). It is well known that amino acid can act as a bi-functional organocatalyst due to the existence of both Lewis base (NH_2_) and Brønsted acidic (COOH) sites. In the suggested mechanism, the carboxy group may polarize the carbonyl group of the active methylene ketone and the amino group as a Lewis base serves the formation of enolate to produce α-iodo ketone with the molecular I_2_ produced by anodic oxidation. Subsequently, the nucleophilic substitution between intermediate **4** and thiourea tautomer gives α-sulfur substituted ketone **5**. Intermediate **5** undergoes intramolecular nucleophilic addition to the carbonyl group and followed by dehydration to give the heterocyclic product **3**. At the cathode, protons are reduced to release H_2_.

## Conclusion

In conclusion, we have developed a one-pot electrochemical strategy for the synthesis of 2-amniothiazoles by the reaction of active methylene ketones with thioureas. The electrochemical synthesis was performed in an undivided cell with NH_4_I as the mediator and cheap graphite plate as the working electrode. Various active methylene ketones, including β-keto ester, β-keto amide, β-keto nitrile, β-keto sulfone and 1,3-diones proved to be compatible with the protocol. Since external oxidants and prefunctionalization of substrates are avoided, the present electrochemical protocol represents an appealing alternative for the synthesis of 2-aminothiazoles. Gram-scale synthesis also highlighted the synthetic practicability of this electrochemical strategy. Mechanistically, the in situ-generated α-iodoketone was proposed to be a key active species. Further application of this method is underway in our laboratory.

## Supporting Information

File 1 Experimental procedures, characterization data and copies of spectra of the all synthesized compounds (^1^H NMR, ^13^C NMR and HRMS).
