# Supplementary material for: A one-pot electrochemical synthesis of 2-aminothiazoles from active methylene ketones and thioureas mediated by NH4I
Source: Beilstein J Org Chem. 2022 Sep 15;18:1249–55. doi: 10.3762/bjoc.18.130 (PMC9490072; doi:10.3762/bjoc.18.130)
Supplement: File 1 — Experimental procedures, characterization data and copies of spectra of the all synthesized compounds (1H NMR, 13C NMR and HRMS). [file Beilstein_J_Org_Chem-18-1249-s001.pdf]

BEILSTEIN JOURNAL OF ORGANIC CHEMISTRY

Supporting Information

for

A one-pot electrochemical synthesis of 2-aminothiazoles from active methylene ketones and thioureas mediated by NH_4I

Shang-Feng Yang, Pei Li, Zi-Lin Fang, Sen Liang, Hong-Yu Tian, Bao-Guo Sun, Kun Xu and Cheng-Chu Zeng

Beilstein J. Org. Chem. **2022,** *18,* 1249–1255. doi:10.3762/bjoc.18.130

Experimental procedures, characterization data and copies of spectra of the all synthesized compounds (^{1}H NMR, ^{13}C NMR and HRMS)

License and Terms: This is a supporting information file under the terms of the Creative Commons Attribution License (https://creativecommons.org/licenses/by/4.0). Please note that the reuse, redistribution and reproduction in particular requires that the author(s) and source are credited and that individual graphics may be subject to special legal provisions.
The license is subject to the *Beilstein Journal of Organic Chemistry* terms and conditions: (https://www.beilstein-journals.org/bjoc/terms)

Table of contents for the supporting information

1. General information S1

2. General procedure for the one-pot electrochemically synthesis of 2-aminothiazoles from active methylene ketones and thioureas............................... S1

3. Compounds characterization... S2

4. References.. S6

5. Spectra of prepared compounds S7

1. General information

Starting materials and solvents were obtained from commercial sources and used without further purification. Chromatographic purification of products was accomplished by flash chromatography on silica gel (petroleum ether/EtOAc). The product spots on the thin layer chromatography (TLC) were identified/visualized by fluorescence quenching or by potassium permanganate. NMR spectra were recorded with a 300 MHz spectrometer (300 MHz ^{1}H frequency, 75 MHz ^{13}C frequency). Chemical shifts were referenced to residual undeuterated solvent peaks (note: DMSO-d_6: 2.50 ppm ^{1}H NMR, 40 ppm ^{13}C NMR). Coupling constants are reported in Hz. High resolution mass spectra (HRMS) were obtained on a SolariX mass spectrometer.

2. General procedure for the one-pot electrochemically synthesis of 2-aminothiazoles from active methylene ketones and thioureas

A 50 mL undivided cell was equipped with a graphite plate cathode and a graphite plate anode (each about 2 × 2 cm^2) which were connected to a DC regulated power supply. To the cell was added active methylene ketone **1** (2 mmol), thiourea **2** (1 mmol), NH_4I (0.1 mmol), DL-alanine (1 mmol) and $LiClO_4$ (0.5 mmol) dissolved in a mixed solvent of DMSO (1 mL) and H_2O (14 mL). The mixture was electrolyzed under constant current conditions at 5 mA/cm^2 at 30 °C while stirring. The electrolysis was terminated when 6 F/mol of charge had been consumed. After the electrolysis, the reaction mixture was washed with a saturated aqueous $Na_2S_2O_3$ and the product was then extracted with DCM (3 × 10 mL), dried over $MgSO_4$, and concentrated in vacuum. The residue was purified by column chromatography on silica gel using a mixture of petroleum ether/EtOAc as eluent.

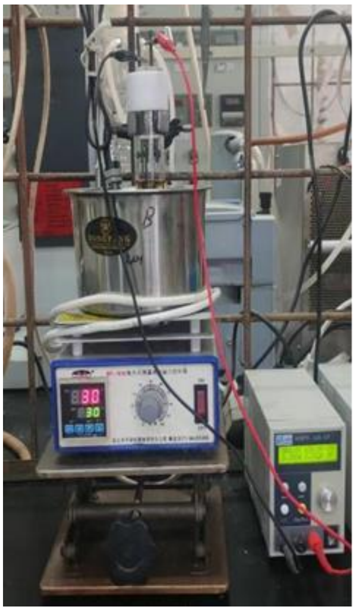

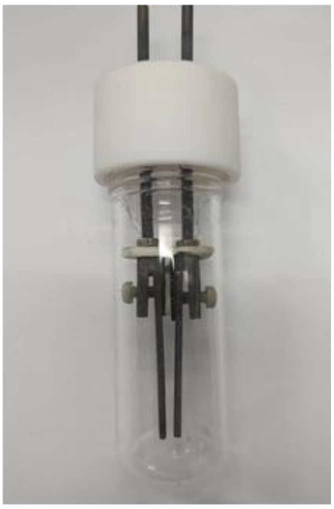

3. Compounds characterization

ethyl 2-amino-4-methylthiazole-5-carboxylate (**3a**)[1]	
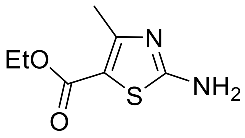	Yield: 140 mg, 75%; white solid; M.P.: 173-174 °C<br>^{1}H NMR (300 MHz, DMSO-d_6) δ (ppm): 1.22 (t, *J* = 7.2 Hz, 3H), 2.37 (s, 3H), 4.14 (q, *J* = 7.2 Hz, 2H), 7.72(br, 2H);<br>^{13}C NMR (75 MHz, DMSO-d_6) δ (ppm): 14.8, 17.6, 60.2, 107.9, 159.8, 162.5, 170.7.
methyl 2-amino-4-methylthiazole-5-carboxylate (**3b**)[2]	
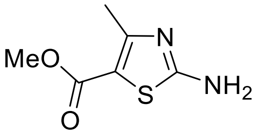	Yield: 138 mg, 80%; light yellow solid; M.P.: 218-220 °C.<br>^{1}H NMR (300 MHz, DMSO-d_6) δ (ppm): 2.37 (s, 3H), 3.67 (s, 3H), 7.74 (br, 2H);<br>^{13}C NMR (75 MHz, DMSO-d_6) δ (ppm): 17.6, 51.7, 107.3, 160.1, 162.8, 170.8.
tert-butyl 2-amino-4-methylthiazole-5-carboxylate (**3c**) [1]	
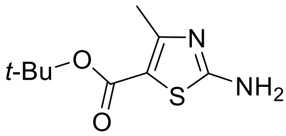	Yield: 109 mg, 51%; yellow solid; M.P.: 162-163 °C.<br>^{1}H NMR (300 MHz, DMSO-d_6) δ (ppm): 1.45(s, 9H), 2.33(s, 3H), 7.62(br, 2H);<br>^{13}C NMR (75 MHz, DMSO-d_6) δ (ppm): 17.6, 28.5, 80.6, 109.7, 158.8, 162.0, 170.3.
pentyl 2-amino-4-methylthiazole-5-carboxylate (**3d**)[2]	
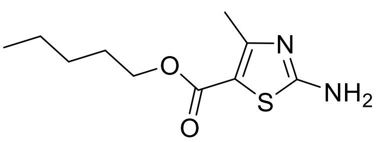	Yield: 52 mg,30%; yellow waxy solid.<br>^{1}H NMR (300 MHz, DMSO-d_6) δ (ppm): 0.87 (t, *J* = 6.6 Hz, 3H), 1.23-1.32 (m, 4H), 1.54-1.64 (m, 2H), 2.37(s, 3H), 4.09 (t, *J* = 6.6 Hz, 2H), 7.74 (br, 2H);<br>^{13}C NMR (75 MHz, DMSO-d_6) δ (ppm): 14.4, 17.7, 22.3, 28.3, 28.5, 64.3, 107.9, 159.8, 162.5, 170.8.
allyl 2-amino-4-methylthiazole-5-carboxylate (**3e**)[2]	
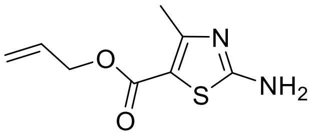	Yield: 155 mg, 78%; white solid; M.P.: 151-154 °C.<br>^{1}H NMR (300 MHz, DMSO-d_6) δ (ppm): 2.38 (s, 3H), 4.63-4.64 (m, 2H), 5.2-5.33 (m, 2H), 5.91-6.01 (m, 1H), 7.79 (br, 2H);<br>^{13}C NMR (75 MHz, DMSO-d_6) δ (ppm): 17.7, 64.6, 107.3,

	117.9, 133.5, 160.5, 162.0, 170.9.
benzyl 2-amino-4-methylthiazole-5-carboxylate (**3f**)	
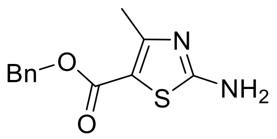	Yield: 130 mg, 52%; light yellow solid; M.P.: 135-138 °C.<br>^{1}H NMR (300 MHz, DMSO-d_6) δ (ppm): 2.38 (s, 3H), 5.19 (s, 2H), 7.31-7.39 (m, 5H), 7.78 (br, 2H);<br>^{13}C NMR (75 MHz, DMSO-d_6) δ (ppm): 17.7, 65.6, 107.3, 128.2, 128.4, 129.0, 137.0, 160.5, 162.2, 171.0;<br>HRMS (ESI) calcd for $C_{12}H_{13}N_2O_2S$ $(M+H)^+$ 249.06914, found 249.06923.
ethyl 2-amino-4-ethylthiazole-5-carboxylate (**3g**)	
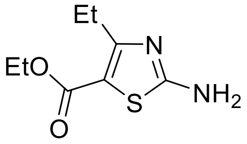	Yield: 127 mg, 65%; yellow solid; M.P.: 174-178 °C<br>^{1}H NMR (300 MHz, DMSO-d_6) δ (ppm): 1.11 (t, *J* = 7.5 Hz, 3H), 1.21 (t, *J* = 7.2 Hz, 3H), 2.82 (q, *J* = 6.3 Hz, 2H), 4.13 (q, *J* = 7.2 Hz, 2H), 7.75 (br, 2H);<br>^{13}C NMR (75 MHz, DMSO-d_6) δ (ppm): 13.9, 14.9, 24.2, 60.3, 107.2, 162.3, 165.5, 171.1;<br>HRMS (ESI) calcd for $C_8H_{13}N_2O_2S$ $(M+H)^+$ 201.06921, found 201.06923.
ethyl 2-amino-4-propylthiazole-5-carboxylate (**3h**)[1]	
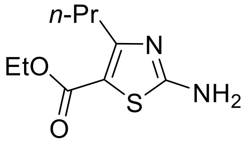	Yield: 130 mg, 61%; yellow solid; M.P.: 134-135 °C<br>^{1}H NMR (300 MHz, DMSO-d_6) δ (ppm): 0.87 (t, *J* = 7.5 Hz, 3H), 1.22 (t, *J* = 7.2 Hz, 3H), 1.52-1.62 (m, 2H), 2.80 (t, *J* = 7.5 Hz, 2H), 4.13 (q, *J* = 7.2 Hz, 2H), 7.73 (br, 2H);<br>^{13}C NMR (75 MHz, DMSO-d_6) δ (ppm): 14.3, 14.8, 22.4, 32.6, 60.3, 108.0, 162.3, 164.1, 170.9.
ethyl 2-amino-4-isopropylthiazole-5-carboxylate (**3i**)[3]	
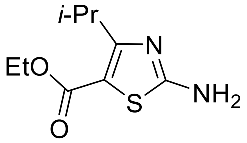	Yield: 114 mg, 58%; yellow solid; M.P.: 171-173 °C.<br>^{1}H NMR (300 MHz, DMSO-d_6) δ (ppm): 1.120 (d, *J* = 6.6 Hz, 6H), 1.213 (t, *J* = 6.9 Hz, 3H), 3.745-3.900 (m, 1H), 4.136 (q, *J* = 6.9 Hz, 2H), 7.776 (br s, 2H);<br>^{13}C NMR (75 MHz, DMSO-d_6) δ (ppm): 14.8, 22.4, 28.7, 60.2, 106.5, 162.2, 169.4, 171.2.
ethyl 2-amino-4-butylthiazole-5-carboxylate (**3j**)	
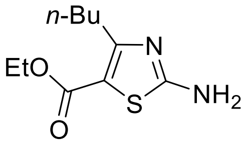	Yield: 95 mg, 41%; yellow solid; M.P.: 118-119 °C.<br>^{1}H NMR (300 MHz, DMSO-d_6) δ (ppm): 0.87 (t, *J* = 7.5 Hz, 3H), 1.22 (t, *J* = 7.2 Hz, 3H), 1.24-1.34 (m, 2H), 1.49-1.59 (m, 2H), 2.82 (t, *J* = 7.2 Hz, 2H), 4.13 (q, *J* = 7.2 Hz, 2H), 7.73 (br, 2H);<br>^{13}C NMR (75 MHz, DMSO-d_6) δ (ppm): 14.3, 14.8, 22.4, 30.2, 31.2, 60.2, 107.8, 162., 164.3, 170.9;<br>HRMS (ESI) calcd for $C_{10}H_{17}N_2O_2S$ $(M+H)^+$ 229.10046, found 229.10053.
ethyl 2-amino-4-(tert-butyl)thiazole-5-carboxylate (**3k**)	
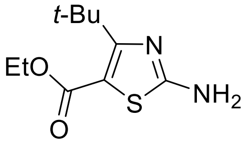	Yield: 51 mg, 24%; yellow waxy solid.<br>^{1}H NMR (300 MHz, DMSO-d_6) δ (ppm): 1.21 (t, *J* = 6.9 Hz, 3H), 1.34 (s, 9H), 4.12 (q, *J* = 6.9 Hz, 2H), 7.67 (br, 2H).<br>^{13}C NMR (75 MHz, DMSO-d_6) δ (ppm): 14.8, 29.6, 36.4, 60.4, 107.0, 161.6, 169.4, 170.7.<br>HRMS (ESI) calcd for $C_{10}H_{17}N_2O_2S$ $(M+H)^+$ 229.10036, found 229.10053.
ethyl 2-amino-4-cyclohexylthiazole-5-carboxylate (**3l**)	
	Yield: 84 mg, 33%; yellow solid; M.P.: 179-182 °C

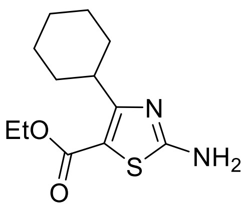	^{1}H NMR (300 MHz, DMSO-d_6) δ (ppm): 1.22 (t, *J* = 7.2 Hz, 3H), 1.29-1.76 (m, 10H), 3.40-3.50 (m, 1H), 4.13 (q, *J* = 6.9 Hz, 2H), 7.74 (br, 2H);<br>^{13}C NMR (75 MHz, DMSO-d_6) δ (ppm): 14.8, 26.2, 26.6, 32.2, 38.9, 60.2, 106.6, 162.2, 168.7, 171.1;<br>HRMS (ESI) calcd for $C_{12}H_{19}N_2O_2S$ $(M+H)^+$ 255.11599, found 255.11618.
ethyl 2-amino-4-phenylthiazole-5-carboxylate (**3m**)[3]	
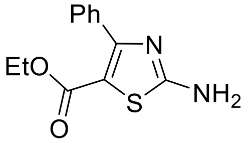	Yield: 130 mg, 52%; yellow solid; M.P.: 147-149 °C.<br>^{1}H NMR (300 MHz, DMSO-d_6) δ (ppm): 1.14 (t, *J* = 6.9 Hz, 3H), 4.08 (q, *J* = 6.9 Hz, 2H), 7.36-7.38 (m, 3H), 7.61-7.62 (m, 2H), 7.85 (br, 2H);<br>^{13}C NMR (75 MHz, DMSO-d_6) δ (ppm): 14.6, 60.6, 108.8, 127.8, 129.2, 130.2, 135.1, 159.3, 161.7, 170.4.
ethyl 2-amino-4-(p-tolyl) thiazole-5-carboxylate (**3n**)	
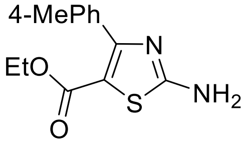	Yield: 107 mg, 41%; yellow solid; M.P.: 178-181°C.<br>^{1}H NMR (300 MHz, DMSO-d_6) δ (ppm): 1.15 (t, *J* = 7.2 Hz, 3H), 2.33 (s, 3H), 4.08 (q, *J* = 7.2 Hz, 2H), 7.17 (d, *J* = 7.8 Hz, 2H), 7.55 (d, *J* = 7.8 Hz, 2H), 7.84 (br, 2H);<br>^{13}C NMR (75 MHz, DMSO-d_6) δ (ppm): 14.6, 21.4, 60.4, 108.3, 128.3, 130.1, 132.2, 138.6, 159.3, 161.7, 170.2;<br>HRMS (ESI) calcd for $C_{13}H_{15}N_2O_2S$ $(M+H)^+$ 263.08464, found 263.08488.
ethyl 2-amino-4-(4-methoxyphenyl) thiazole-5-carboxylate (**3o**)	
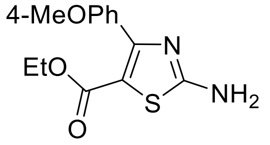	Yield: 51 mg, 18%; yellow solid; M.P.: 241-244 °C.<br>^{1}H NMR (300 MHz, DMSO-d_6) δ (ppm): 1.16 (t, *J* = 6.9 Hz, 3H), 3.79 (s, 3H), 4.09 (q, *J* = 6.0 Hz, 2H), 6.92 (d, *J* = 8.7 Hz, 2H), 7.65 (d, *J* = 8.4 Hz, 2H), 7.81 (br, 2H);<br>^{13}C NMR (75 MHz, DMSO-d_6) δ (ppm): 14.7, 55.6, 60.4, 107.6, 113.2, 127.3, 131.7, 159.1, 160.1, 161.8, 170.1;<br>HRMS (ESI) calcd for $C_{13}H_{15}N_2O_3S$ $(M+H)^+$ 279.07957, found 279.07979.
ethyl 2-amino-4-(3-methoxyphenyl) thiazole-5-carboxylate (**3p**)	
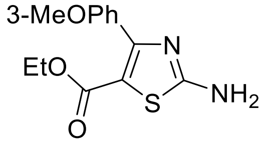	Yield: 147 mg, 53 %; yellow solid; M.P.: 286-304 °C.<br>^{1}H NMR (300 MHz, DMSO-d_6) δ (ppm): 1.14 (t, *J* = 6.9 Hz, 3H), 3.76 (s, 3H), 4.09 (q, *J* = 6.9 Hz, 2H), 6.94 (d, *J* = 6.9 Hz, 1H), 7.21-7.30 (m, 3H), 7.88 (br, 2H);<br>^{13}C NMR (75 MHz, DMSO-d_6) δ (ppm): 14.6, 55.6, 60.6, 109.1, 115.0, 115.5, 122.7, 128.9, 136.4, 158.9, 159.0, 161.7, 170.4;<br>HRMS (ESI) calcd for $C_{13}H_{15}N_2O_3S$ $(M+H)^+$ 279.07970, found 279.07979.
ethyl 2-amino-4-(2-methoxyphenyl) thiazole-5-carboxylate (**3q**)	
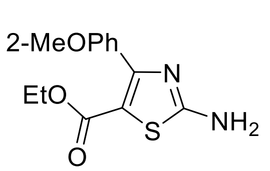	Yield: 50 mg, 17%; yellow waxy solid.<br>^{1}H NMR (300 MHz, DMSO-d_6) δ (ppm): 1.02 (t, *J* = 7.2 Hz, 3H), 3.68 (s, 3H), 3.97 (q, *J* = 7.2 Hz, 2H), 6.93 (t, *J* = 7.2 Hz, 1H), 7.01 (d, *J* = 8.4 Hz, 1H), 7.20 (d, *J* = 7.2 Hz, 1H), 7.32 (t, *J* = 7.2 Hz, 1H), 7.76 (br, 2H);<br>^{13}C NMR (75 MHz, DMSO-d_6) δ (ppm): 14.5, 55.7, 60.2, 110.8, 111.4, 120.1, 125.4, 130.1, 130.6, 155.8, 157.3, 161.6, 170.3;<br>HRMS (ESI) calcd for $C_{13}H_{15}N_2O_3S$ $(M+H)^+$ 279.07962, found 279.07979.
ethyl 2-amino-4-(4-fluorophenyl) thiazole-5-carboxylate (**3r**)	
	Yield: 79 mg, 30%; yellow solid; M.P.: 187-190 °C

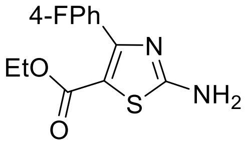	^{1}H NMR (300 MHz, DMSO-d_6) δ (ppm): 1.149 (t, *J* = 7.0 Hz, 3H), 4.089 (q, *J* = 7.1 Hz, 2H), 7.198 (t, *J* = 8.9 Hz, 2H), 7.671-7.719 (m, 2H), 7.881 (br, 2H);<br>^{13}C NMR (75 MHz, DMSO-d_6) δ (ppm): 14.6, 60.6, 108.7, 114.6 and 114.9(d, *J*(C,F) = 22.5 Hz),131.42 and 131.46(d, *J*(C,F)= 3.0 Hz), 132.4 and 132.5(d, *J*(C,F) = 7.5 Hz), 158.1 , 161.1 and 164.4 (d, *J*(C,F)= 247.5 Hz), 161.6, 170.4;<br>HRMS (ESI) calcd for $C_{12}H_{12}FN_2O_2S$ $(M+H)^+$ 267.05958, found 267.05980.
ethyl 2-amino-4-(4-chlorophenyl)thiazole-5-carboxylate (**3s**)[3]	
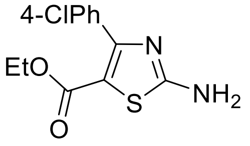	Yield: 112 mg, 40%; light yellow solid; M.P.: 188-193 °C
	^{1}H NMR (300 MHz, DMSO-d_6) δ (ppm): 1.15 (t, *J* = 6.9 Hz, 3H), 4.09 (q, *J* = 6.9 Hz, 2H), 7.44 (d, *J* = 8.4 Hz, 2H), 7.67 (d, *J* = 8.7 Hz, 2H), 7.90 (br, 2H);<br>^{13}C NMR (75 MHz, DMSO-d_6) δ (ppm): 14.6, 60.7, 109.1, 127.9, 132.0, 133.8, 133.8, 157.8, 161.6, 170.5.
ethyl 2-amino-4-(4-bromophenyl)thiazole-5-carboxylate (**3t**)[4]	
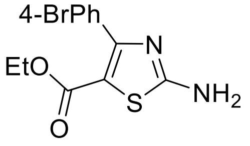	Yield: 62 mg, 19%; light yellow solid; M.P.: 187-202°C
	^{1}H NMR (300 MHz, DMSO-d_6) δ (ppm): 1.15 (t, *J* = 6.9 Hz, 3H), 4.09 (q, *J* = 6.9 Hz, 2H), 7.55-7.62 (m, 4H), 7.90 (br, 2H);<br>^{13}C NMR (75 MHz, DMSO-d_6) δ (ppm): 14.6, 60.7, 109.1, 122.5, 130.8, 132.2, 134.2, 157.8, 161.5, 170.5.
ethyl 2-amino-4-(4-nitrophenyl)thiazole-5-carboxylate (**3u**)[4]	
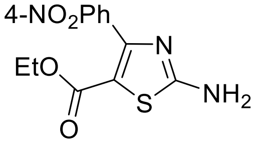	Yield: 155 mg, 53%; yellow waxy solid.
	^{1}H NMR (300 MHz, DMSO-d_6) δ (ppm): 1.15 (t, *J* = 6.9 Hz, 3H), 4.11 (q, *J* = 6.9 Hz, 2H), 7.90 (d, *J* = 8.7 Hz, 2H), 7.99 (br, 2H), 8.25 (d, *J* = 8.4 Hz, 2H);<br>^{13}C NMR (75 MHz, DMSO-d_6) δ (ppm): 14.5, 60.9, 113.0, 123.1, 131.5, 141.4, 147.7, 156.4, 161.4, 170.7.
ethyl 2-amino-4-(furan-2-yl)thiazole-5-carboxylate (**3v**)[4]	
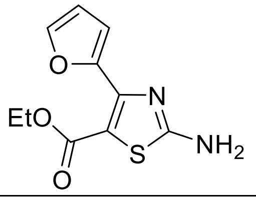	Yield: 55 mg, 23%; yellow waxy solid.
	^{1}H NMR (300 MHz, DMSO-d_6) δ (ppm): 1.23 (t, *J* = 6.9 Hz, 3H), 4.18 (q, *J* = 6.9 Hz, 2H), 6.60-6.62 (m, 1H), 7.48 (d, *J* = 3.3 Hz, 1H), 7.75 (s, 1H), 7.90 (br, 2H);<br>^{13}C NMR (75 MHz, DMSO-d_6) δ (ppm): 14.7, 60.8, 107.6, 112.2, 114.6, 144.0, 147.8, 148.5, 161.3, 170.2.
2-amino-N,N-diethyl-4-methylthiazole-5-carboxamide (**3w**)[2]	
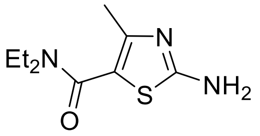	Yield: 51 mg, 24%; light yellow solid; M.P.: 148-154 °C.
	^{1}H NMR (300 MHz, DMSO-d_6) δ (ppm): 1.07 (t, *J* = 7.2 Hz ,6H), 2.05(s, 3H), 3.31-3.38 (m, 4H),7.19 (br, 2H);<br>^{13}C NMR (75 MHz, DMSO-d_6) δ (ppm): 14.1, 16.9, 41.4 111.4, 149.5, 163.7, 167.8.
2-amino-4-phenylthiazole-5-carbonitrile (**3x**)[5]	
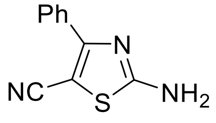	Yield: 95 mg, 48%; light yellow waxy solid.
	^{1}H NMR (300 MHz, DMSO-d_6) δ (ppm): 7.50-7.52 (m, 3H), 7.92 (d, *J* = 6.9 Hz, 2H), 8.26 (br, 2H)<br>^{13}C NMR (75 MHz, DMSO-d_6) δ (ppm): 84.1, 115.8, 127.9, 129.3, 130.5, 133.0, 161.5, 171.1.
4-phenyl-5-(phenylsulfonyl)thiazol-2-amine (**3y**)	
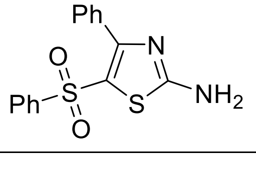	Yield: 67 mg, 22%; white solid; M.P.: 169-175 °C.
	^{1}H NMR (300 MHz, DMSO-d_6) δ (ppm): 7.37-7.56(m, 10H), 8.05 (br, 2H);<br>^{13}C NMR (75 MHz, DMSO-d_6) δ (ppm): 117.8, 127.0, 128.6, 130.2, 130.4, 133.7, 134.2, 143.0, 158.3, 171.6.

	HRMS (ESI) calcd for $C_{15}H_{13}N_2O_2S_2$ $(M+H)^+$ 317.04087, found 317.04130.
(2-amino-4-phenylthiazol-5-yl) (phenyl)methanone (**3z**) [6]	
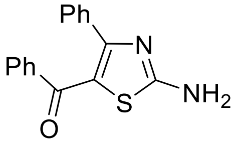	Yield: 12 mg, 4 %; light yellow solid; M.P.: 157-161 °C.
	^{1}H NMR (300 MHz, DMSO-d_6) δ (ppm): 7.04-7.14 (m, 5H), 7.22-7.29 (m, 3H), 7.35-7.37 (m, 2H), 8.06 (br, 2H); ^{13}C NMR (75 MHz, DMSO-d_6) δ (ppm): 121.0, 127.8, 128.1, 128.8, 129.0, 130.0, 131.6, 135.3, 139.0, 159.4, 171.4, 188.0.
1-(2-amino-4-methylthiazol-5-yl)ethan-1-one (**3aa**)[5]	
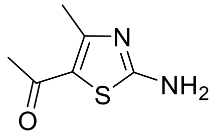	Yield: 39 mg, 25%; light yellow waxy solid.
	^{1}H NMR (300 MHz, DMSO-d_6) δ (ppm): 2.32 (s, 3H), 2.40 (s, 3H), 7.84 (br, 2H); ^{13}C NMR (75 MHz, DMSO-d_6) δ (ppm): 18.8, 30.0, 121.8, 158.1, 171.0, 188.8.
Ethyl 4-methyl-2-(methylamino)thiazole-5-carboxylate (**3bb**) [1]	
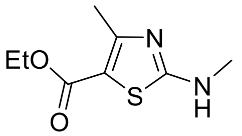	Yield: 154 mg, 77%; light yellow solid; M.P.: 149-151 °C.
	^{1}H NMR (300 MHz, DMSO-d_6) δ (ppm): 1.22 (t, *J* = 6.9 Hz, 3H), 2.41 (s, 3H), 2.82 (d, *J* = 4.8 Hz, 3H), 4.14 (q, *J* = 6.9 Hz, 2H), 8.28 (d, *J* = 4.2 Hz, 1H); ^{13}C NMR (75 MHz, DMSO-d_6) δ (ppm): 14.9, 17.8, 31.3, 60.3, 107.5, 160.2, 162.5, 171.2.
ethyl 4-methyl-2-(phenethylamino) thiazole-5-carboxylate (**3cc**)	
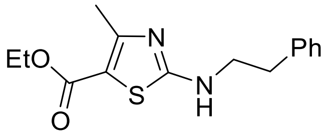	Yield: 128 mg, 44%; yellow solid; M.P.: 100-102 °C.
	^{1}H NMR (300 MHz, DMSO-d_6) δ (ppm): 1.22 (t, *J* = 6.9 Hz, 3H), 2.40 (s, 3H), 2.85 (t, *J* = 6.9 Hz, 2H), 3.45 (q, *J* = 6.6 Hz, 2H), 4.14 (q, *J* = 6.9 Hz, 2H), 7.23-7.30 (m, 5H), 8.44 (br ,1H); 13C NMR (75 MHz, DMSO-d_6) δ (ppm): 14.9, 17.9, 34.9, 46.2, 60.3, 107.4, 126.8, 128.9, 129.2, 139.5, 160.0, 162.5, 170.1; HRMS (ESI) calcd for $C_{15}H_{19}N_2O_2S$ $(M+H)^+$ 291.11602, found 291.11618.

4. References

[1] de Andrade, V. S. C.; de Mattos, M. C. S. d. *Synthesis* **2018**, 50, 4867-4874. doi:org/10.1055/s-0037-1610243.

[2] Roslan, I. I.; Ng, K. H.; Gondal, M. A.; Basheer, C.; Dastageer, M. A.; Jaenicke, S.; Chuah, G. K. *Adv. Synth. Catal.* **2018**, 360, 1584-1589. doi:org/10.1002/adsc.201701565.

[3] Narender, M.; Reddy, M. S.; Kumar, V. P.; Srinivas, B.; Sridhar, R.; Venkata, Y.; Nageswar, D.; Rao, K. R. *Synthesis* **2007**, 3469-3472. doi:org/10.1055/s-2007-990849.

[4] Zhu, Y P.; Yuan, J. J.; Zhao, Q.; Lian, M.; Gao, Q. H.; Liu, M. C.; Yang, Y.; Wu, A. X. *Tetrahedron* **2012**, 68, 173-178. doi:org/10.1016/j.tet.2011.10.074.

[5] Sun, J. Y.; Ge, H. B.; Zhen, X. H.; An, X. C.; Zhang, G. T.; Zhang-Negrerie, D.; Du, Y. F. Zhao, K. *Tetrahedron* **2018**, 74, 2107-2114. doi:org/10.1016/j.tet.2018.02.064.

[6] Zhao, J. W.; Xu, J. X.; Chen,J. X.; He, M. H.; Wang, X. Q. Tetrahedron **2015**,71 539-543. doi.org/10.1016/j.tet.2014.12.044

5. Spectra of prepared compounds

^{1}H NMR of ethyl 2-amino-4-methylthiazole-5-carboxylate (3a)

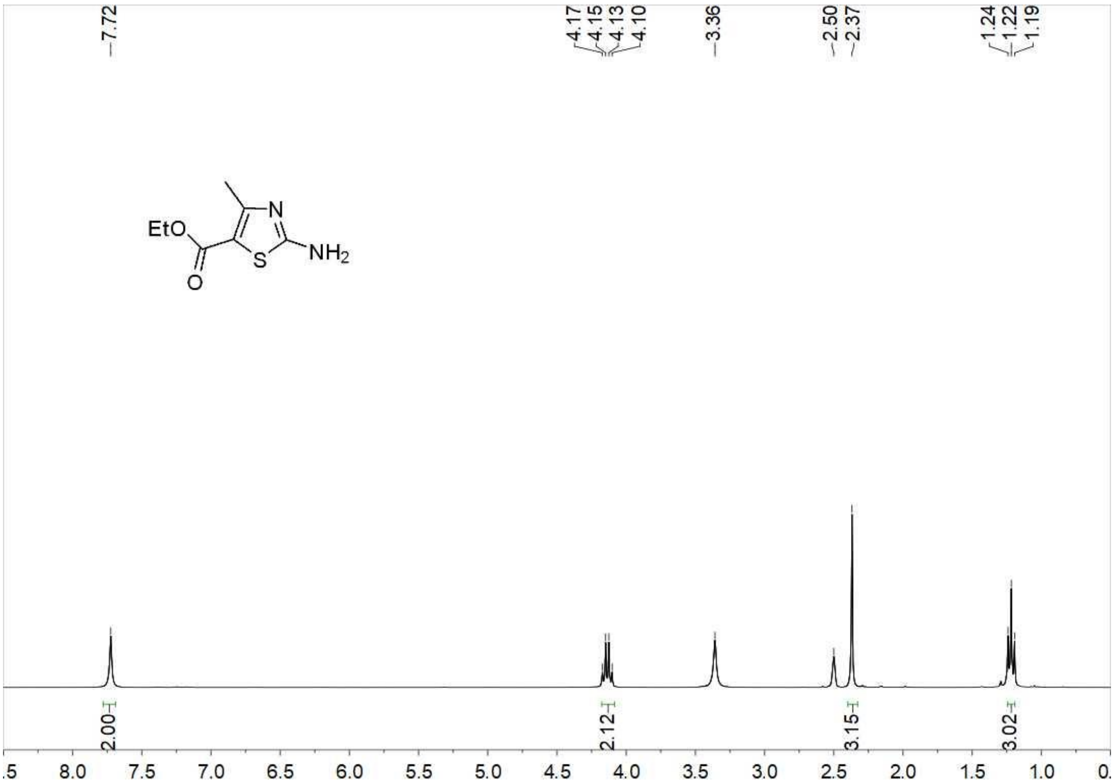

^{13}C NMR of ethyl 2-amino-4-methylthiazole-5-carboxylate (3a)

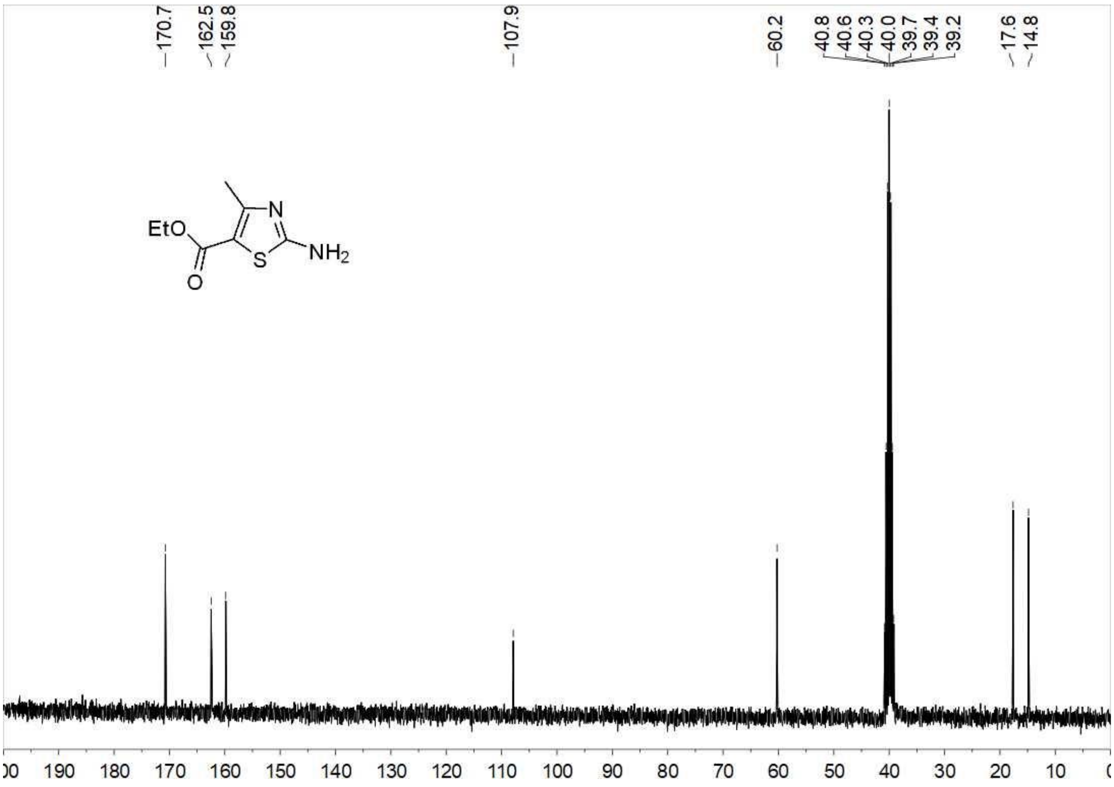

^{1}H NMR of methyl 2-amino-4-methylthiazole-5-carboxylate (3b)

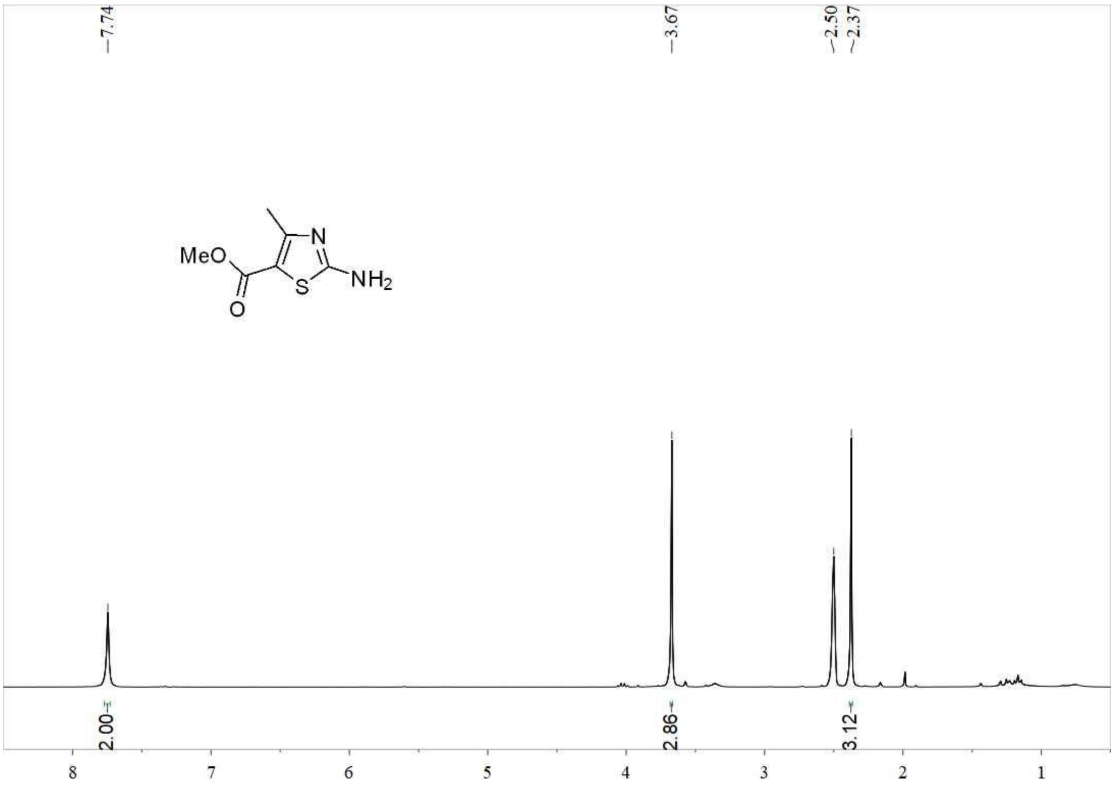

^{13}C NMR of methyl 2-amino-4-methylthiazole-5-carboxylate (3b)

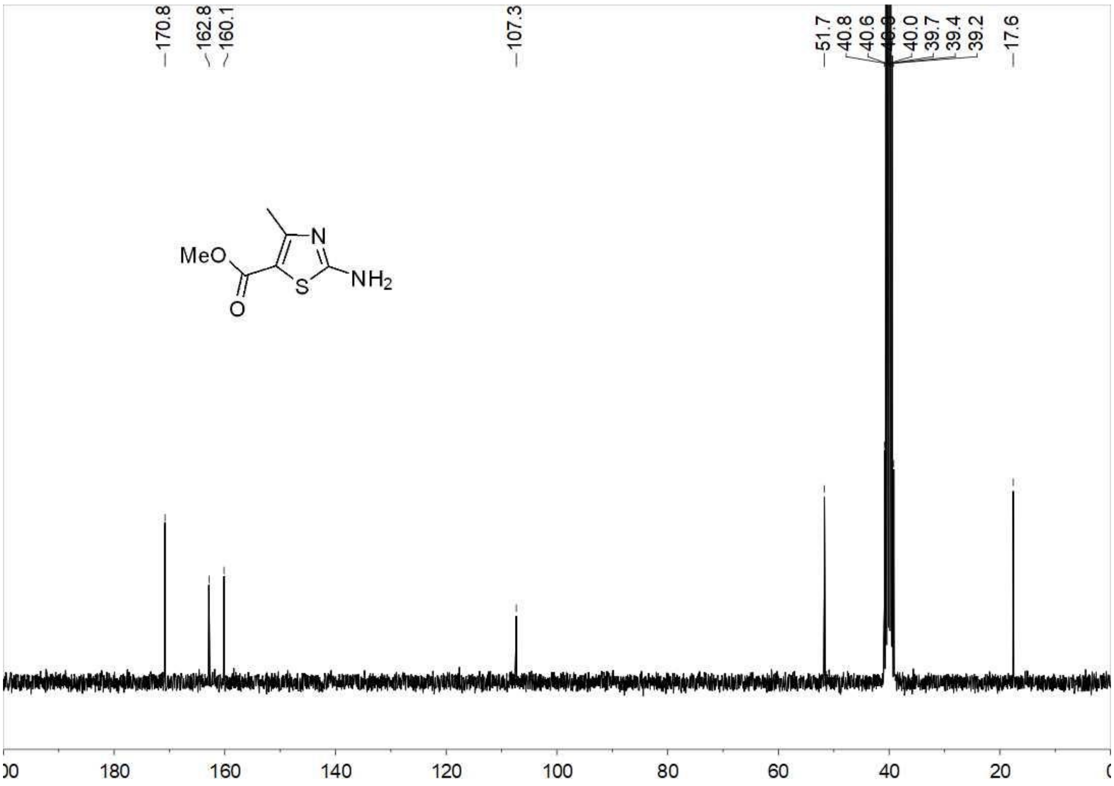

^{1}H NMR of *tert*-butyl 2-amino-4-methylthiazole-5-carboxylate (3c)

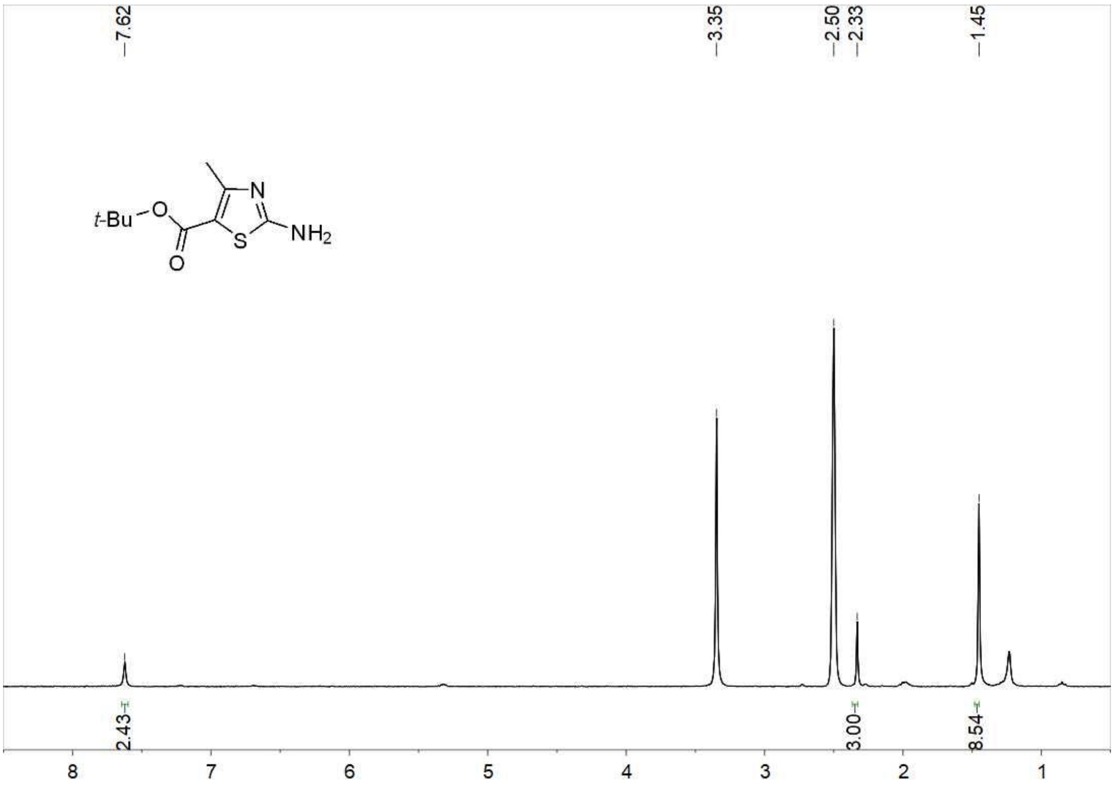

^{13}C NMR of *tert*-butyl 2-amino-4-methylthiazole-5-carboxylate (3c)

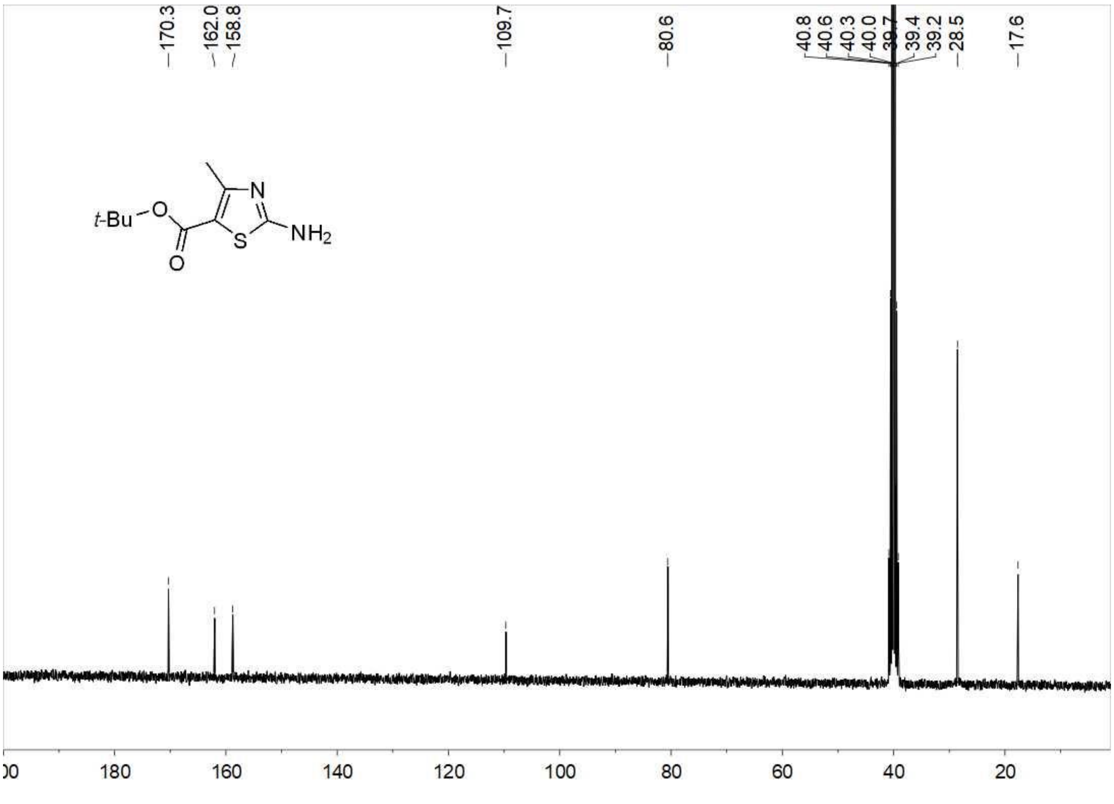

^{1}H NMR of pentyl 2-amino-4-methylthiazole-5-carboxylate (3d)

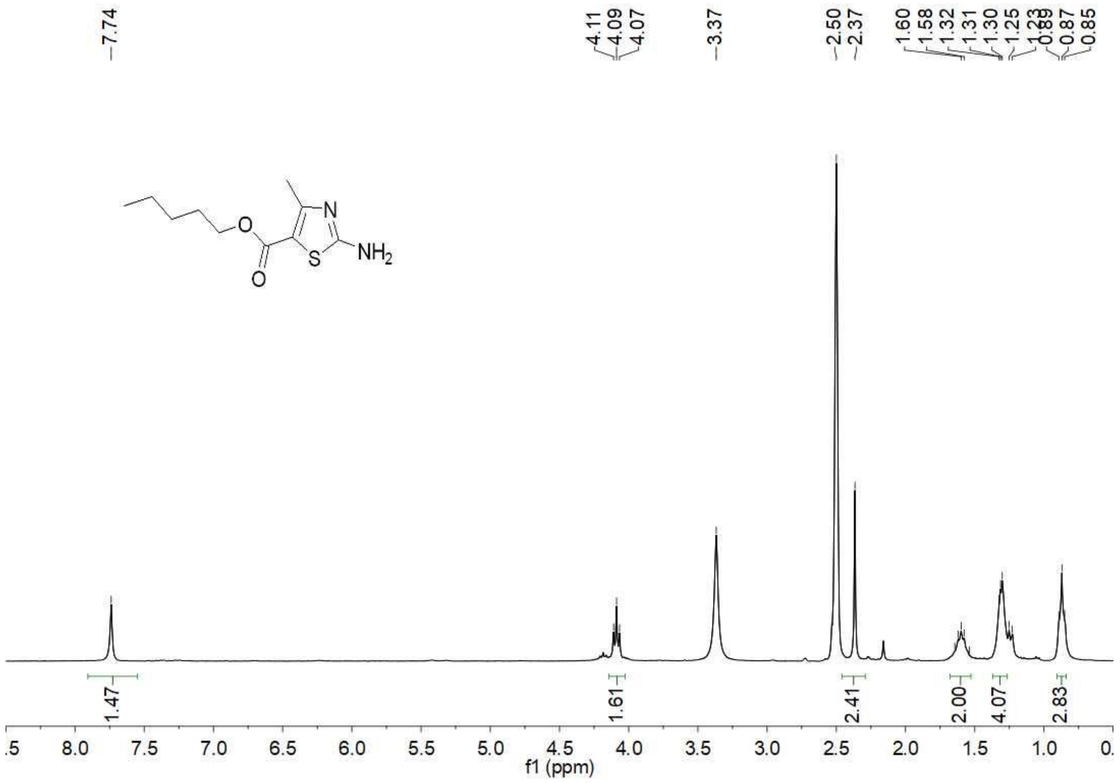

^{13}C NMR of pentyl 2-amino-4-methylthiazole-5-carboxylate (3d)

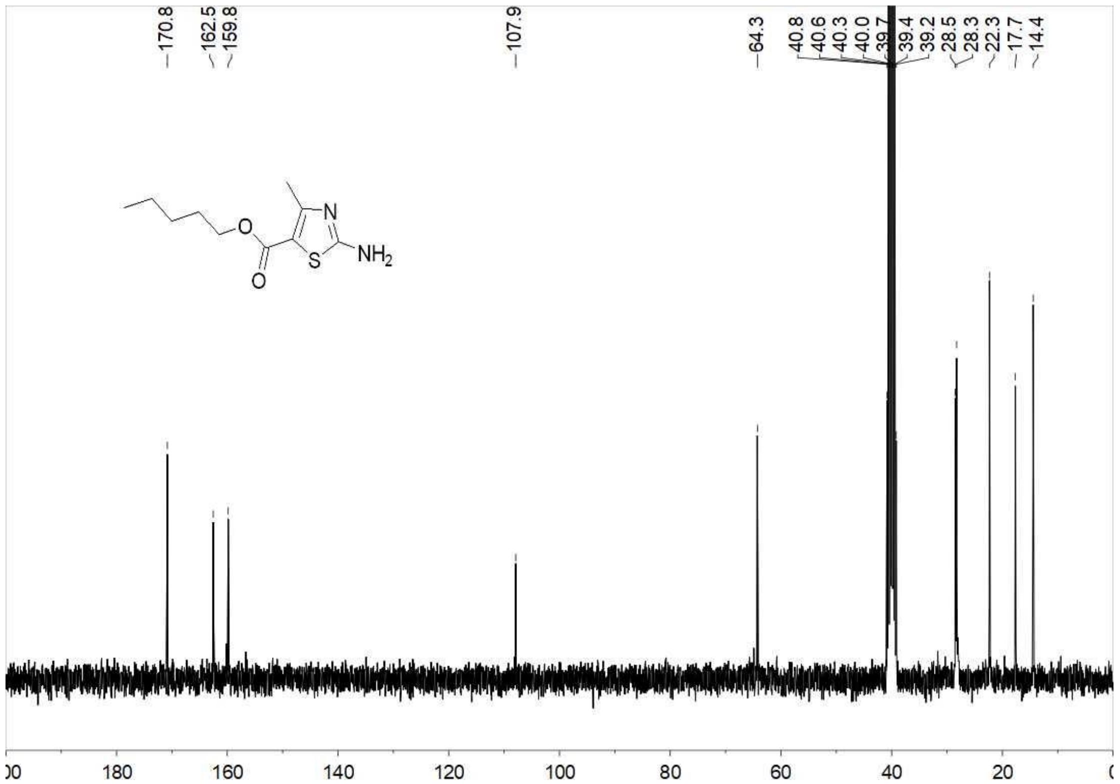

^{1}H NMR of allyl 2-amino-4-methylthiazole-5-carboxylate (3e)

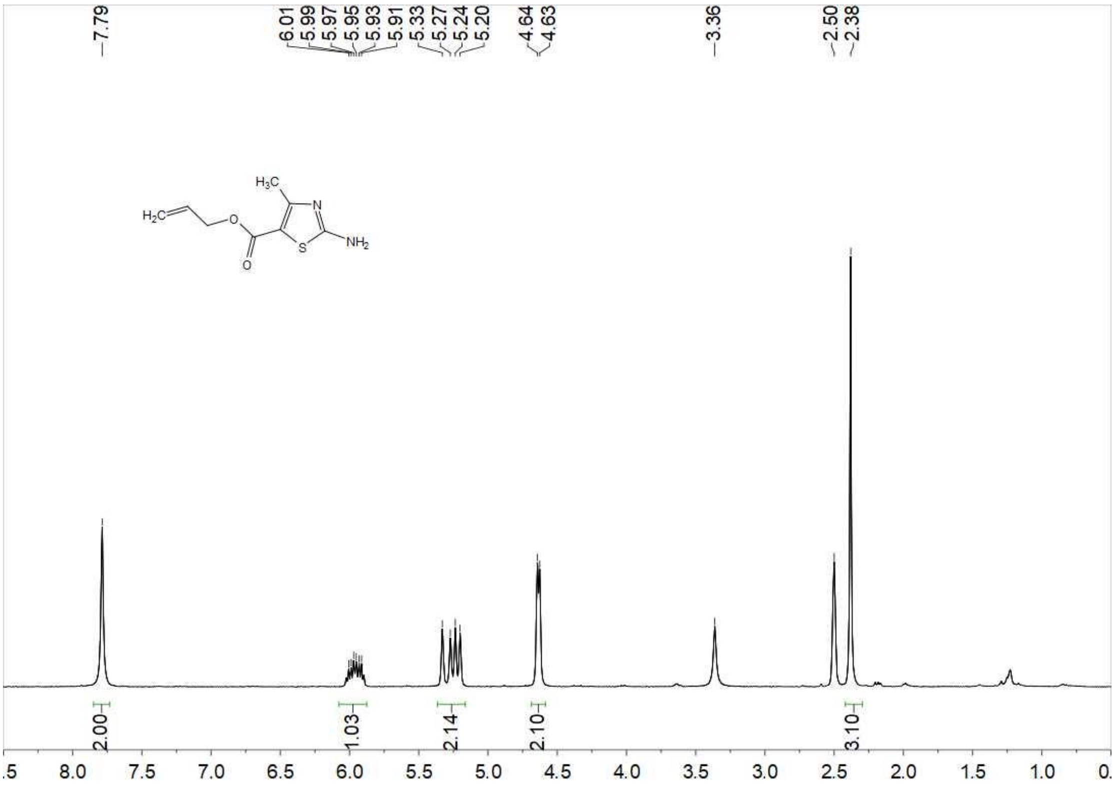

^{13}C NMR of allyl 2-amino-4-methylthiazole-5-carboxylate (3e)

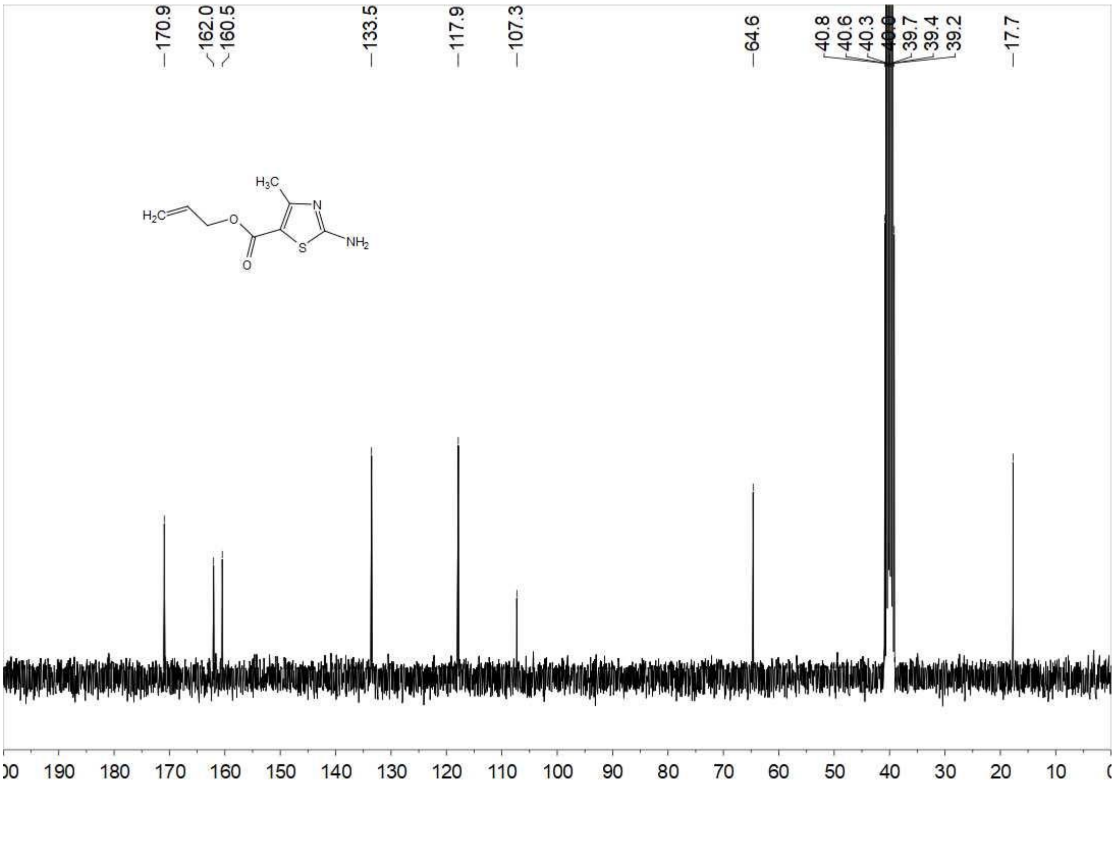

^{1}H NMR of benzyl 2-amino-4-methylthiazole-5-carboxylate (3f)

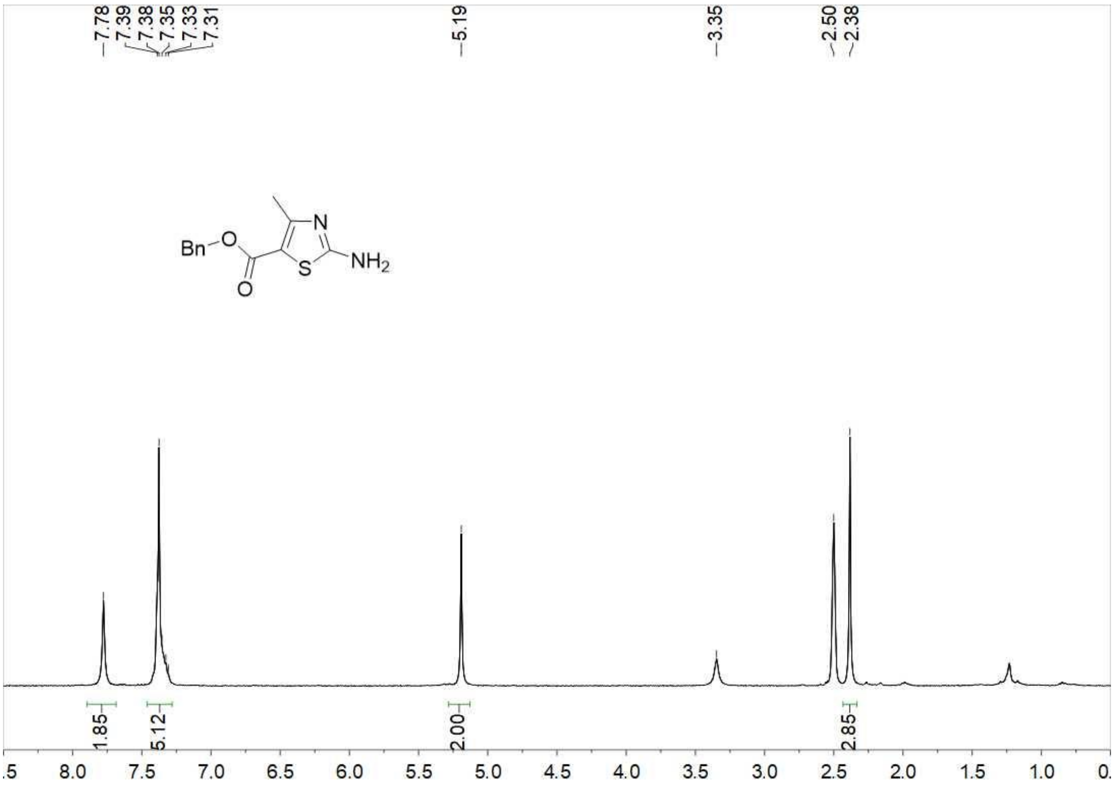

^{13}C NMR of benzyl 2-amino-4-methylthiazole-5-carboxylate (3f)

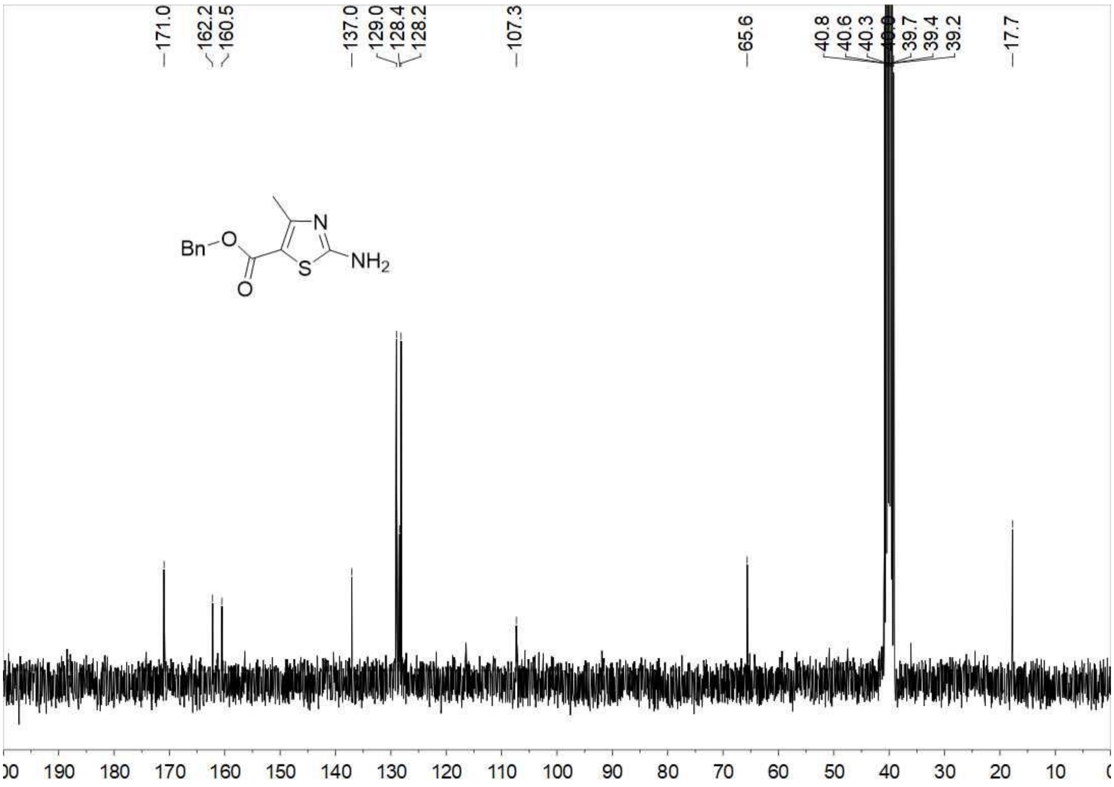

HRMS of benzyl 2-amino-4-methylthiazole-5-carboxylate (3f)

Peking University Mass Spectrometry Sample Analysis Report

Analysis Info

Analysis Name	FTMS-22050052_Pos_20220511_000001.d	Acquisition Date	5/11/2022 2:43:57 PM
Sample	0509-01	Instrument	Bruker Solarix XR FTMS
Comment		Operator	Peking University

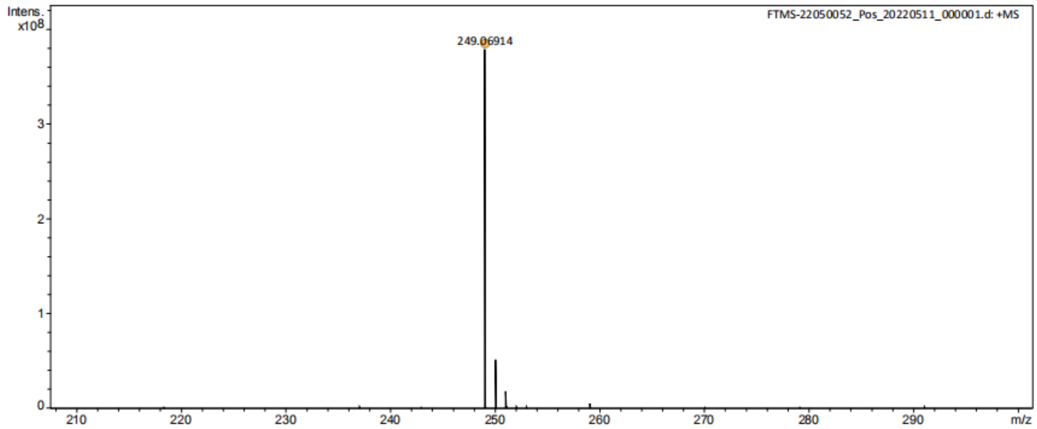

Meas. m/z	#	Ion Formula	Score	m/z	err [ppm]	Mean err [ppm]	mSigma	rdb	e⁻ Conf	N-Rule
249.069135	1	C12H13N2O2S	100.00	249.069225	0.4	-0.7	3.1	10.0	even	ok

Bruker Compass DataAnalysis 5.0 printed: 5/11/2022 2:44:55 PM Page 1 of 1

^{1}H NMR of ethyl 2-amino-4-ethylthiazole-5-carboxylate (3g)

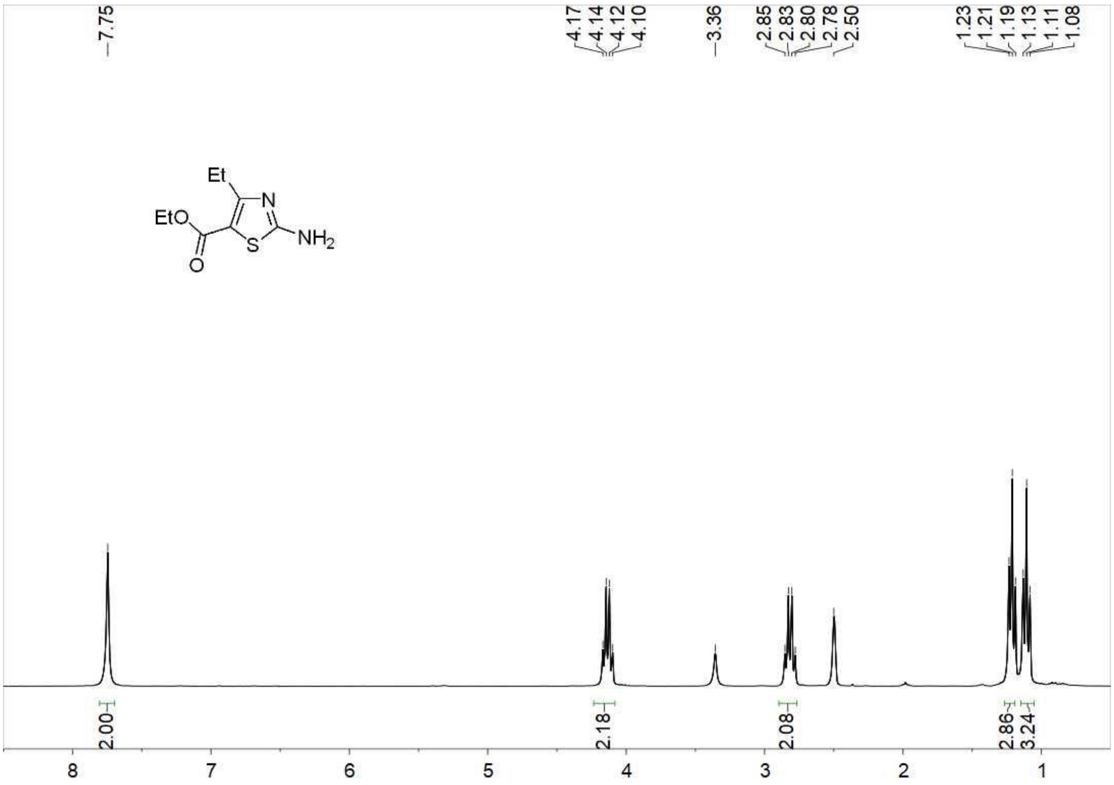

^{13}C NMR of ethyl 2-amino-4-ethylthiazole-5-carboxylate (3g)

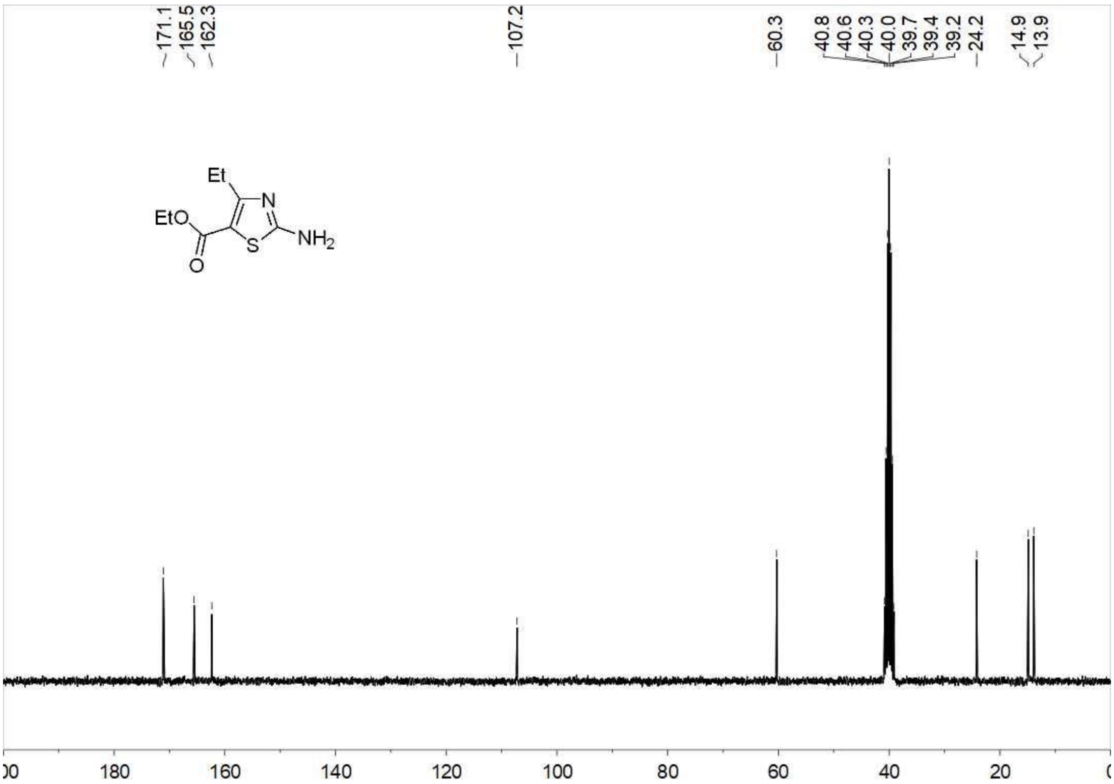

HRMS of ethyl 2-amino-4-ethylthiazole-5-carboxylate (3g)

Peking University Mass Spectrometry Sample Analysis Report

Analysis Info

Analysis Name	FTMS-22050052_Pos_20220511_000002.d	Acquisition Date	5/11/2022 2:46:49 PM
Sample	0509-02	Instrument	Bruker Solarix XR FTMS
Comment		Operator	Peking University

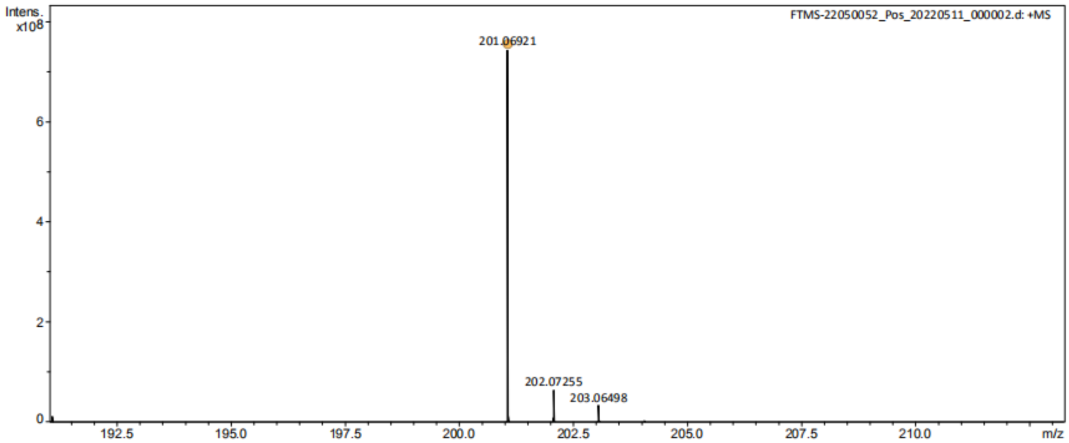

Meas. m/z	#	Ion Formula	Score	m/z	err [ppm]	Mean err [ppm]	mSigma	rdb	e⁻ Conf	N-Rule
201.069209	1	C8H13N2O2S	100.00	201.069225	0.1	-0.1	4.4	6.0	even	ok

Bruker Compass DataAnalysis 5.0 printed: 5/11/2022 2:47:39 PM Page 1 of 1

^{1}H NMR of ethyl 2-amino-4-propylthiazole-5-carboxylate (3h)

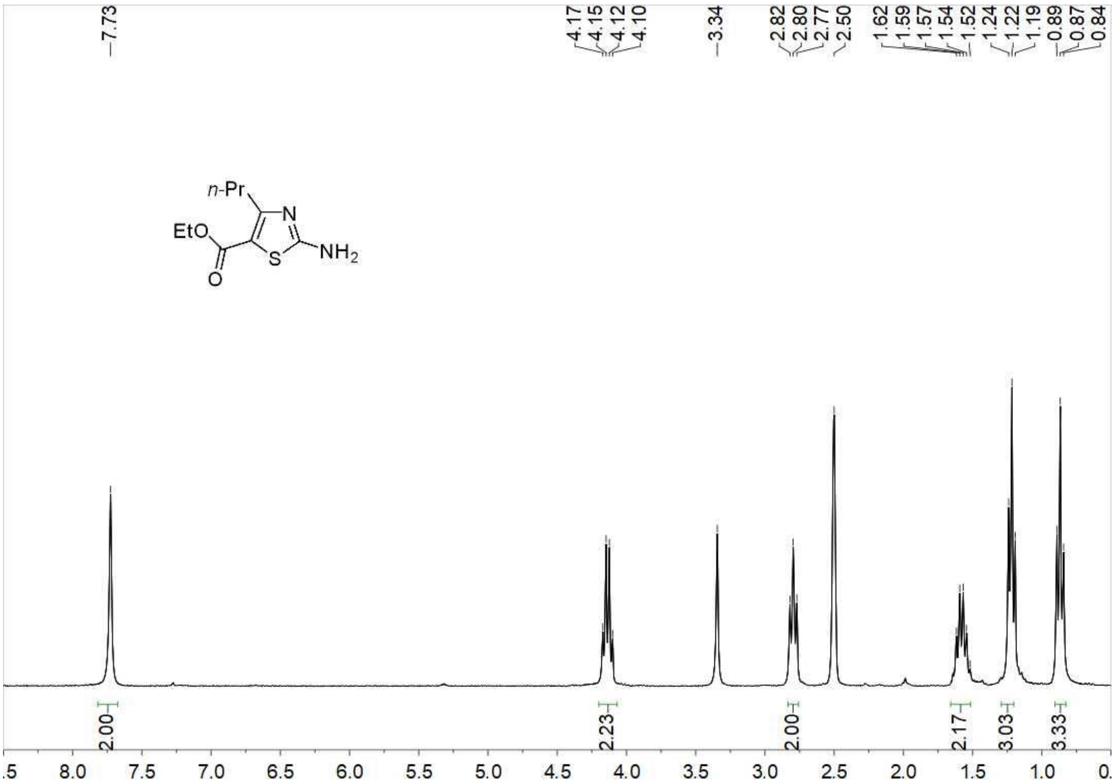

^{13}C NMR of ethyl 2-amino-4-propylthiazole-5-carboxylate (3h)

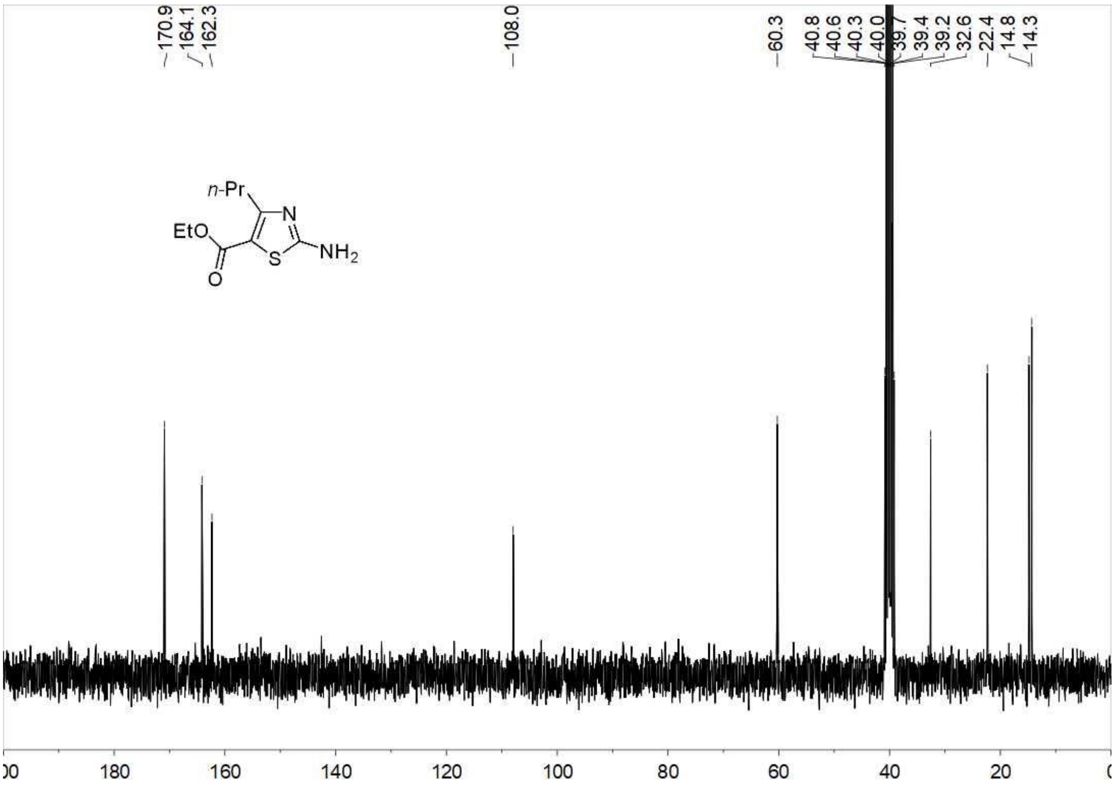

^{1}H NMR of ethyl 2-amino-4-isopropylthiazole-5-carboxylate (3i)

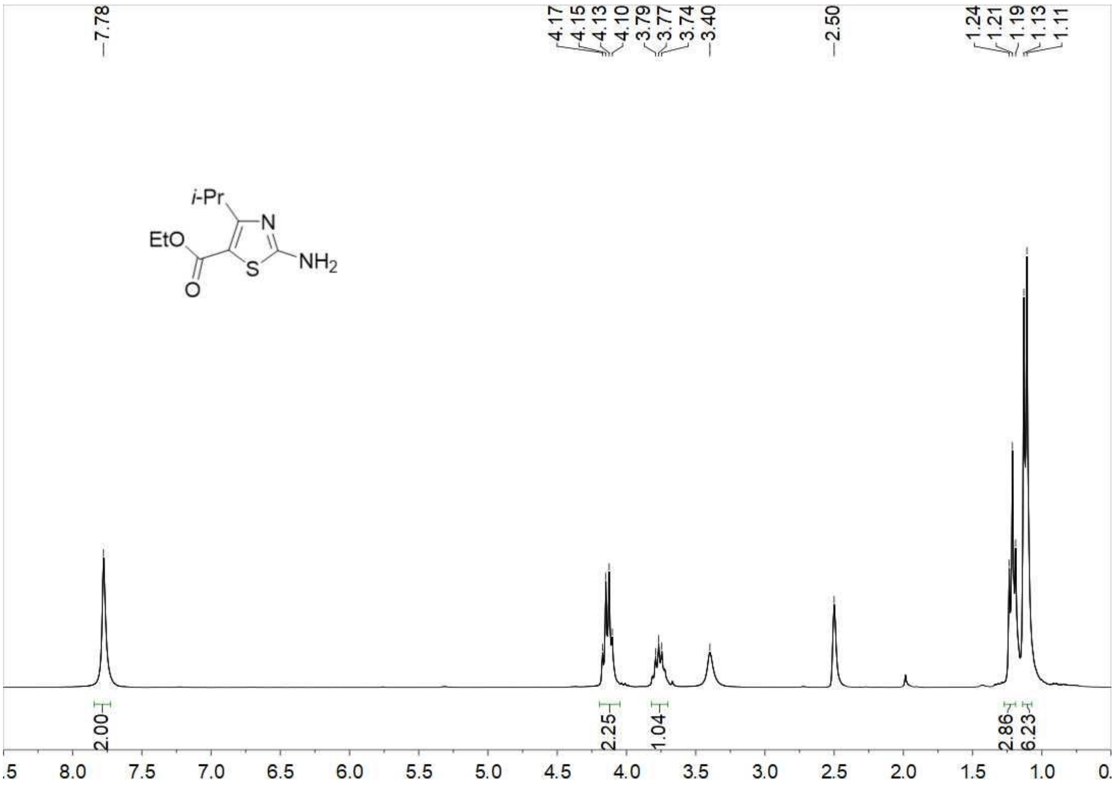

^{13}C NMR of ethyl 2-amino-4-isopropylthiazole-5-carboxylate (3i)

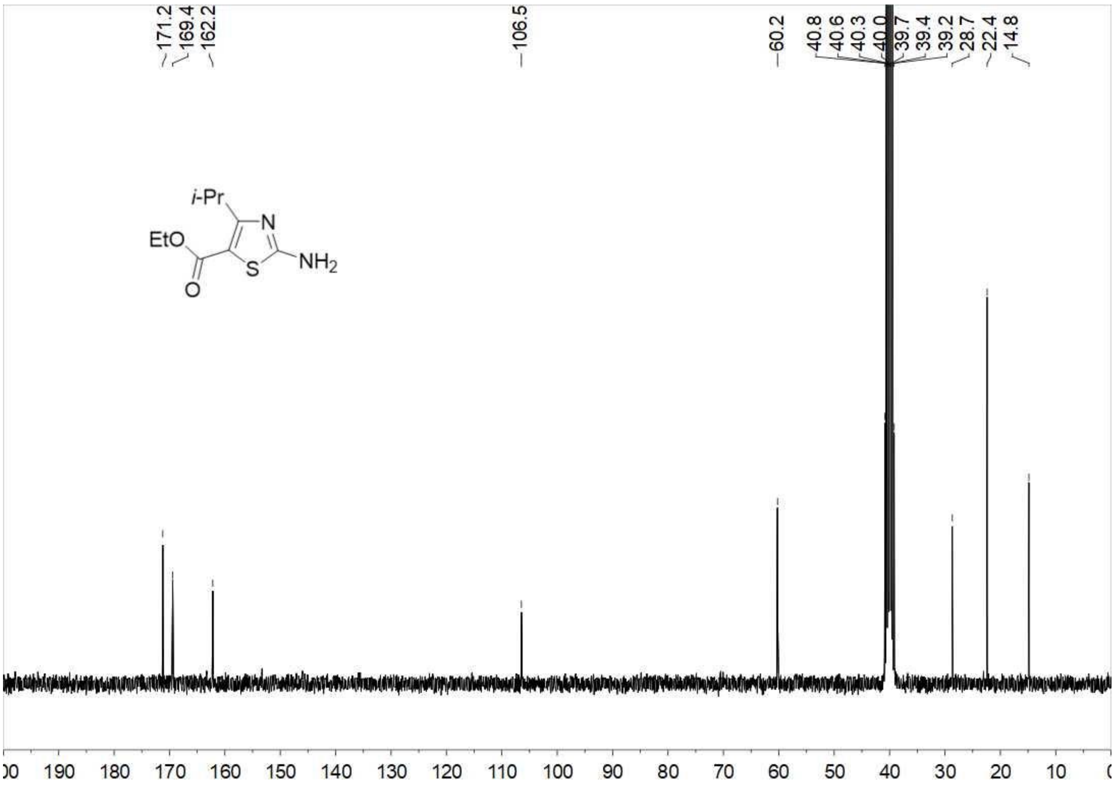

^{1}H NMR of ethyl 2-amino-4-butylthiazole-5-carboxylate (3j)

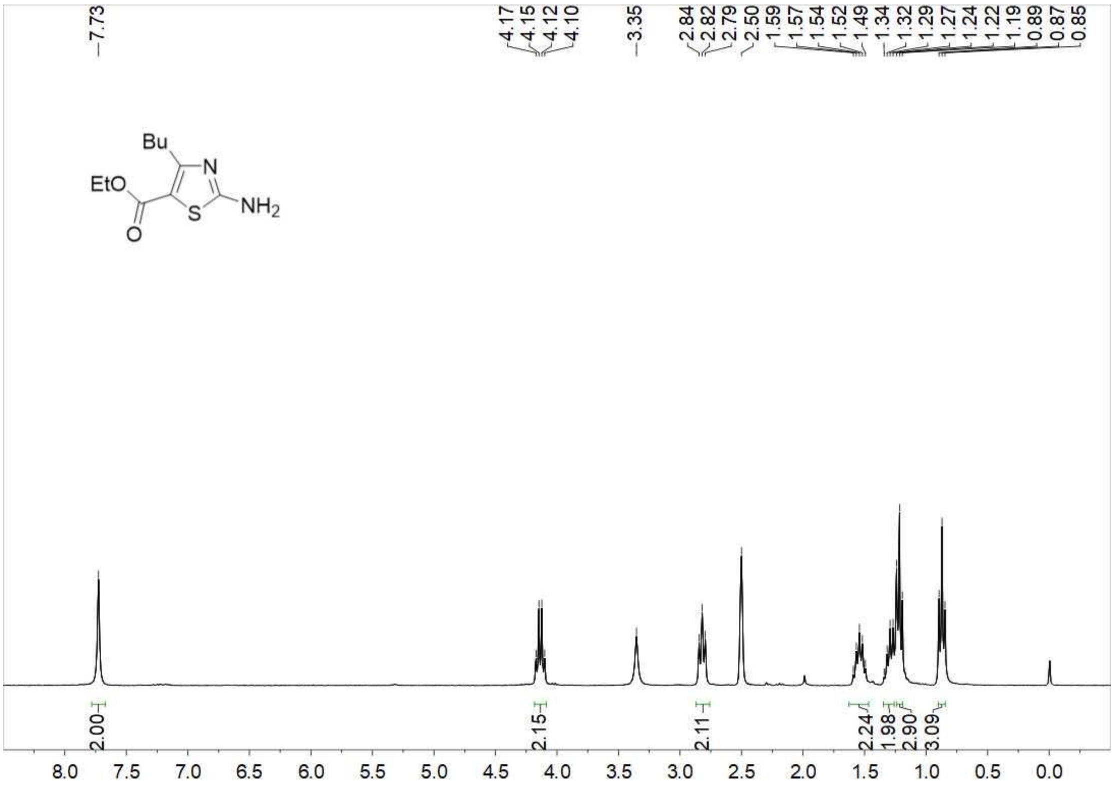

^{13}C NMR of ethyl 2-amino-4-butylthiazole-5-carboxylate (3j)

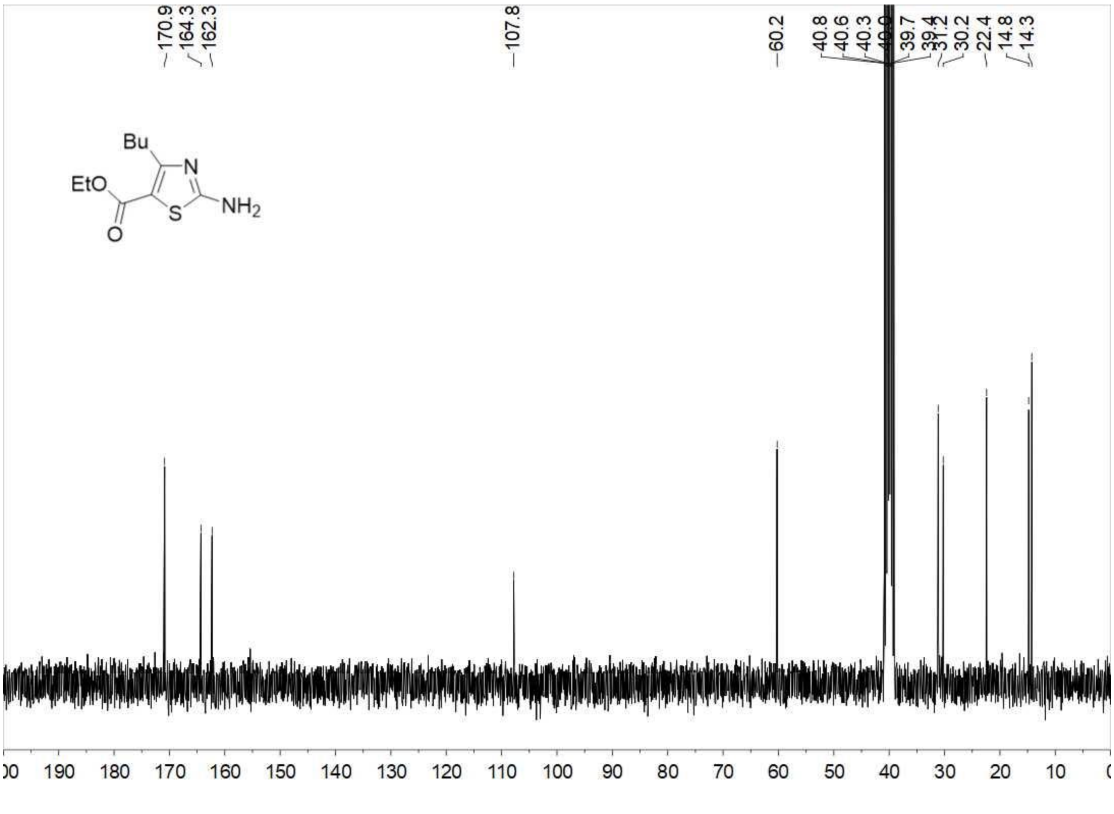

HRMS of ethyl 2-amino-4-butylthiazole-5-carboxylate (3j)

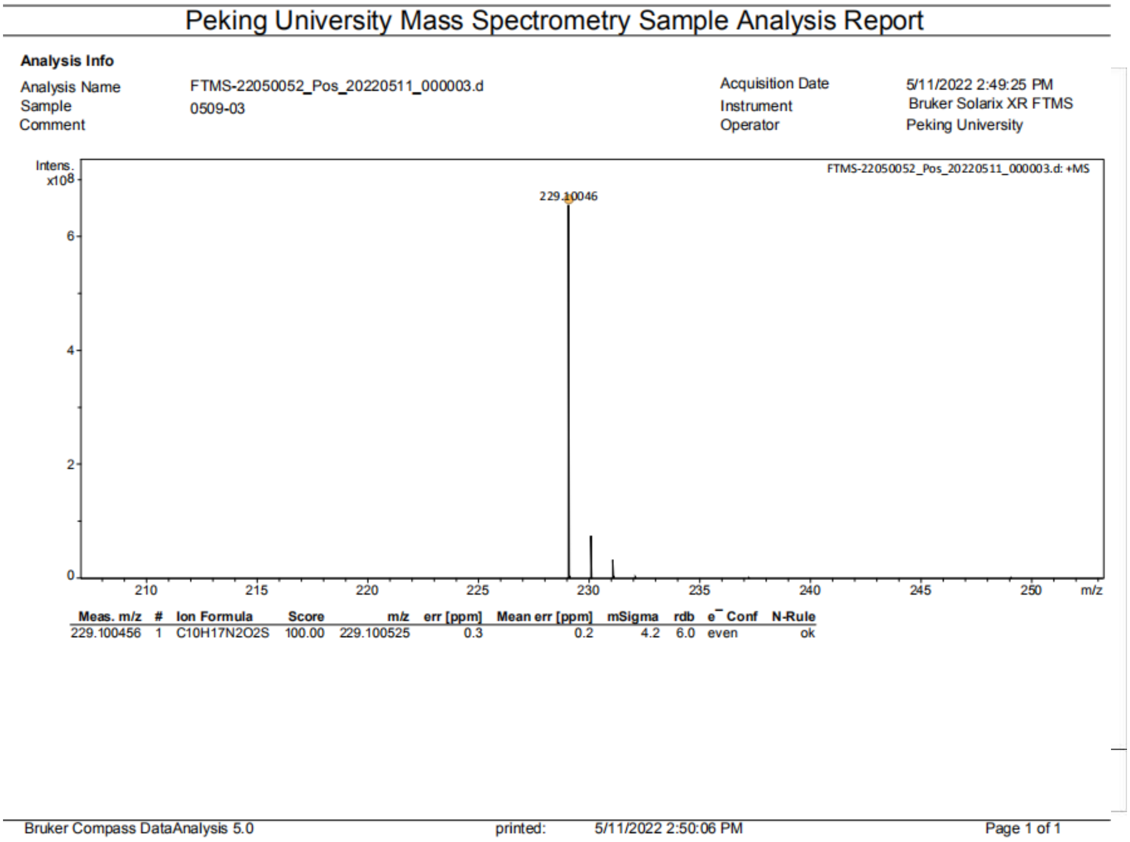

Peking University Mass Spectrometry Sample Analysis Report

Analysis Info

Analysis Name	FTMS-22050052_Pos_20220511_000003.d	Acquisition Date	5/11/2022 2:49:25 PM
Sample	0509-03	Instrument	Bruker Solarix XR FTMS
Comment		Operator	Peking University

Meas. m/z	#	Ion Formula	Score	m/z	err [ppm]	Mean err [ppm]	mSigma	rdb	e⁻ Conf	N-Rule
229.100456	1	C10H17N2O2S	100.00	229.100525	0.3	0.2	4.2	6.0	even	ok

Bruker Compass DataAnalysis 5.0 printed: 5/11/2022 2:50:06 PM Page 1 of 1

^{1}H NMR of ethyl 2-amino-4-(*tert*-butyl) thiazole-5-carboxylate (3k)

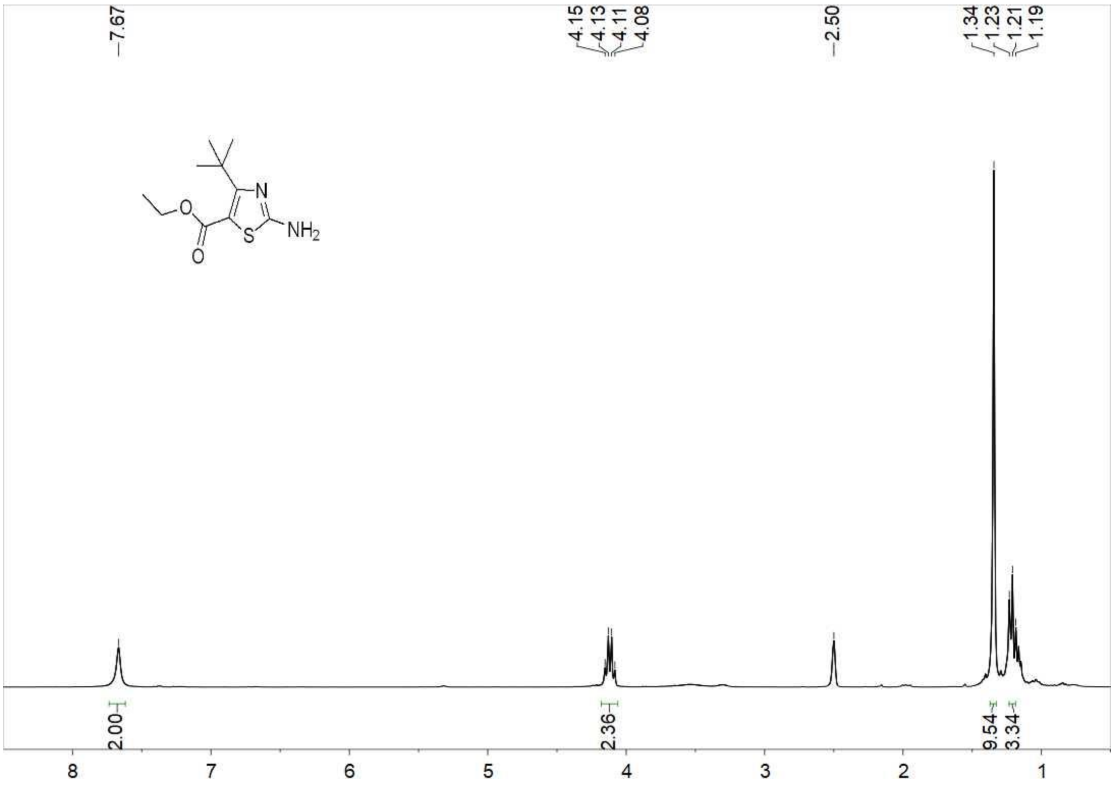

^{13}C NMR of ethyl 2-amino-4-(*tert*-butyl) thiazole-5-carboxylate (3k)

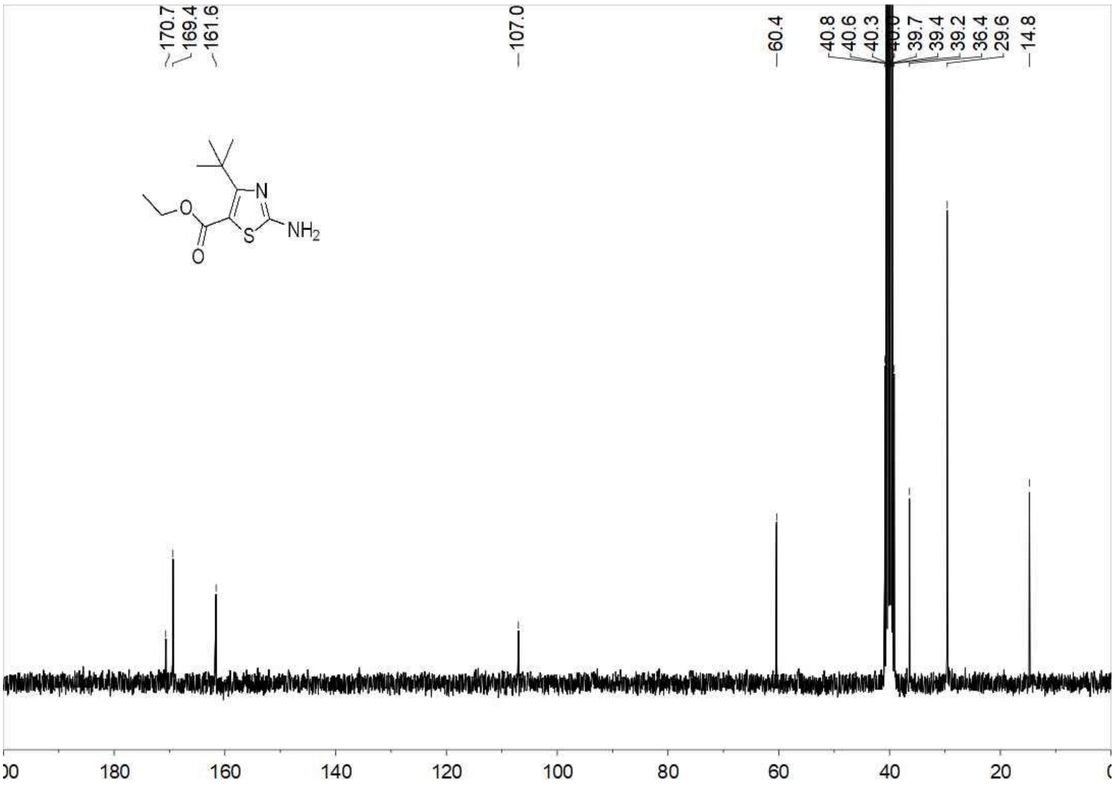

HRMS of ethyl 2-amino-4-(*tert*-butyl) thiazole-5-carboxylate (3k)

Peking University Mass Spectrometry Sample Analysis Report

Analysis Info

Analysis Name	FTMS-22050052_Pos_20220511_000004.d	Acquisition Date	5/11/2022 2:51:57 PM
Sample	0509-04	Instrument	Bruker Solarix XR FTMS
Comment		Operator	Peking University

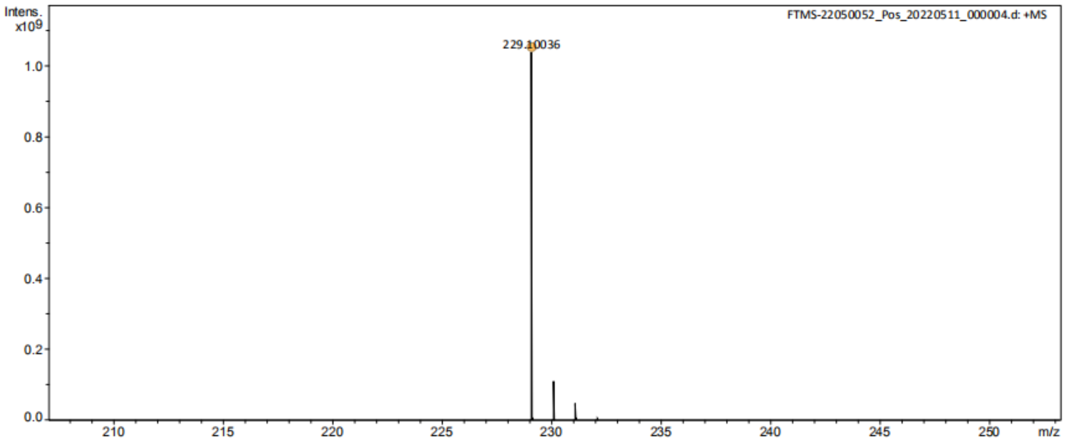

Meas. m/z	#	Ion Formula	Score	m/z	err [ppm]	Mean err [ppm]	mSigma	rdb	e⁻ Conf	N-Rule
229.100359	1	C10H17N2O2S	100.00	229.100525	0.7	0.4	6.3	6.0	even	ok

Bruker Compass DataAnalysis 5.0 printed: 5/11/2022 2:52:19 PM Page 1 of 1

^{1}H NMR of ethyl 2-amino-4-cyclohexylthiazole-5-carboxylate (3l)

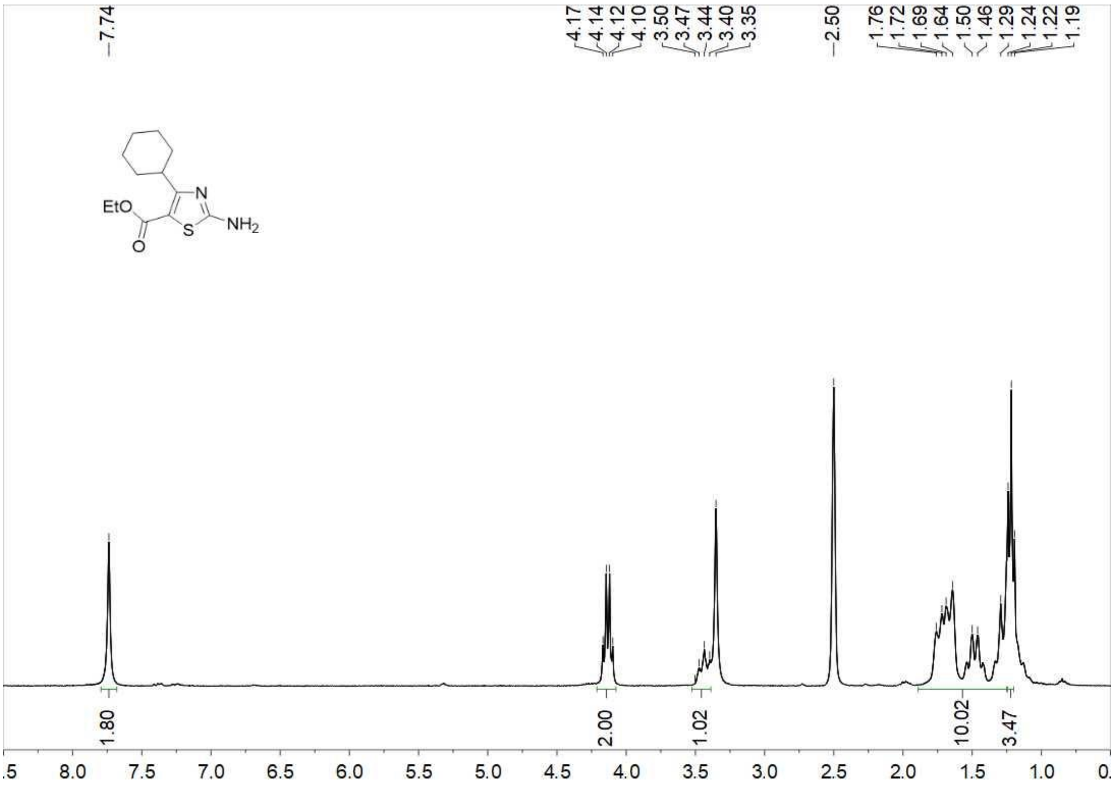

^{13}C NMR of ethyl 2-amino-4-cyclohexylthiazole-5-carboxylate (3l)

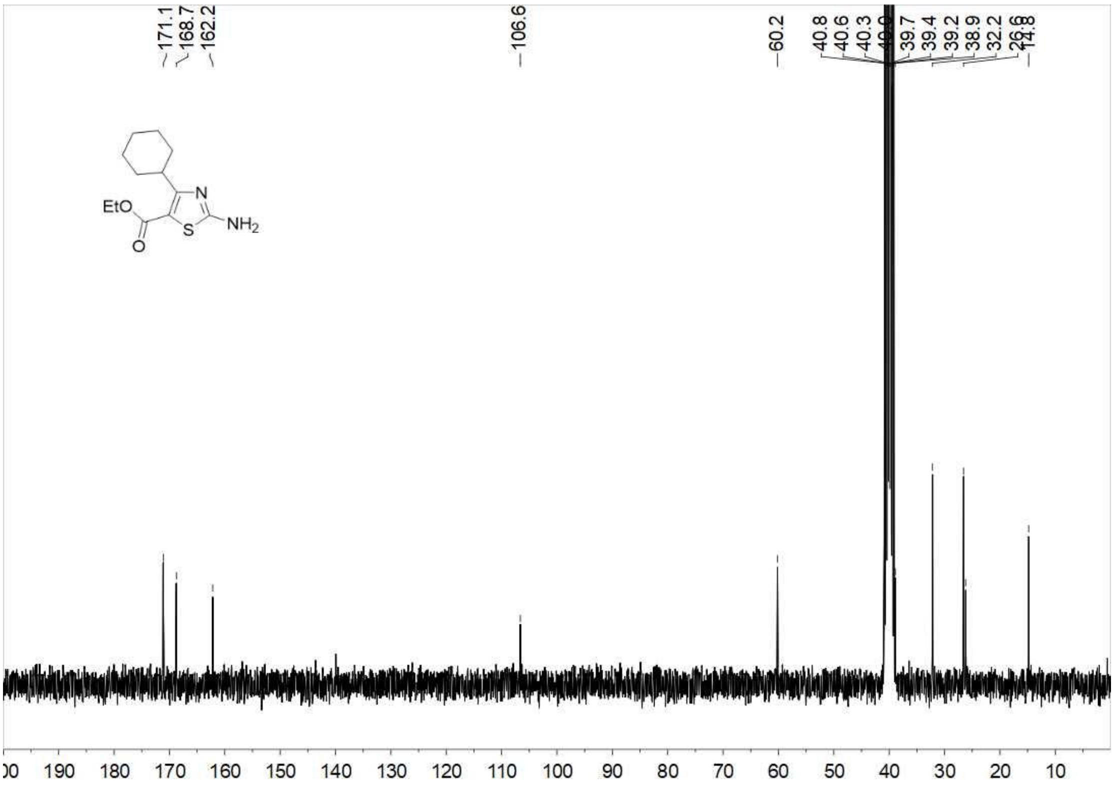

HRMS of ethyl 2-amino-4-cyclohexylthiazole-5-carboxylate (3l)

Peking University Mass Spectrometry Sample Analysis Report

Analysis Info

Analysis Name	FTMS-22050052_Pos_20220511_000005.d	Acquisition Date	5/11/2022 2:54:14 PM
Sample	0509-05	Instrument	Bruker Solarix XR FTMS
Comment		Operator	Peking University

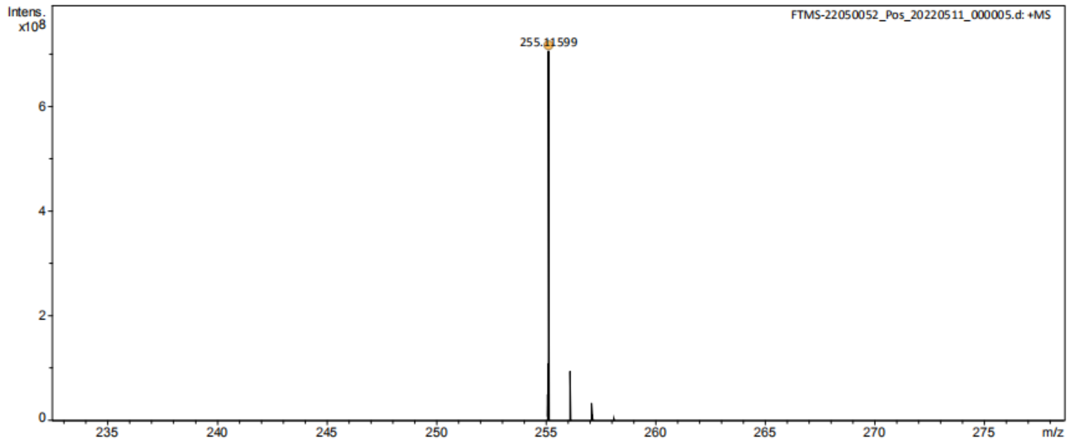

Meas. m/z	#	Ion Formula	Score	m/z	err [ppm]	Mean err [ppm]	mSigma	rdb	e⁻ Conf	N-Rule
255.115993	1	C12H19N2O2S	100.00	255.116175	0.7	-0.9	2.8	7.0	even	ok

Bruker Compass DataAnalysis 5.0 printed: 5/11/2022 2:54:45 PM Page 1 of 1

^{1}H NMR of ethyl 2-amino-4-phenylthiazole-5-carboxylate (3m)

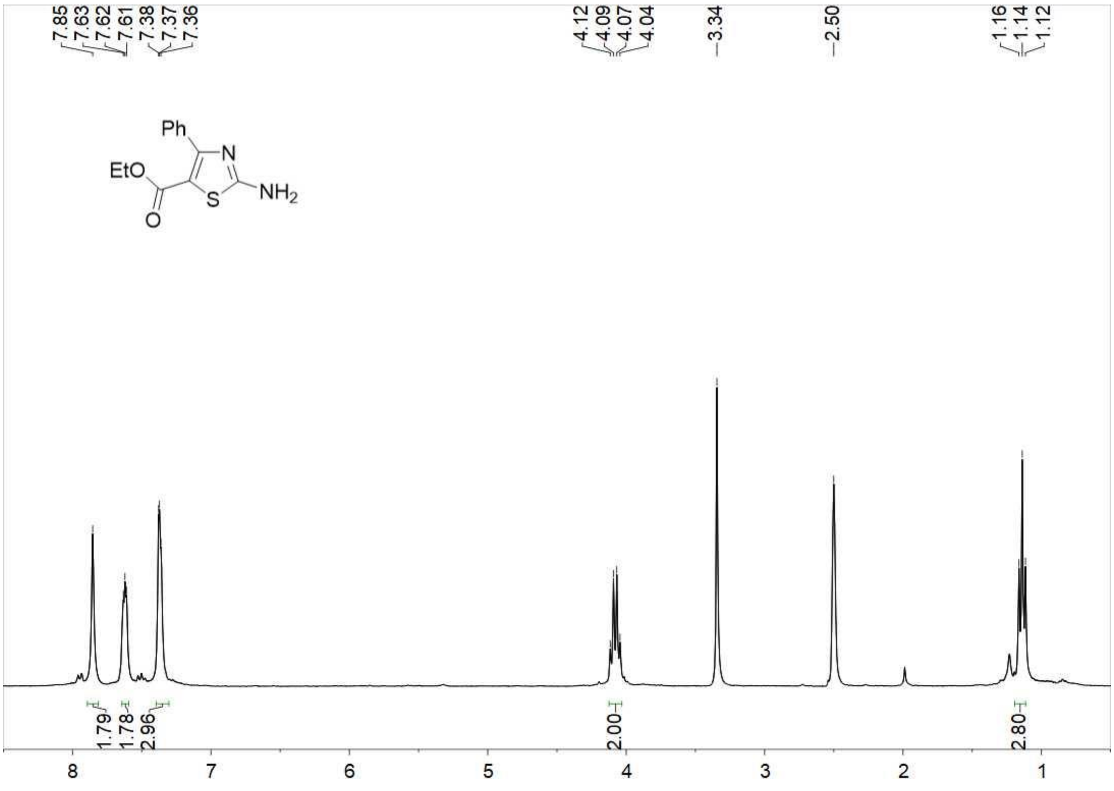

^{13}C NMR of ethyl 2-amino-4-phenylthiazole-5-carboxylate (3m)

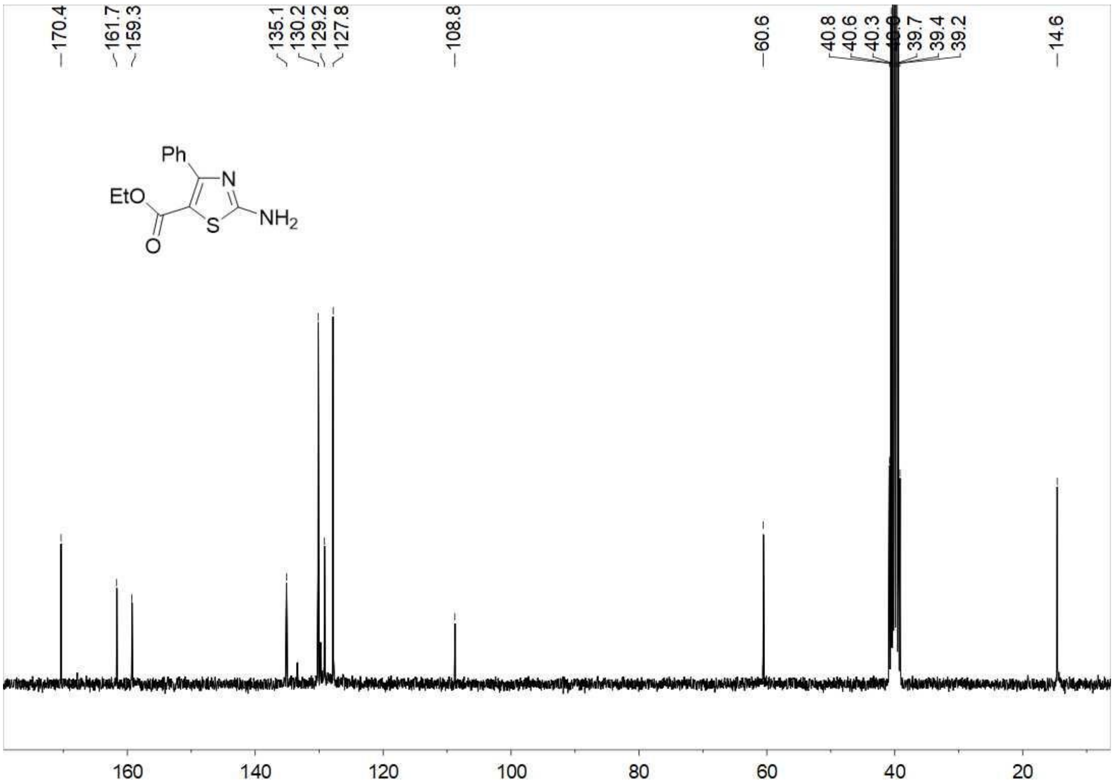

^{1}H NMR of ethyl 2-amino-4-(*p*-tolyl) thiazole-5-carboxylate (3n)

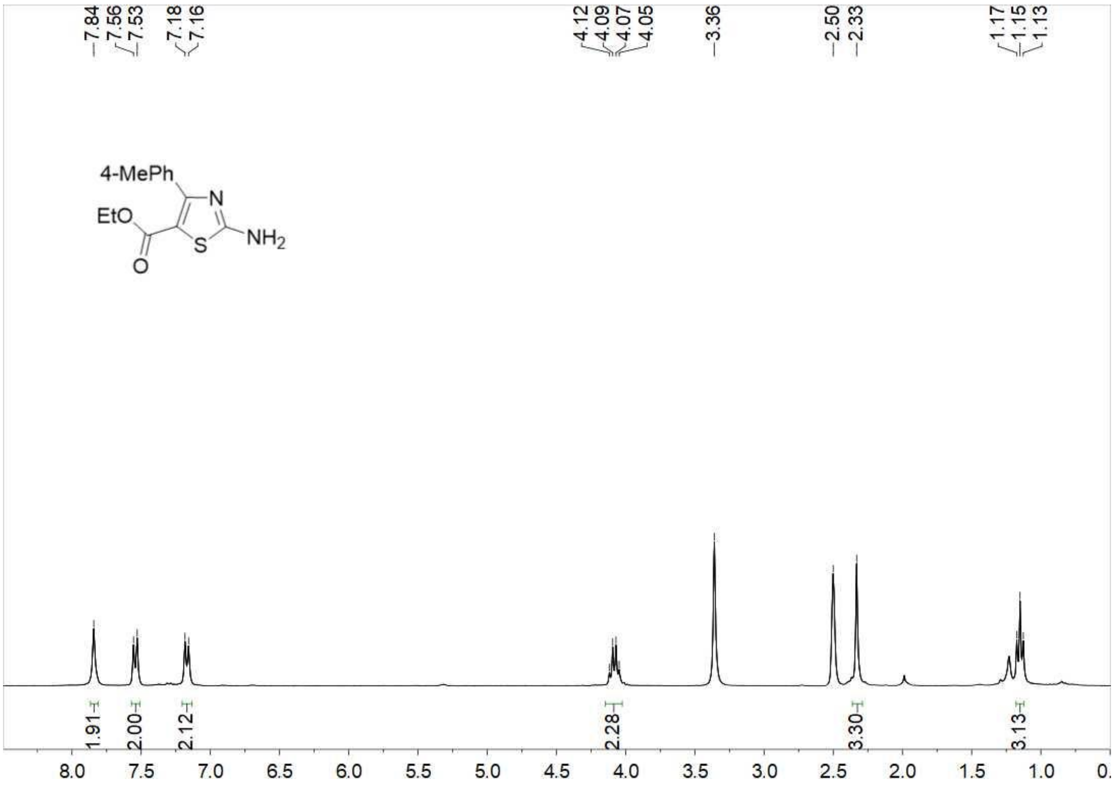

^{13}C NMR of ethyl 2-amino-4-(*p*-tolyl) thiazole-5-carboxylate (3n)

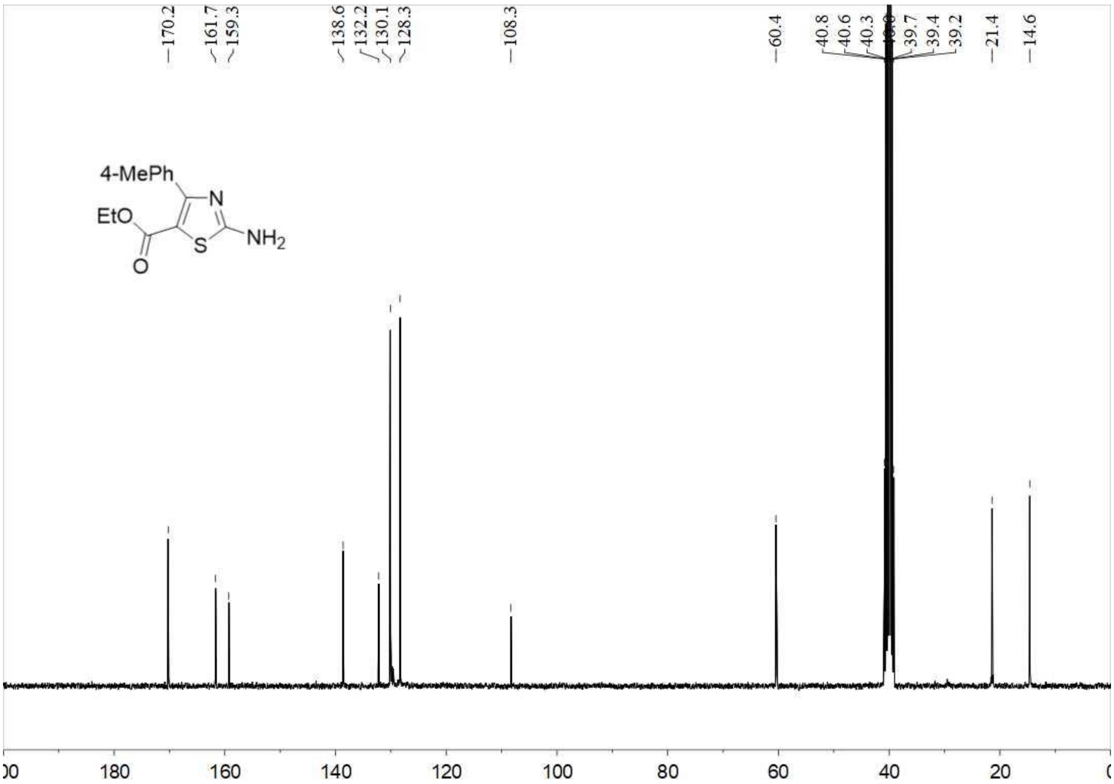

HRMS of ethyl 2-amino-4-(*p*-tolyl) thiazole-5-carboxylate (3n)

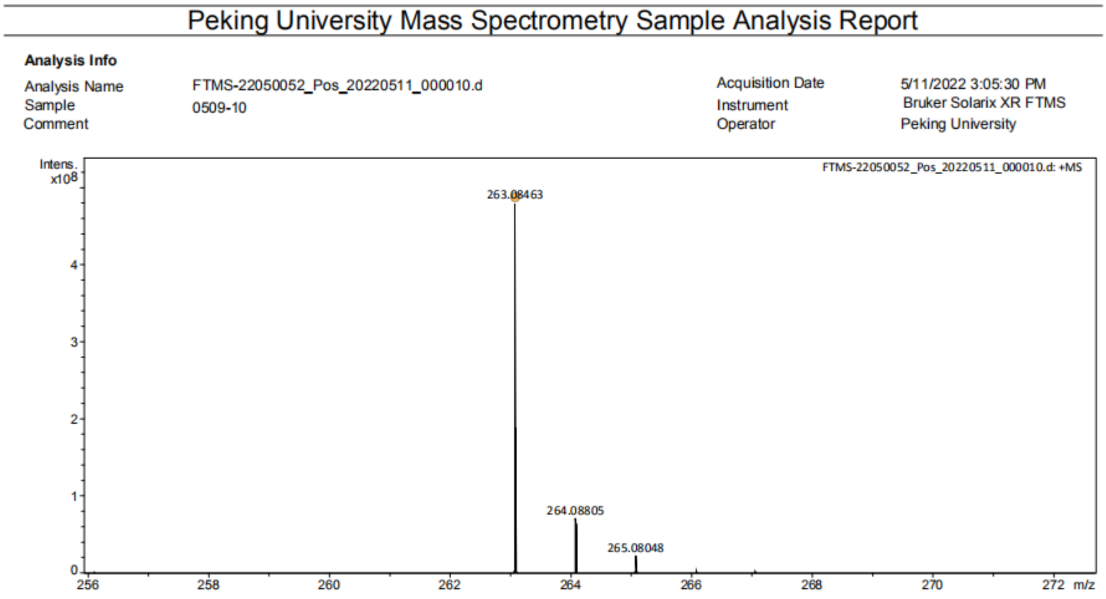

Meas. m/z	#	Ion Formula	Score	m/z	err [ppm]	Mean err [ppm]	mSigma	rdb	e⁻ Conf	N-Rule
263.084635	1	C13H15N2O2S	100.00	263.084875	0.9	-0.5	3.6	10.0	even	ok

Bruker Compass DataAnalysis 5.0 printed: 5/11/2022 3:06:00 PM Page 1 of 1

^{1}H NMR of ethyl 2-amino-4-(4-methoxyphenyl) thiazole-5-carboxylate (3o)

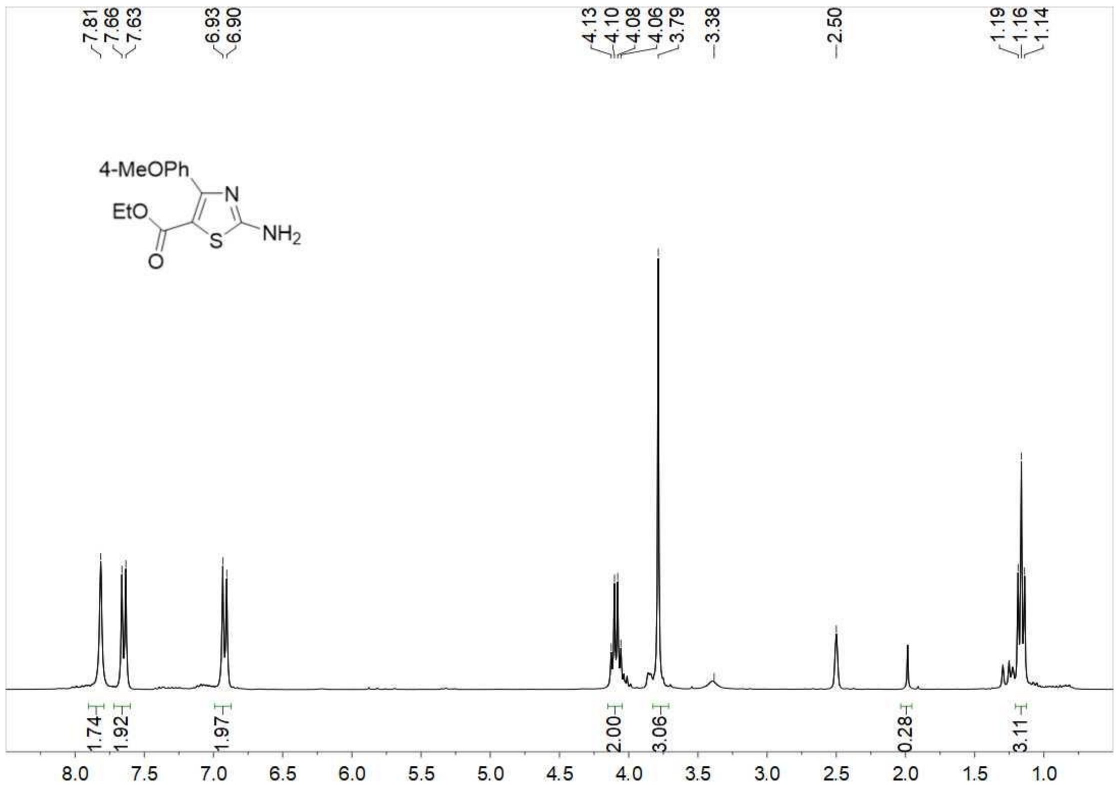

^{13}C NMR of ethyl 2-amino-4-(4-methoxyphenyl) thiazole-5-carboxylate (3o)

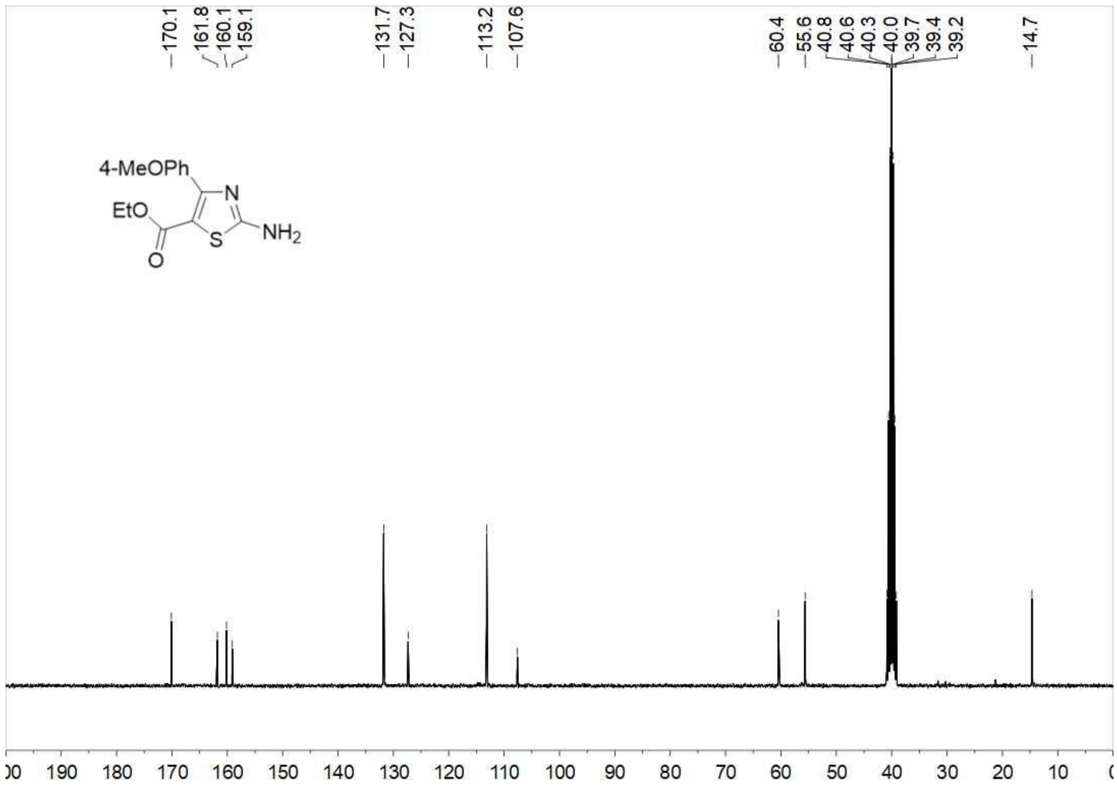

HRMS of ethyl 2-amino-4-(4-methoxyphenyl) thiazole-5-carboxylate (3o)

Peking University Mass Spectrometry Sample Analysis Report

Analysis Info

Analysis Name	FTMS-22050052_Pos_20220511_000007.d	Acquisition Date	5/11/2022 2:59:08 PM
Sample	0509-07	Instrument	Bruker Solarix XR FTMS
Comment		Operator	Peking University

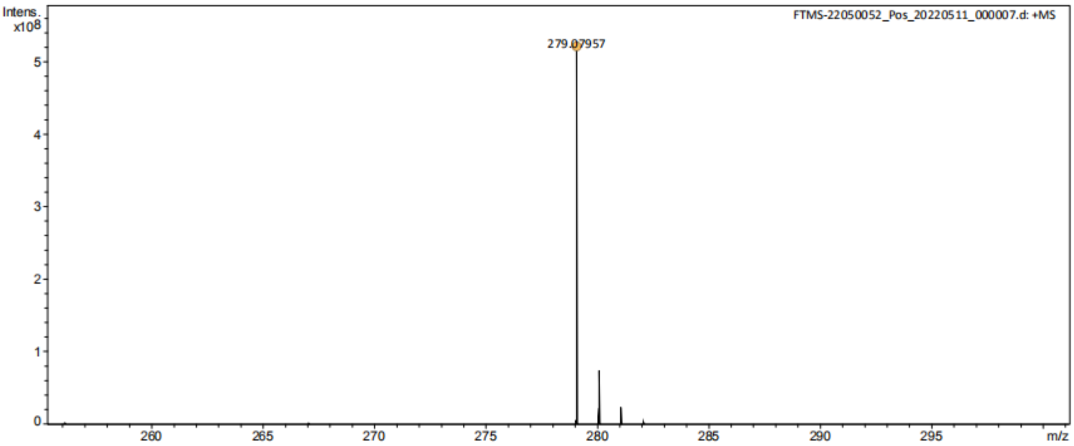

Meas. m/z	#	Ion Formula	Score	m/z	err [ppm]	Mean err [ppm]	mSigma	rdb	e⁻ Conf	N-Rule
279.079570	1	C13H15N2O3S	100.00	279.079790	0.8	0.5	4.0	10.0	even	ok

Bruker Compass DataAnalysis 5.0 printed: 5/11/2022 2:59:37 PM Page 1 of 1

^{1}H NMR of ethyl 2-amino-4-(3-methoxyphenyl) thiazole-5-carboxylate (3p)

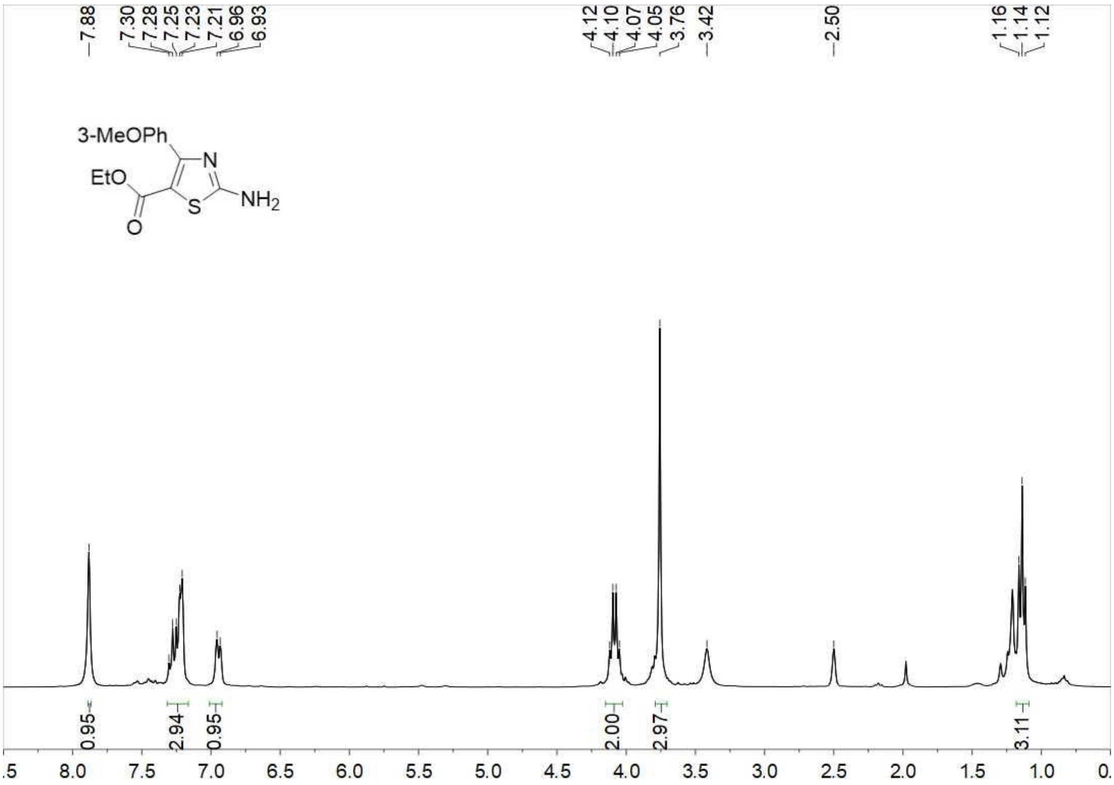

^{13}C NMR of ethyl 2-amino-4-(3-methoxyphenyl) thiazole-5-carboxylate (3p)

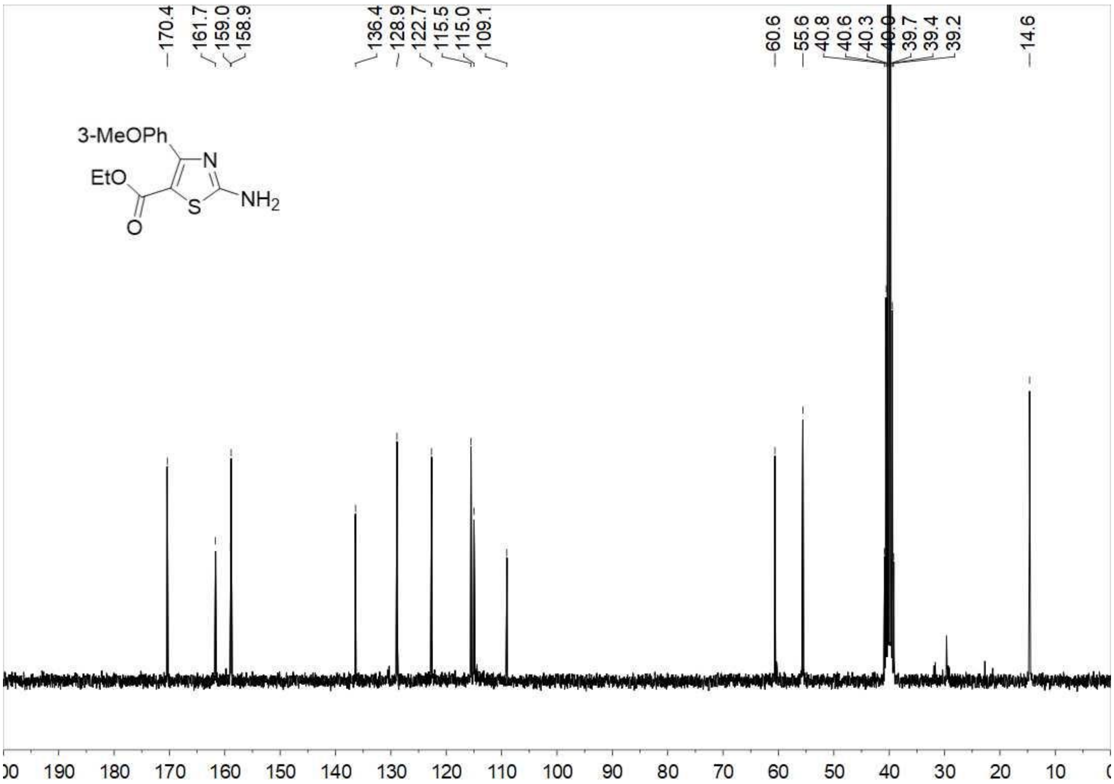

HRMS of ethyl 2-amino-4-(3-methoxyphenyl) thiazole-5-carboxylate (3p)

Peking University Mass Spectrometry Sample Analysis Report

Analysis Info

Analysis Name	FTMS-22050052_Pos_20220511_000008.d	Acquisition Date	5/11/2022 3:01:15 PM
Sample	0509-08	Instrument	Bruker Solarix XR FTMS
Comment		Operator	Peking University

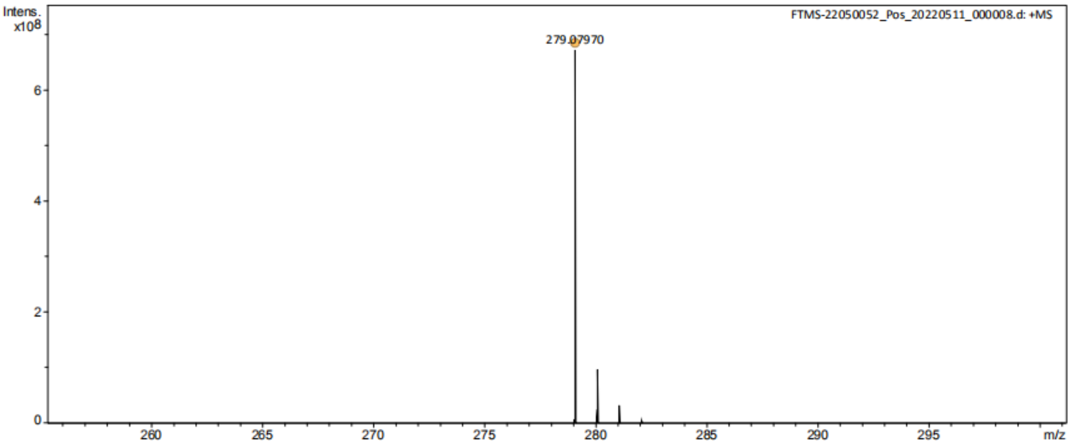

Meas. m/z	#	Ion Formula	Score	m/z	err [ppm]	Mean err [ppm]	mSigma	rdb	e⁻ Conf	N-Rule
279.079703	1	C13H15N2O3S	100.00	279.079790	0.3	-0.0	4.2	10.0	even	ok

Bruker Compass DataAnalysis 5.0 printed: 5/11/2022 3:01:34 PM Page 1 of 1

^{1}H NMR of ethyl 2-amino-4-(2-methoxyphenyl) thiazole-5-carboxylate (3q)

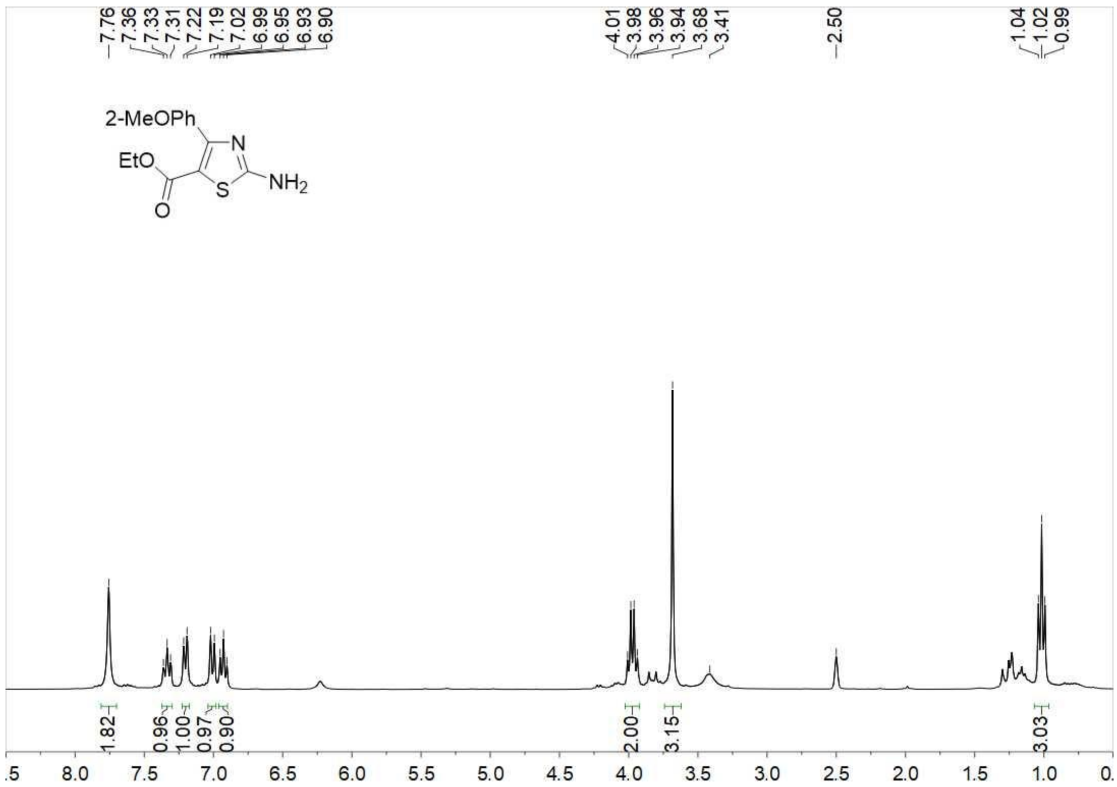

^{13}C NMR of ethyl 2-amino-4-(2-methoxyphenyl) thiazole-5-carboxylate (3q)

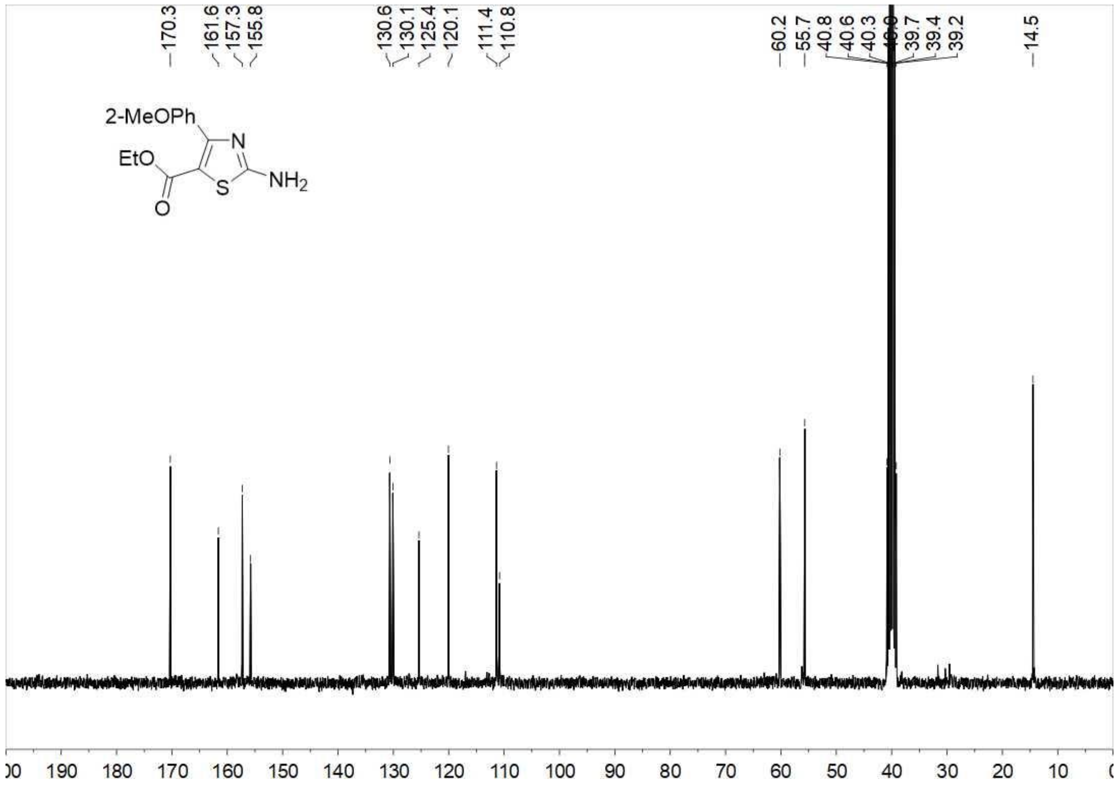

HRMS of ethyl 2-amino-4-(2-methoxyphenyl) thiazole-5-carboxylate (3q)

Peking University Mass Spectrometry Sample Analysis Report

Analysis Info

Analysis Name	FTMS-22050052_Pos_20220511_000009.d	Acquisition Date	5/11/2022 3:03:04 PM
Sample	0509-09	Instrument	Bruker Solarix XR FTMS
Comment		Operator	Peking University

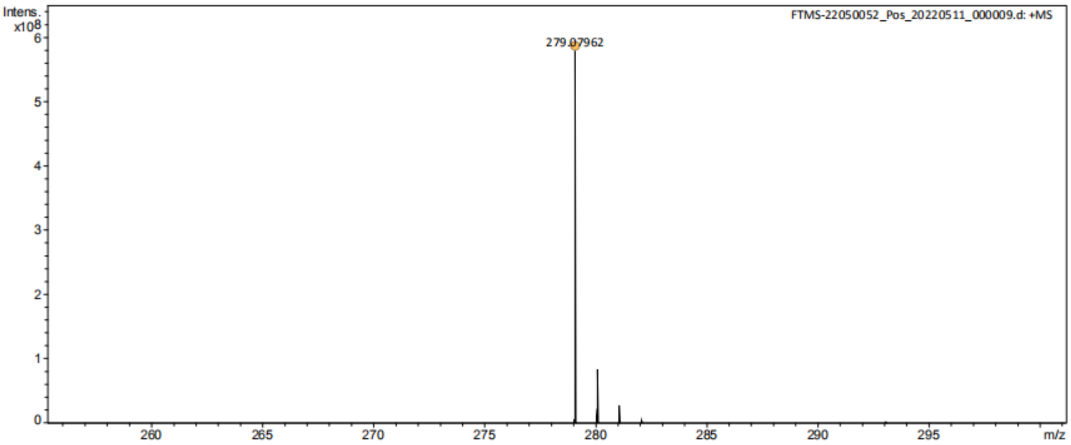

Meas. m/z	#	Ion Formula	Score	m/z	err [ppm]	Mean err [ppm]	mSigma	rdb	e⁻ Conf	N-Rule
279.079622	1	C13H15N2O3S	100.00	279.079790	0.6	0.3	4.2	10.0	even	ok

Bruker Compass DataAnalysis 5.0 printed: 5/11/2022 3:03:29 PM Page 1 of 1

^{1}H NMR of ethyl 2-amino-4-(4-fluorophenyl) thiazole-5-carboxylate (3r)

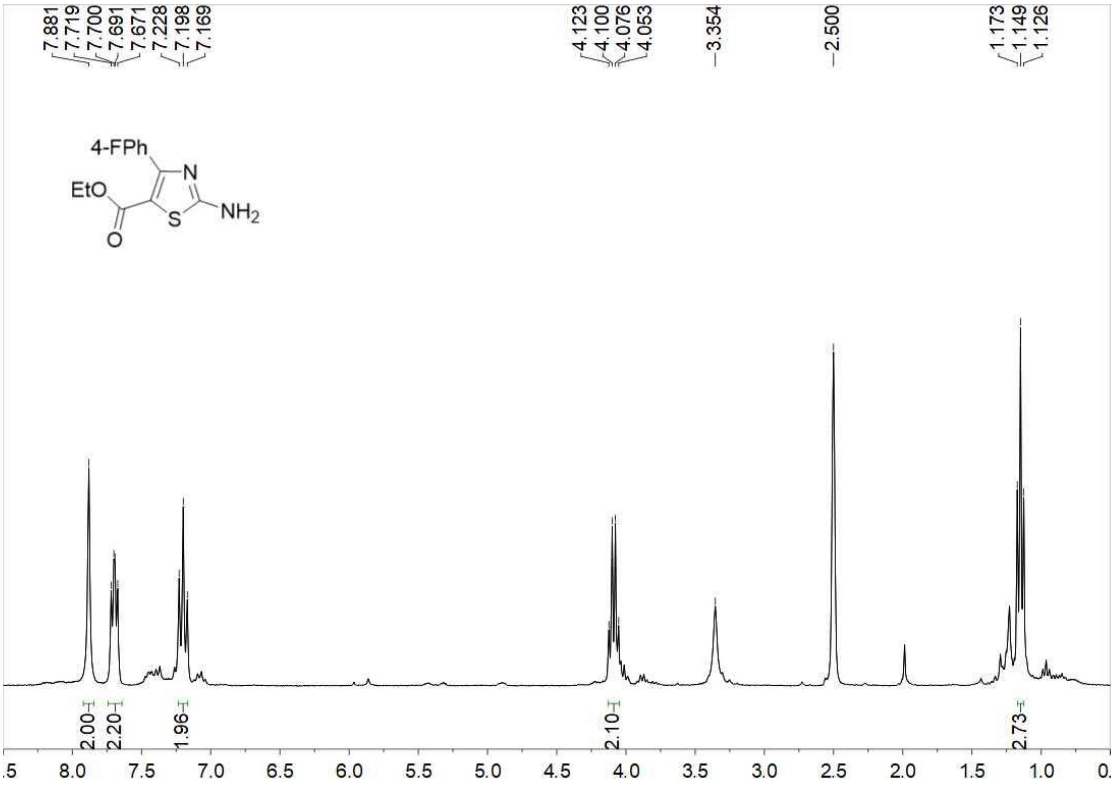

^{13}C NMR of ethyl 2-amino-4-(4-fluorophenyl) thiazole-5-carboxylate (3r)

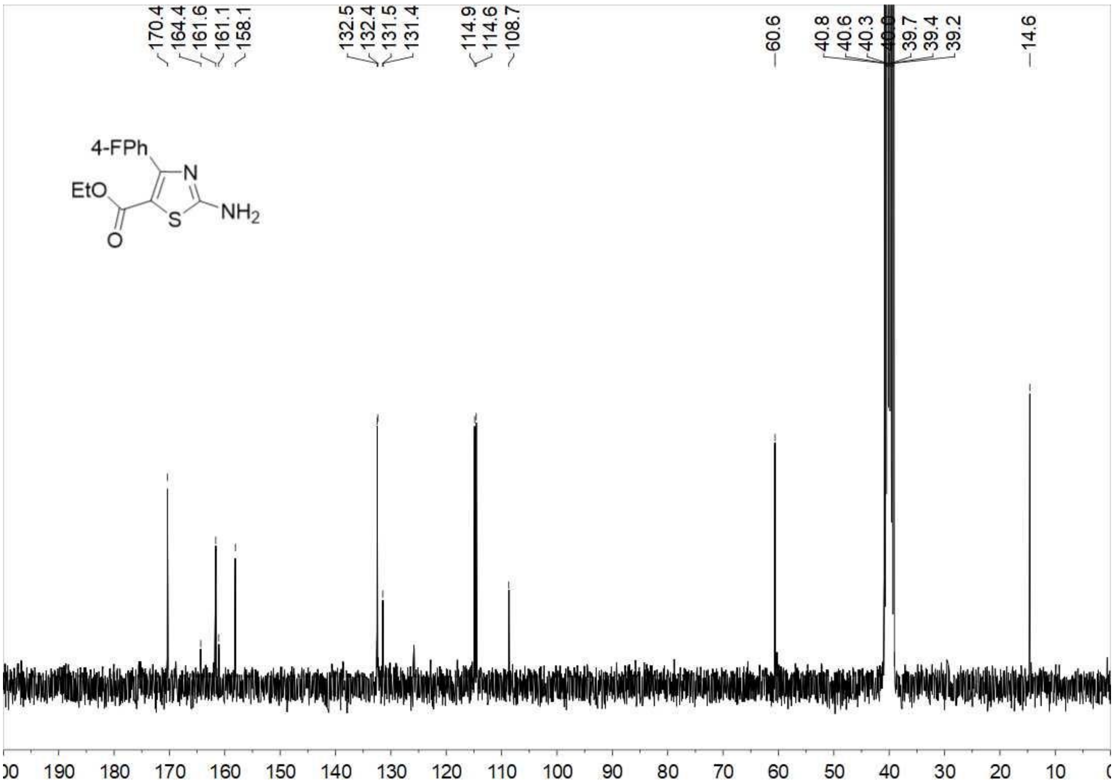

HRMS of ethyl 2-amino-4-(4-fluorophenyl) thiazole-5-carboxylate (3r)

Peking University Mass Spectrometry Sample Analysis Report

Analysis Info

Analysis Name FTMS-22050052_Pos_20220511_000006.d
Sample 0509-06
Comment

Acquisition Date 5/11/2022 2:56:44 PM
Instrument Bruker Solarix XR FTMS
Operator Peking University

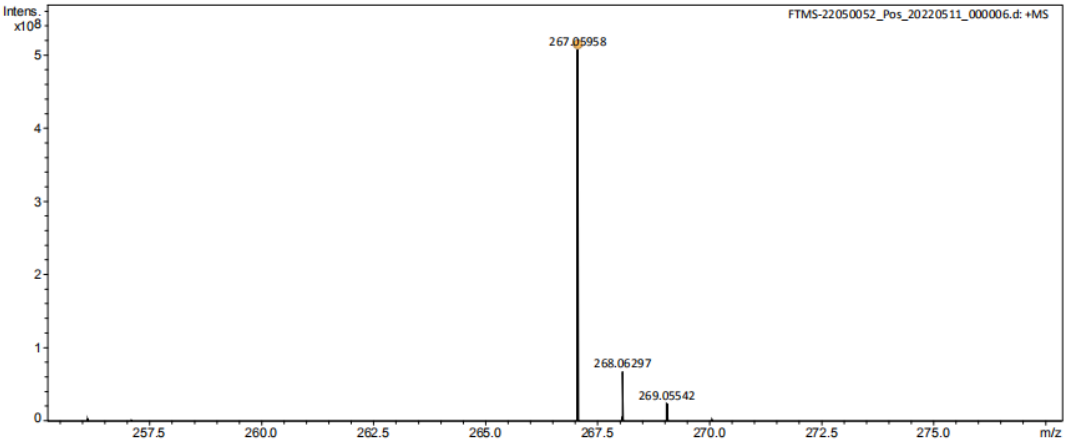

Meas. m/z	#	Ion Formula	Score	m/z	err [ppm]	Mean err [ppm]	mSigma	rdb	e⁻ Conf	N-Rule
267.059580	1	C12H12FN2O2S	100.00	267.059803	0.8	0.5	3.2	10.0	even	ok

Bruker Compass DataAnalysis 5.0 printed: 5/11/2022 2:57:13 PM Page 1 of 1

^{1}H NMR of ethyl 2-amino-4-(4-chlorophenyl) thiazole-5-carboxylate (3s)

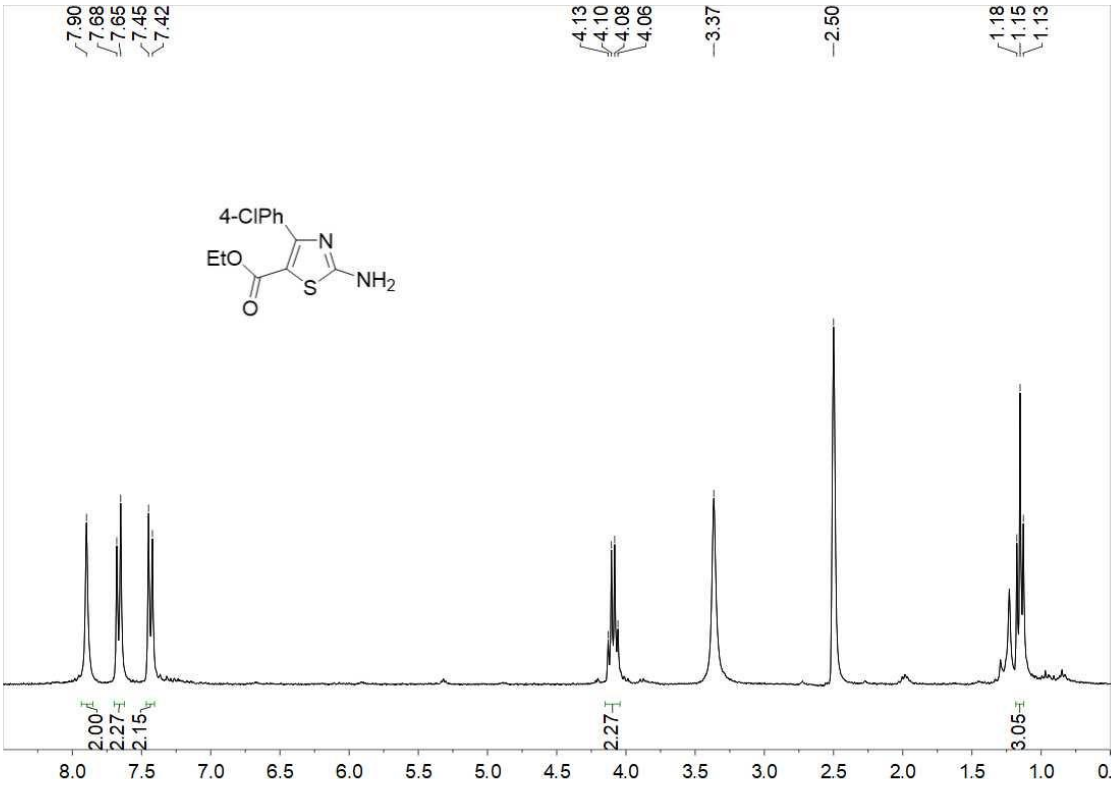

^{13}C NMR of ethyl 2-amino-4-(4-chlorophenyl) thiazole-5-carboxylate (3s)

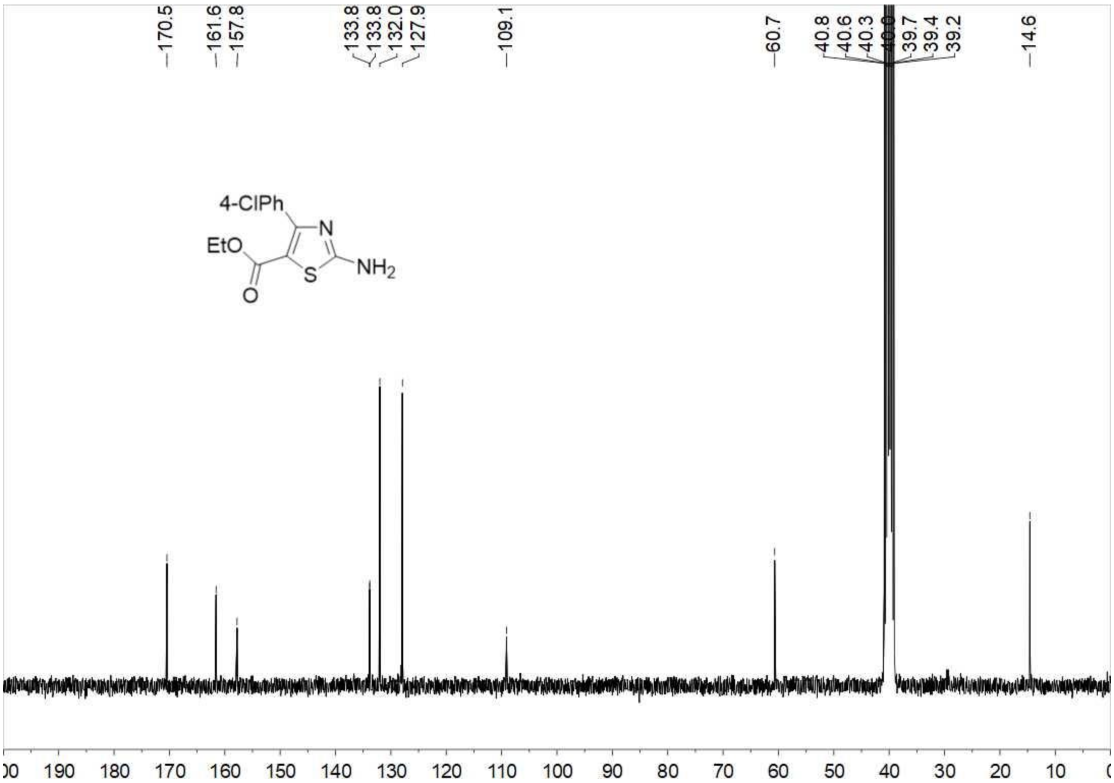

^{1}H NMR of ethyl 2-amino-4-(4-bromophenyl) thiazole-5-carboxylate (3t)

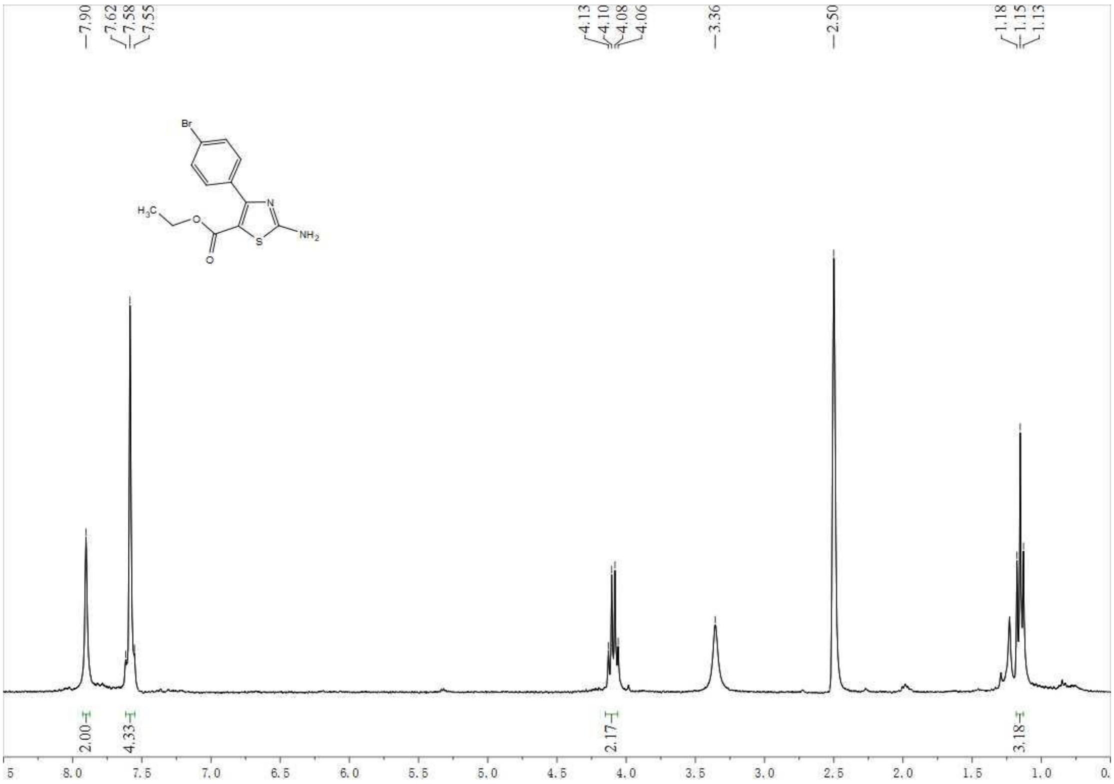

^{13}C NMR of ethyl 2-amino-4-(4-bromophenyl) thiazole-5-carboxylate (3t)

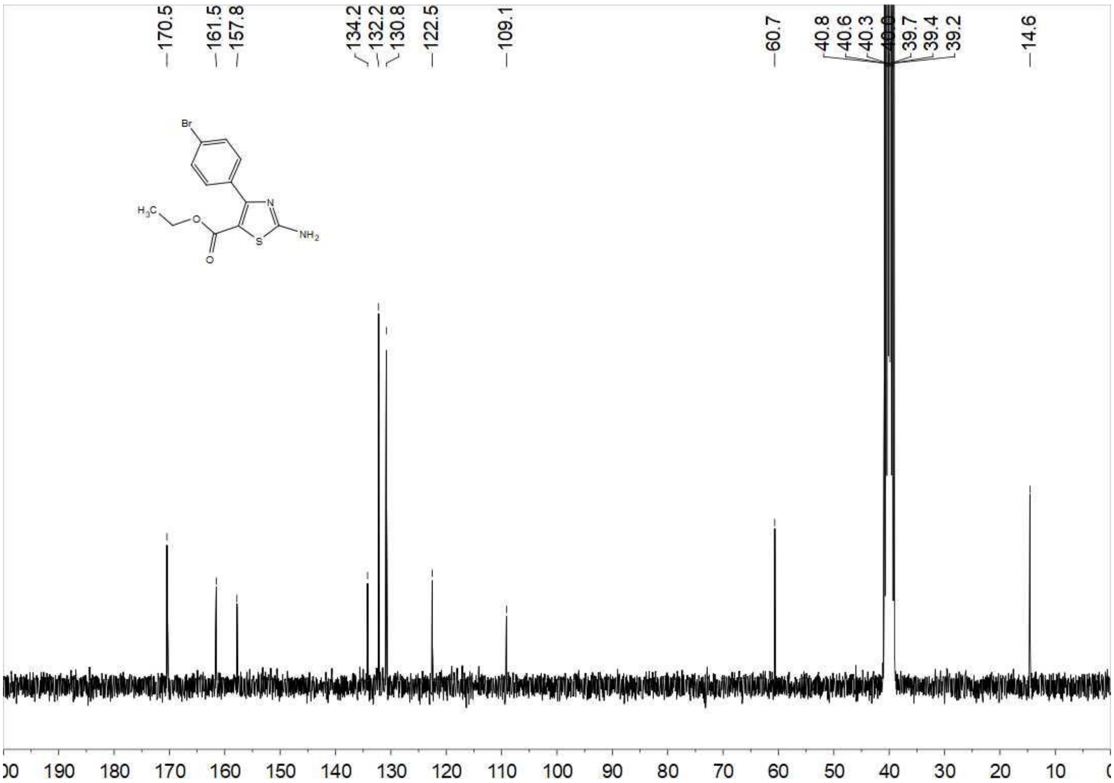

^{1}H NMR of ethyl 2-amino-4-(4-nitrophenyl) thiazole-5-carboxylate (3u)

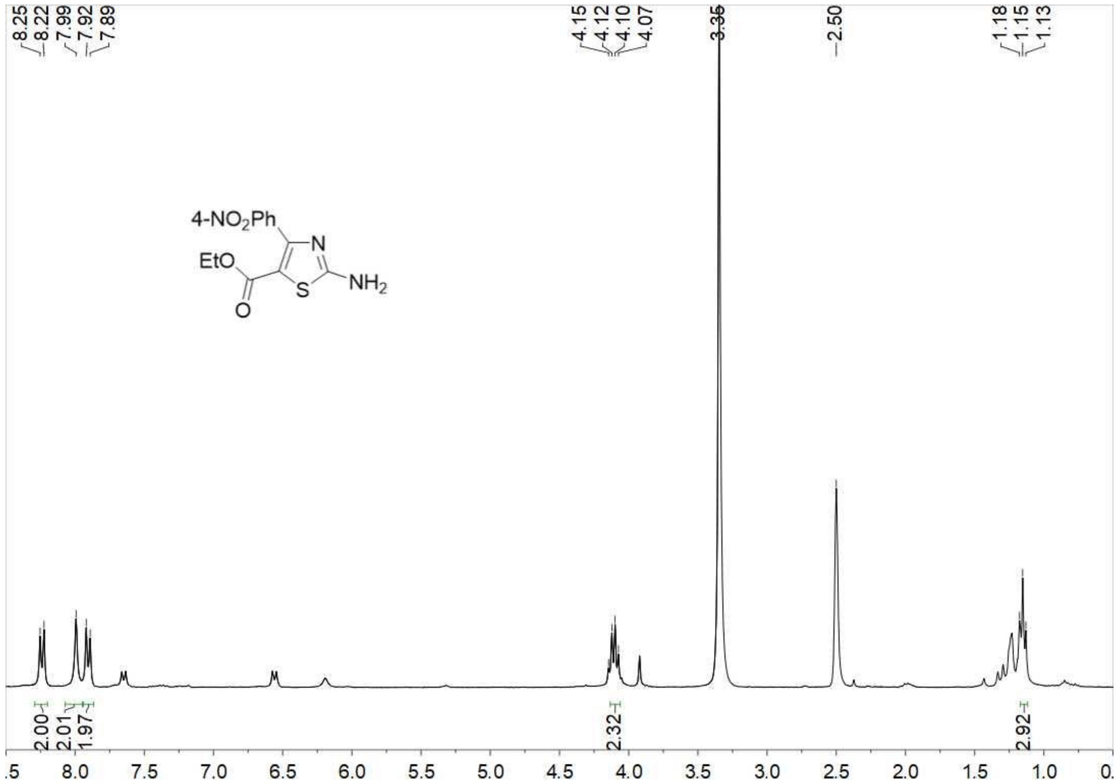

^{13}C NMR of ethyl 2-amino-4-(4-nitrophenyl) thiazole-5-carboxylate (3u)

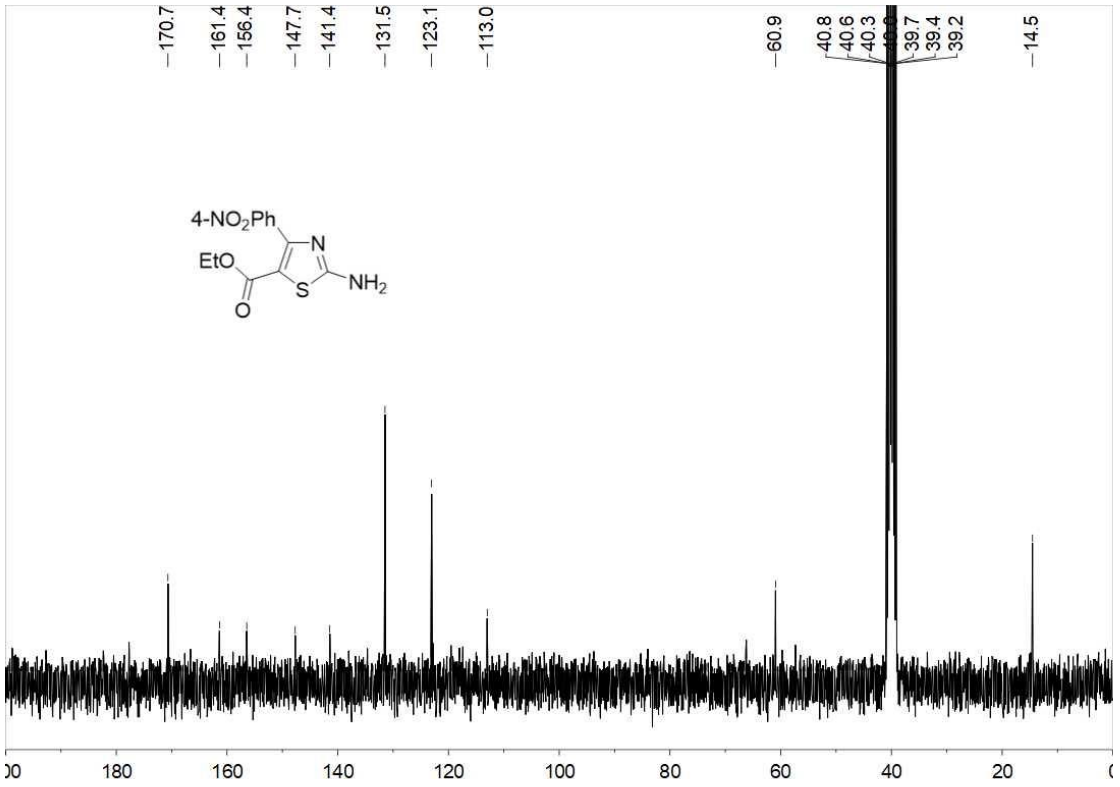

^{1}H NMR of ethyl 2-amino-4-(furan-2-yl) thiazole-5-carboxylate (3v)

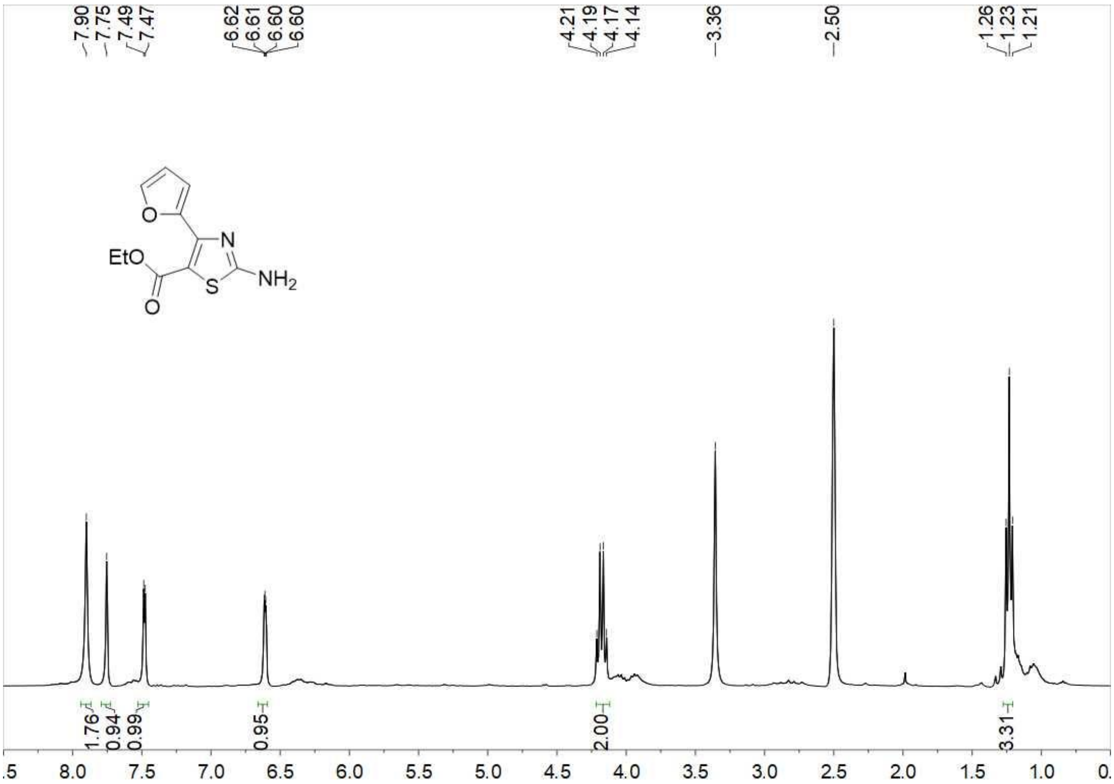

^{13}C NMR of ethyl 2-amino-4-(furan-2-yl) thiazole-5-carboxylate (3v)

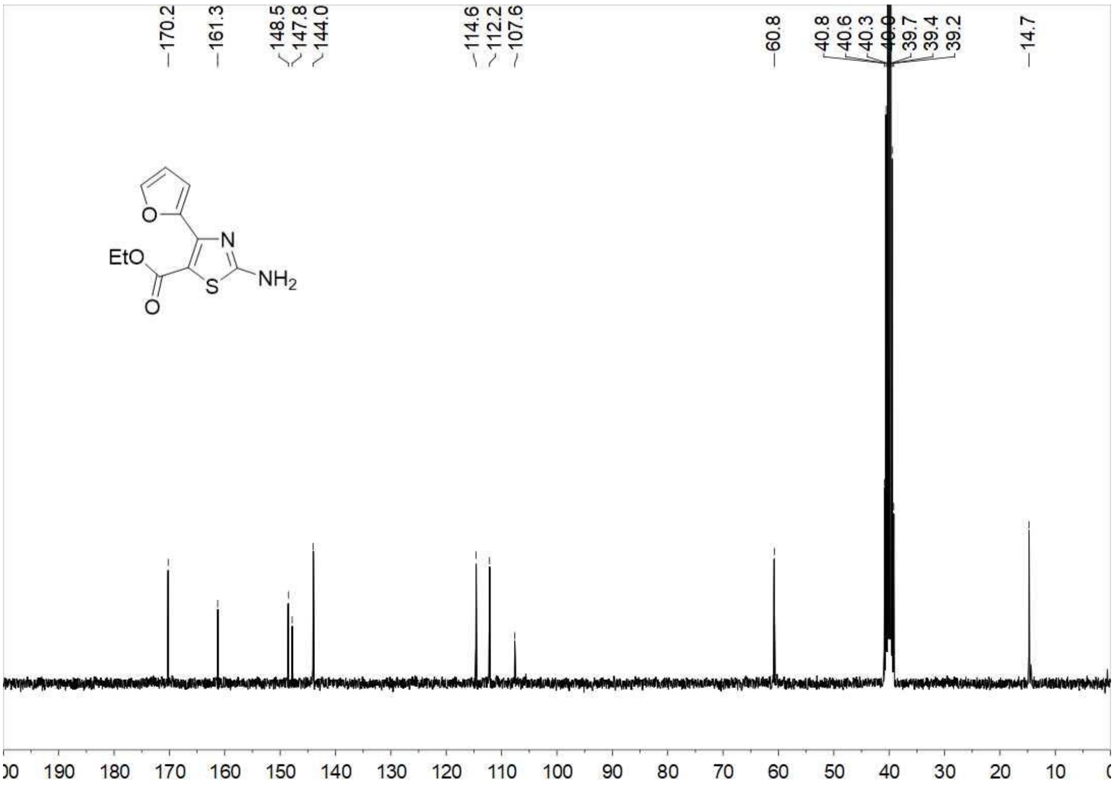

^{1}H NMR of 2-amino-N, N-diethyl-4-methylthiazole-5-carboxamide (3w)

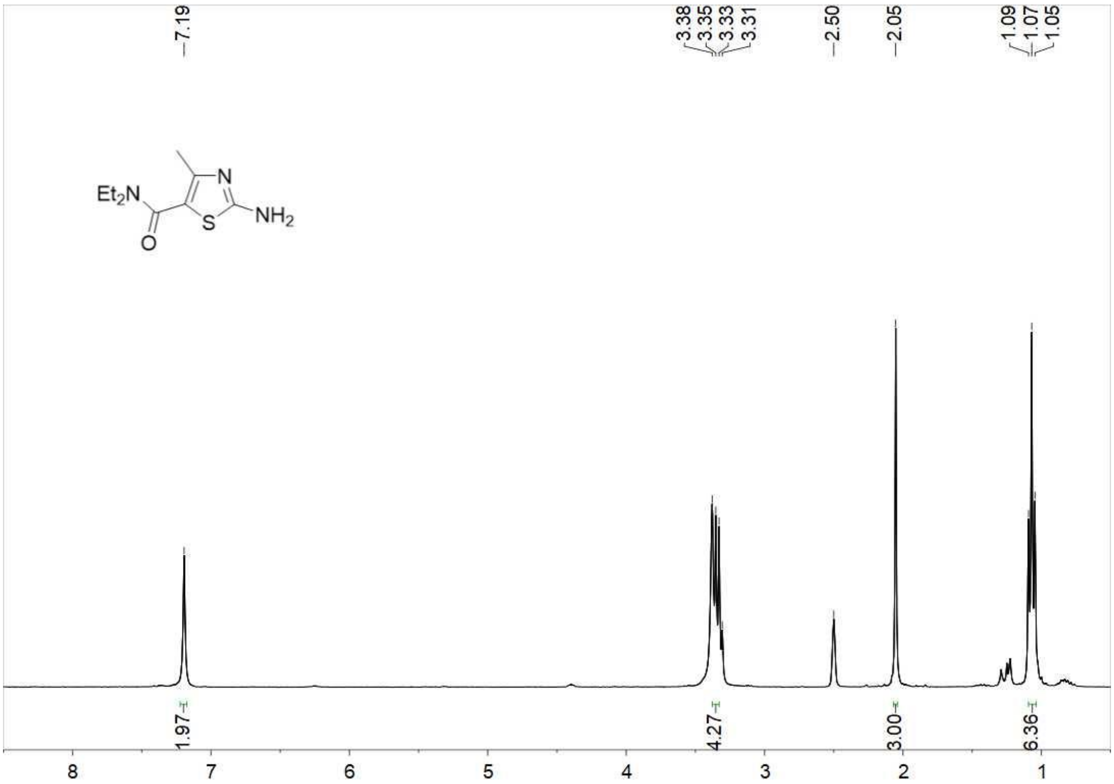

^{13}C NMR of 2-amino-N, N-diethyl-4-methylthiazole-5-carboxamide (3w)

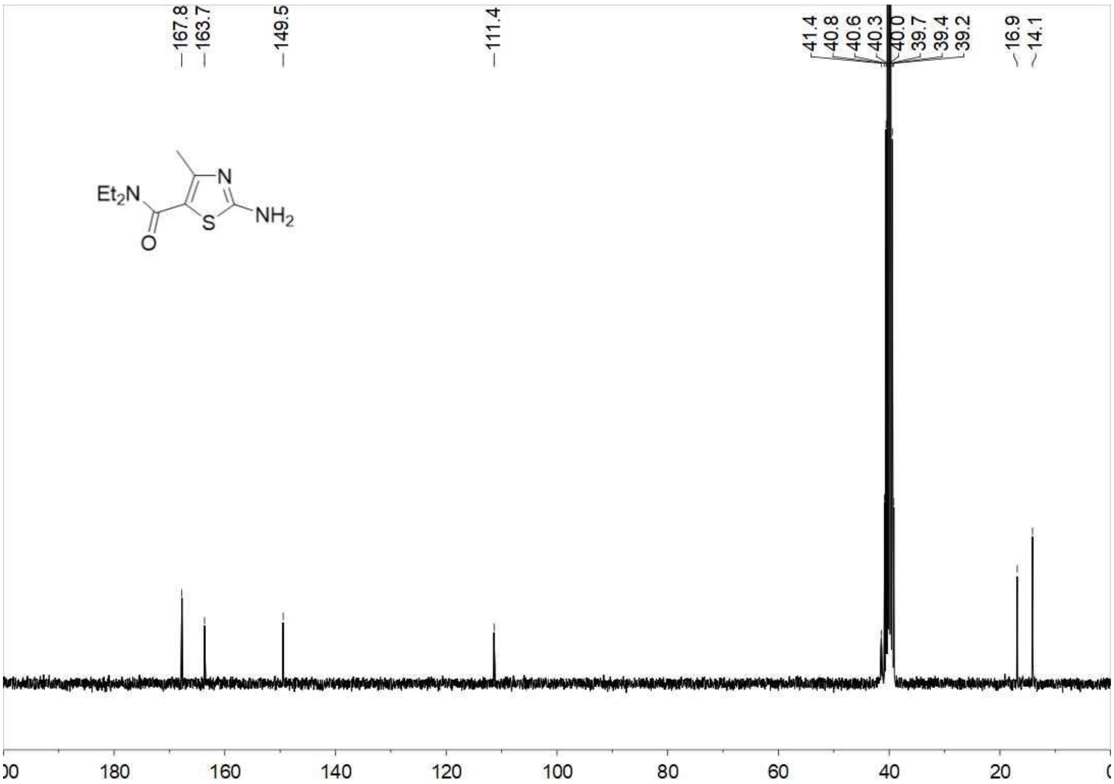

^{1}H NMR of 2-amino-4-phenylthiazole-5-carbonitrile (3x)

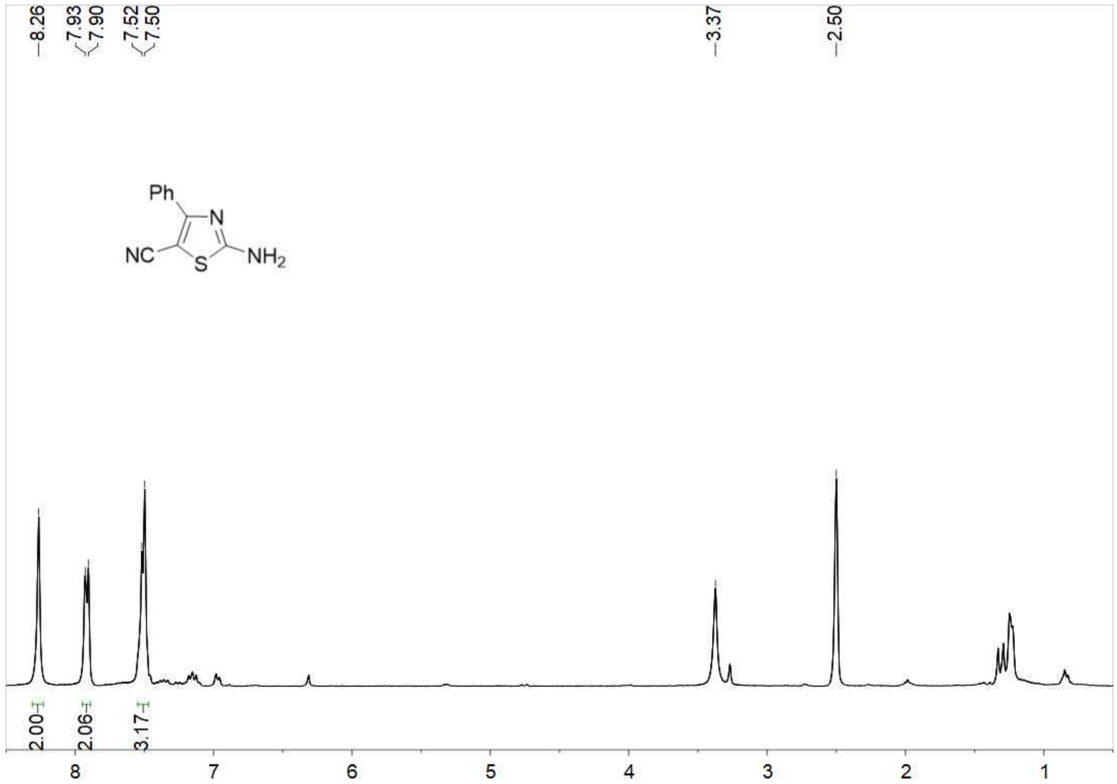

^{13}C NMR of 2-amino-4-phenylthiazole-5-carbonitrile (3x)

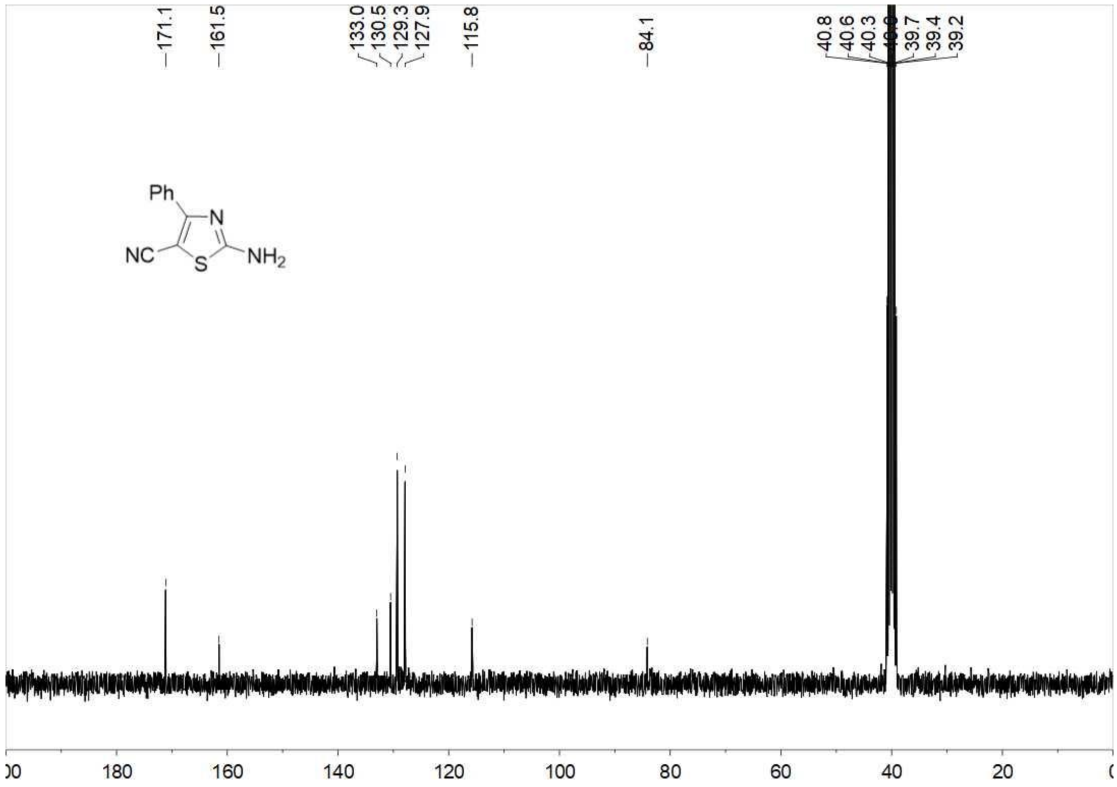

^{1}H NMR of 4-phenyl-5-(phenylsulfonyl) thiazol-2-amine (3y)

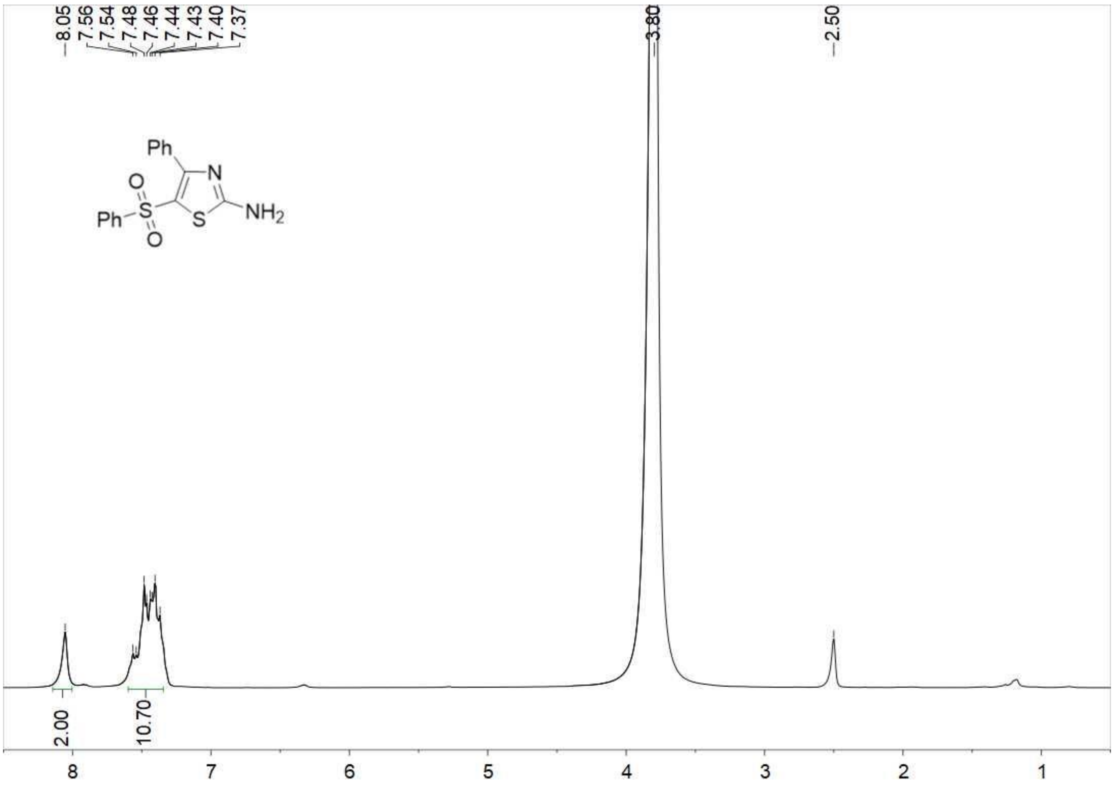

^{13}C NMR of 4-phenyl-5-(phenylsulfonyl) thiazol-2-amine (3y)

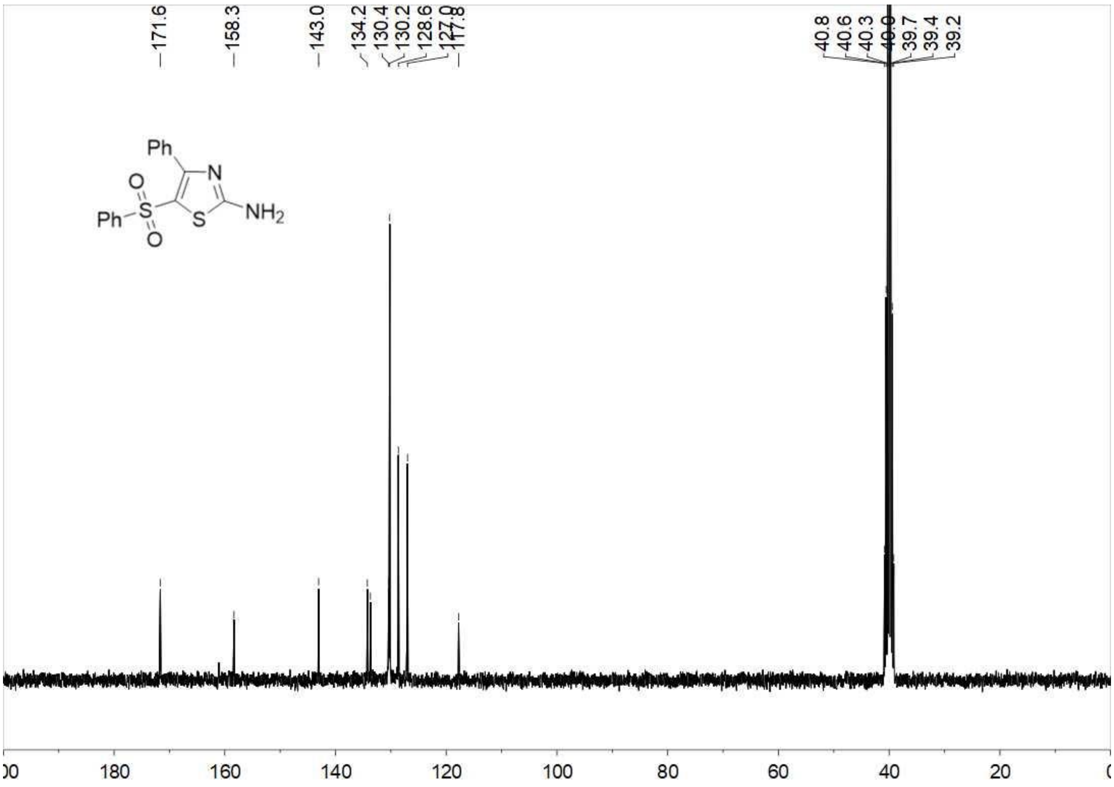

HRMS of 4-phenyl-5-(phenylsulfonyl) thiazol-2-amine (3y)

Peking University Mass Spectrometry Sample Analysis Report

Analysis Info

Analysis Name FTMS-22050052_Pos_20220511_000013.d
Sample 0509-12
Comment

Acquisition Date 5/11/2022 3:10:27 PM
Instrument Bruker Solarix XR FTMS
Operator Peking University

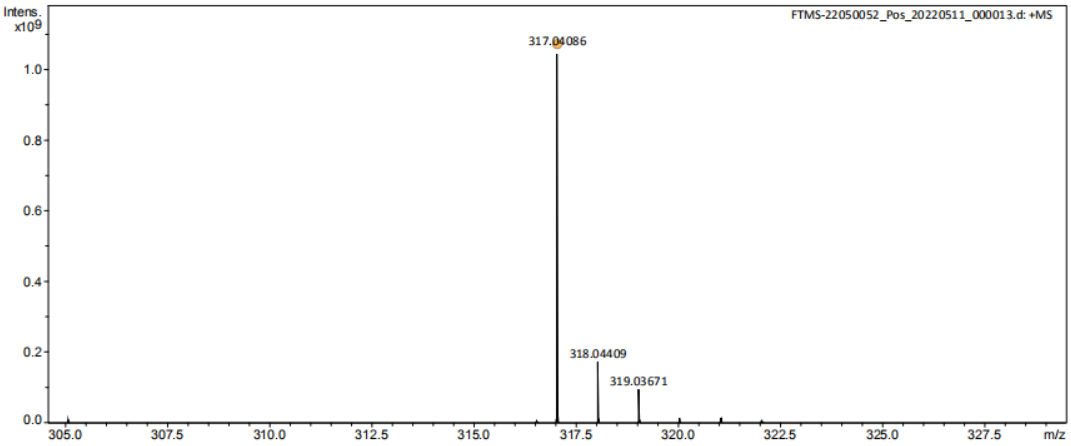

Meas. m/z	#	Ion Formula	Score	m/z	err [ppm]	Mean err [ppm]	mSigma	rdb	e⁻ Conf	N-Rule
317.040865	1	C15H13N2O2S2	100.00	317.041296	1.4	-2.1	9.7	15.0	even	ok

Bruker Compass DataAnalysis 5.0 printed: 5/11/2022 3:11:11 PM Page 1 of 1

^{1}H NMR of (2-amino-4-phenylthiazol-5-yl) (phenyl)methanone (3z)

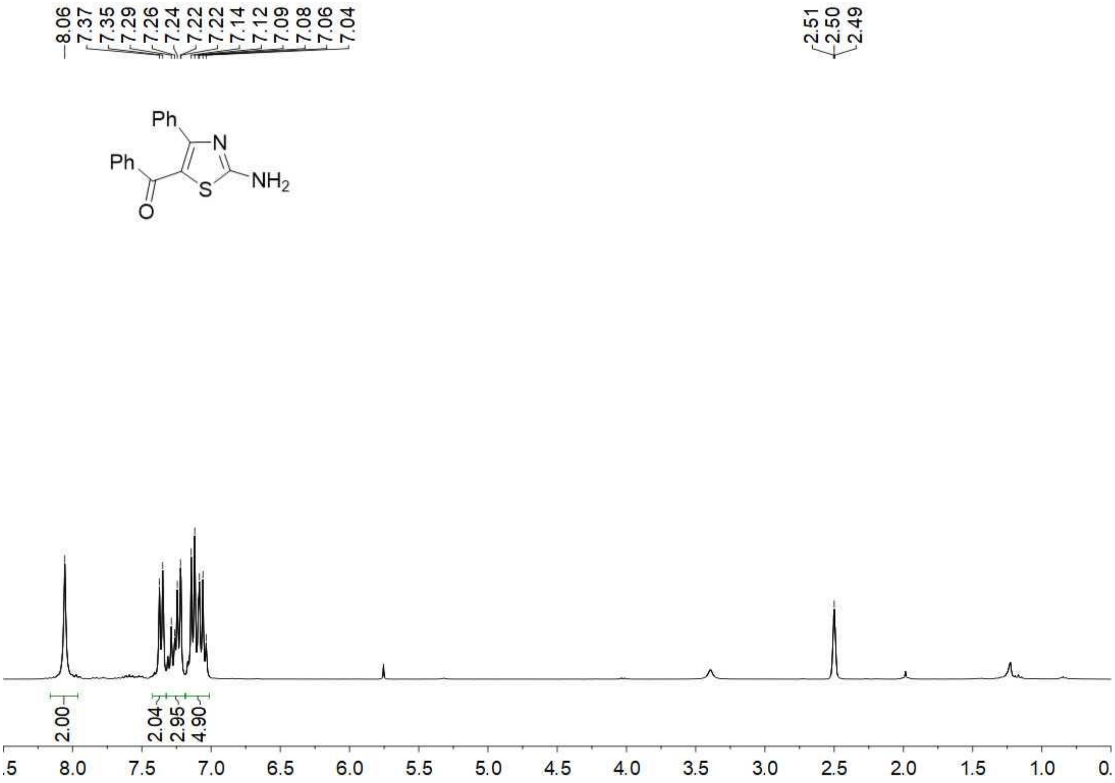

^{13}C NMR of (2-amino-4-phenylthiazol-5-yl) (phenyl)methanone (3z)

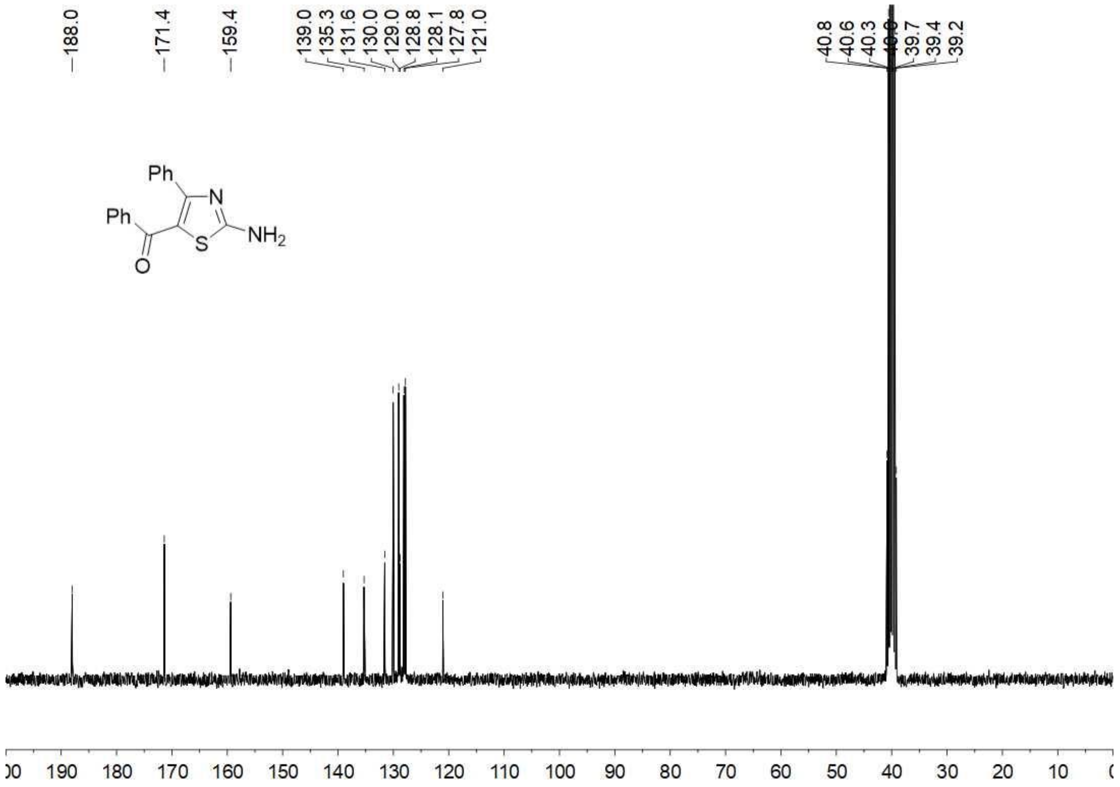

^{1}H NMR of 1-(2-amino-4-methylthiazol-5-yl)ethan-1-one (3aa)

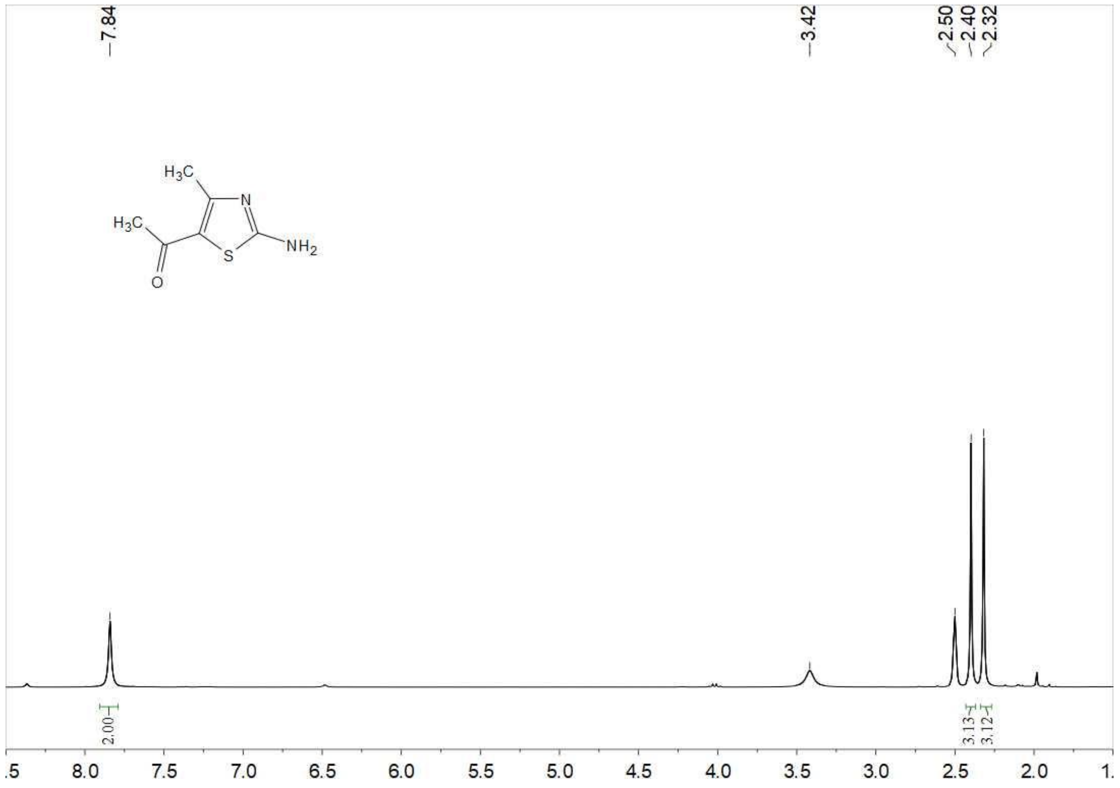

^{13}C NMR of 1-(2-amino-4-methylthiazol-5-yl)ethan-1-one (3aa)

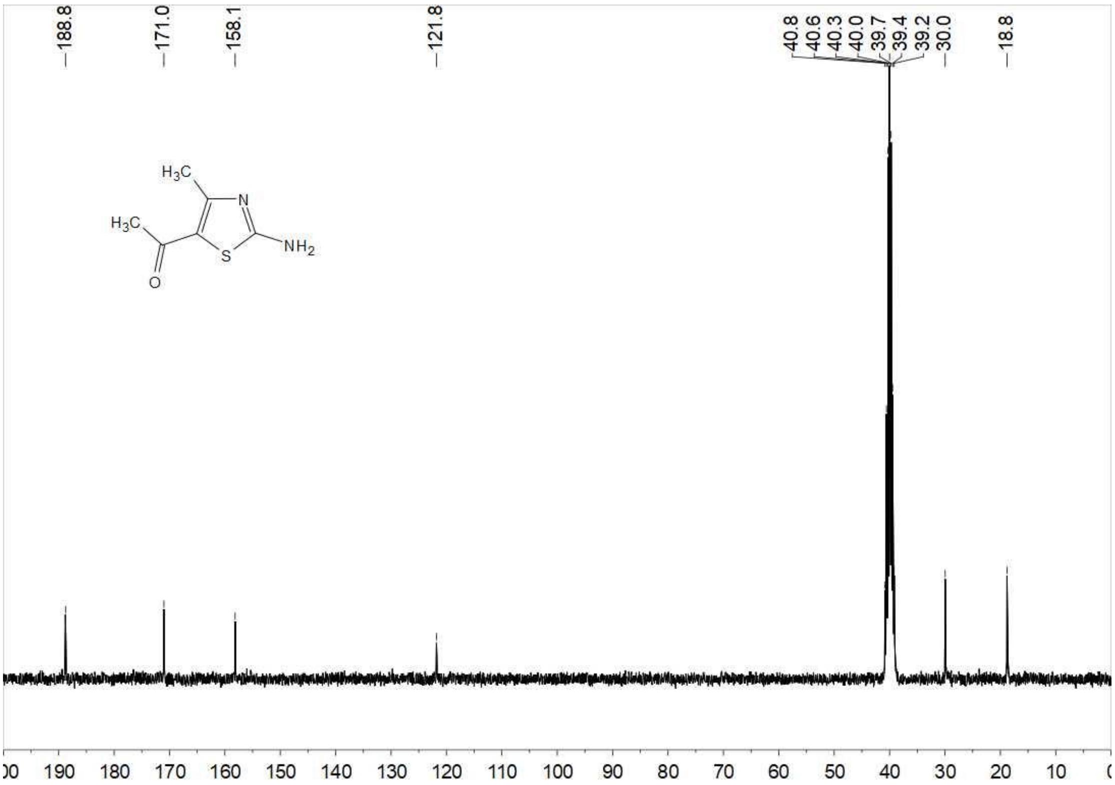

^{1}H NMR of ethyl 4-methyl-2-(methylamino) thiazole-5-carboxylate (3bb)

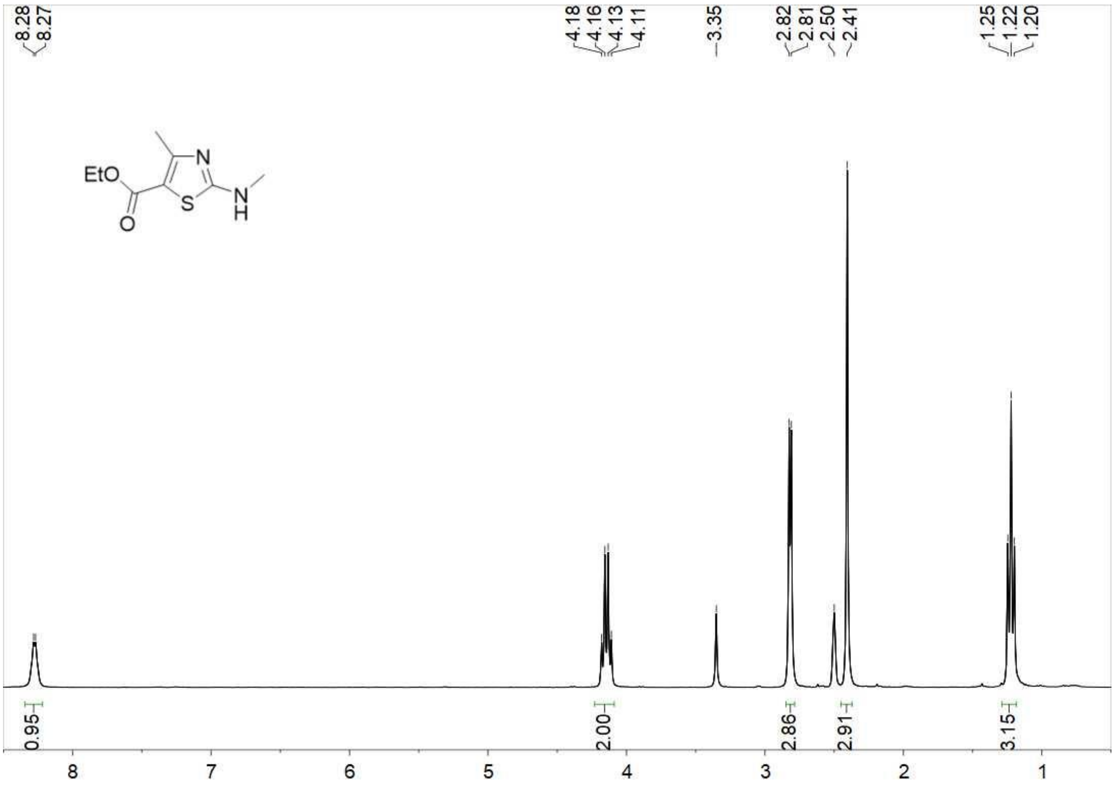

^{13}C NMR of ethyl 4-methyl-2-(methylamino) thiazole-5-carboxylate (3bb)

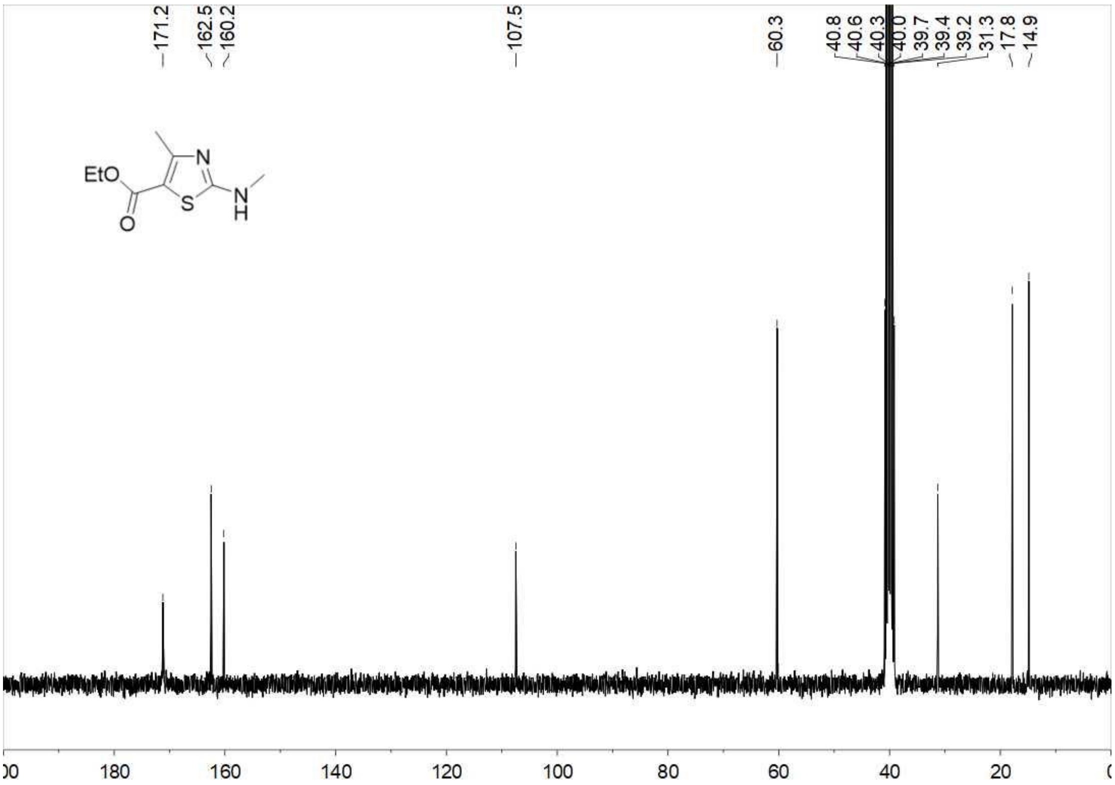

^{1}H NMR of ethyl 4-methyl-2-(phenethylamino) thiazole-5-carboxylate (3cc)

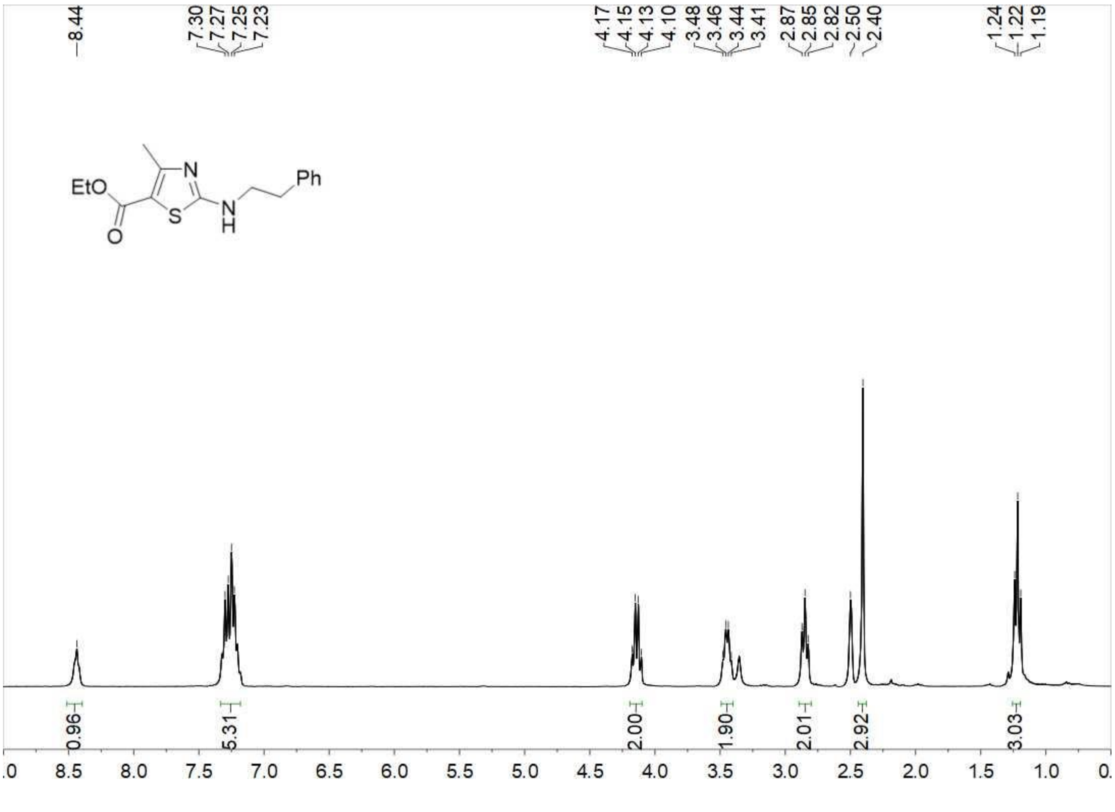

^{13}C NMR of ethyl 4-methyl-2-(phenethylamino) thiazole-5-carboxylate (3cc)

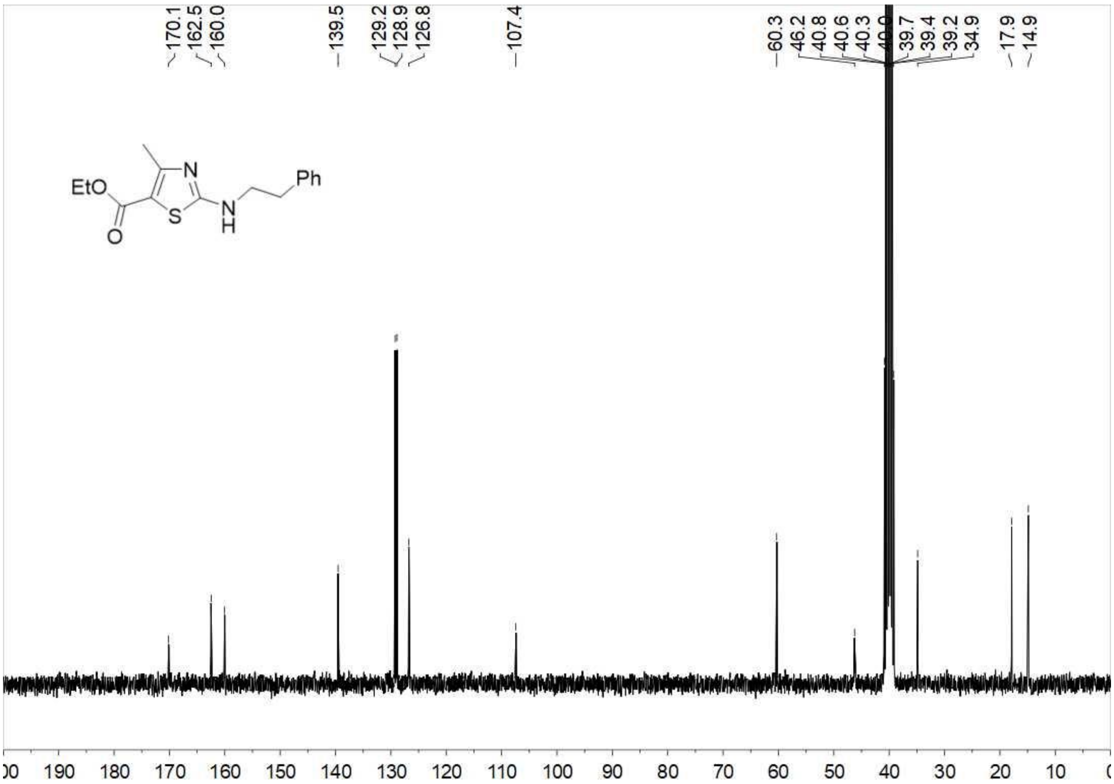

HRMS of ethyl 4-methyl-2-(phenethylamino) thiazole-5-carboxylate (3cc)

Peking University Mass Spectrometry Sample Analysis Report

Analysis Info

Analysis Name	FTMS-22050052_Pos_20220511_000011.d	Acquisition Date	5/11/2022 3:07:49 PM
Sample	0509-11	Instrument	Bruker Solarix XR FTMS
Comment		Operator	Peking University

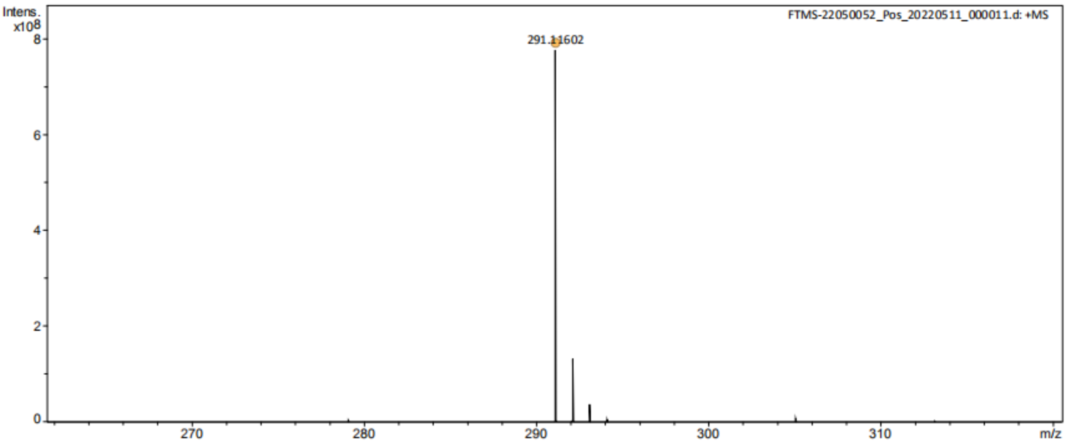

Meas. m/z	#	Ion Formula	Score	m/z	err [ppm]	Mean err [ppm]	mSigma	rdb	e⁻ Conf	N-Rule
291.116016	1	C15H19N2O2S	100.00	291.116175	0.5	-0.0	6.9	10.0	even	ok

Bruker Compass DataAnalysis 5.0 printed: 5/11/2022 3:08:19 PM Page 1 of 1
